# Rib microstructure in thunniform ichthyosaurs and toothed whales

**DOI:** 10.7717/peerj.21486

**Published:** 2026-07-07

**Authors:** Jørgen Krøglid, Mathieu Gabriel Faure-Brac, Lene Liebe Delsett

**Affiliations:** Natural History Museum, University of Oslo, Oslo, Norway

**Keywords:** Ichthyosaurs, Odontocetes, Histology, Microanatomy, Microstructure, *Palvennia*, *Keilhauia*, *Phocoena*, *Delphinapterus*, Ribs

## Abstract

The inner microstructure of aquatic amniote ribs can reveal adaptations to physiology and locomotion, but are relatively poorly known for ichthyosaurs, especially ophthalmosaurid taxa whose body shape converges to modern odontocetes. In this study, we describe the microstructure of ribs and gastralia from two Late Jurassic ichthyosaurs (†*Palvennia hoybergeti* and †*Keilhauia* sp.) and compare them to extant odontocetes (*Delphinapterus leucas* and *Phocoena phocoena*). Ribs were sampled at proximal, midshaft, and distal locations to assess the potential for reliable growth marks, and quantitative microanatomical values were collected to enable a comparison to other ichthyosaurs. The ichthyosaur ribs preserve alternating vascular and avascular bands resulting from localized growth variation, but no unambiguous growth marks, whereas gastralia seem more promising for skeletochronology. The toothed whale ribs preserve no growth marks. Ichthyosaur skeletal elements often have a spongy internal structure without a thick cortex, but the ophthalmosaurid ribs in this study have relatively compact bone throughout, compared to the cetaceans, something that is possibly an adaptation to counter ballast the buoyancy of the lungs.

## Introduction

Ichthyosaurs were secondarily aquatic reptiles that dominated the world’s oceans through much of the Mesozoic Era, with a fossil record spanning from the Early Triassic (~250 Ma) to their final demise in the early Late Cretaceous (~93 Ma) (Bardet, 1992; Fischer et al., 2016; Kear et al., 2023). Ichthyosaurs are among the few aquatic amniotes to acquire a fully aquatic lifestyle in the open ocean, matched only by that of modern cetaceans. In both groups, the skeleton underwent extreme modifications compared to their land-dwelling ancestors (Kelley & Pyenson, 2015), and consequently, these groups are important for understanding convergent evolution of secondarily aquatic adaptations (*e.g.*, Delsett et al., 2023; Sander et al., 2021).

Observed variation in skeletal elements result from physical forces acting on them, their adaptive role, and their phylogenetic history (de Buffrénil & Quilhac, 2021; Seilacher, 1970). Variation is evident not only in gross skeletal morphology but also in the internal microstructure, and different bone tissues relate to factors such as biomechanical loading, body size, ecology, growth, and ontogeny (Chinsamy, 2023; Hand et al., 2025; Houssaye, Nakajima & Sander, 2018; Wilson, 2023). Thus, descriptions of bone microstructure can provide important biological inferences. Microanatomical descriptions address the internal architecture, such as cortical thickness, medullary cavity size, and trabecular organization (de Buffrénil et al., 2021). Histology, on the other hand, examines the characteristics and organization of the bone tissue itself, including the vascular pattern, cellular morphology, and the type of extracellular matrix (de Buffrénil et al., 2021). The primary distinction between these fields lies in their level of magnification. Histology involves higher magnification and focuses on finer structural details that appear invisible to the eye, whereas microanatomy operates as an intermediate scale between anatomy and histology, making it relevant to both disciplines (de Buffrénil et al., 2021; Francillon-Vieillot et al., 1990). Bone microanatomy in aquatic amniotes has generated significant interest, most commonly focusing on whether bones are compacted or cancellous, *i.e.*, the prevalence of osteosclerosis, pachyosteosclerosis and osteoporosis (*e.g.*, de Buffrénil & Mazin, 1990; de Ricqlès, 1976; Houssaye, 2009; Houssaye, 2013; Houssaye & Fish, 2016).

Previous research on ichthyosaurs (*e.g.*, Anderson et al., 2018; de Buffrénil & Mazin, 1990; Delsett et al., 2025; Houssaye, Nakajima & Sander, 2018; Houssaye et al., 2014; Kiprijanoff, 1881; Kolb, Sanchez-Villagra & Scheyer, 2011; Sander, 2021; Talevi & Fernández, 2012) suggest that their skeletal elements exhibit the following microstructural characteristics: (1) a predominantly cancellous bone organization, typically lacking a free medullary cavity in long bones; (2) the overall spongy nature results in a relatively thin cortical region in long bones and vertebral centra; (3) scarcity of compact bone, and thus of growth marks, presenting challenges for skeletochronology; and (4) skeletal elements from different taxa exhibiting woven-parallel complexes. The latter is an indication of rapid growth rate (Amprino, 1947; Ferretti & Palumbo, 2021; Marotti, 2010) that supports the idea that ichthyosaurs had elevated body temperatures, with an endothermy-like metabolism (Anderson et al., 2018; Delsett et al., 2025; Houssaye, Nakajima & Sander, 2018; Houssaye et al., 2014; Nakajima, Houssaye & Endo, 2014). Despite this, our knowledge about ichthyosaur physiology and growth remains limited. Microstructural data from ribs might shed light of these topics.

Ribs are curved, elongated bones that articulate with vertebral centra and play a key role in supporting the internal organs, particularly those of the respiratory system. In ichthyosaurs, in contrast to cetaceans, ribs are present along the entire presacral region of the vertebral column, and in addition, they possess gastralia (belly ribs), which reinforce the body wall (McGowan & Motani, 2003), and are in other extinct reptiles hypothesized to contribute to ventilation (Carrier & Farmer, 2000; Claessens, 2004). Studying ribs offers several advantages: Although each rib represents a small portion of the skeleton, the entire ribcage contributes significantly to overall skeletal mass, and thickened and compacted ribs are well-known as an adaptation for ballast, for instance in slow-moving sirenians (de Buffrénil et al., 2010). Rib microanatomy, especially the compactness, thus might reflect ecology and locomotion (Canoville, de Buffrénil & Laurin, 2016). Ribs are generally similar in structure across amniote taxa, which makes them well-suited for comparative studies. Lastly, ribs are frequently preserved due to their abundance, which also makes it easier to obtain permission to destructively sample for histological analysis.

Rib microstructure in ichthyosaurs has been figured and/or described for a handful of Triassic ichthyosaurs (de Ricqlès, Taquet & de Buffrénil, 2009; Kolb, Sanchez-Villagra & Scheyer, 2011; Nakajima, Houssaye & Endo, 2014; Sander & Faber, 2003), some thunniform Early and Middle Jurassic taxa (Anderson et al., 2018; de Ricqlès, Taquet & de Buffrénil, 2009; Enlow & Brown, 1957; Fernández & Talevi, 2013; Seitz, 1907; Wallstedt et al., 2024), and for a number of Middle Jurassic-Cretaceous ophthalmosaurids (Fernández & Talevi, 2013; Houssaye & Fish, 2016; Houssaye et al., 2014; Kiprijanoff, 1881; Martill, 1987; Seitz, 1907; Talevi & Fernández, 2012; Talevi, Fernández & Salgado, 2012). The latter group likely resembled modern toothed whales in locomotion and ecology, as they were pelagic predators with a similar body shape, and are thus the focus of this comparative study.

Here, we investigate physiological data recorded in the microstructure of ribs and gastralia to answer two questions: First, did ichthyosaurs have a high and consistent growth rate? Second, as cetaceans are commonly seen as the modern analogues of ichthyosaurs, do their rib microanatomy and histology converge?

To answer these questions, we first describe the rib microanatomy and histology of two Late Jurassic ophthalmosaurid ichthyosaurs. A common challenge in studies on ichthyosaur ribs is the lack of precise information on sampling location along the rib shaft, which is only provided explicitly in a few studies (Anderson et al., 2018; Nakajima, Houssaye & Endo, 2014; Sander & Faber, 2003; Talevi & Fernández, 2012; Wallstedt et al., 2024). This knowledge gap is significant, as these studies and work on other groups demonstrate that microanatomy and histology vary depending on the sampling position (Gray et al., 2007; Houssaye et al., 2015; Klein, Canoville & Houssaye, 2019; Waskow & Sander, 2014). Another notable gap is the scarcity of studies incorporating longitudinal sections (Kiprijanoff, 1881; Talevi & Fernández, 2012). Thus, to better understand the gross architecture and detect potential intra-elemental variation, ribs in this study were systematically sampled at three locations along the shaft, and in both directions. In addition to histological data, we acquire quantitative microanatomical data, allowing for standardized assessment of bone compactness profiles.

Secondly, we assess the potential for identifying reliable growth marks for use in skeletochronology. Growth marks have only been confidently identified in †*Mixosaurus*, where gastralia were found to be especially informative due to their compact microanatomy (Kolb, Sanchez-Villagra & Scheyer, 2011). Thus, we sampled gastralia from the ichthyosaur specimens in addition to the ribs, contributing to the limited existing histological work on these elements (Anderson et al., 2018; Kolb, Sanchez-Villagra & Scheyer, 2011). While ichthyosaurs often have cancellous bones, some studies noted increased bone mass in the ribs of thunniform ichthyosaurs, which might increase the possibility for preservation of primary bone and thus potentially growth marks in the ribs of these taxa (Anderson et al., 2018; Talevi & Fernández, 2012; Wallstedt et al., 2024).

Thirdly, we investigate convergent microstructural features between ichthyosaurs and cetaceans. Two modern odontocetes of approximately the same body size as the ichthyosaurs, *Phocoena phocoena* (harbour porpoise) and *Delphinapterus leucas* (beluga), were sampled. While microanatomical characteristics of ribs from these species have been mentioned (Canoville, de Buffrénil & Laurin, 2016), their histological features are previously undocumented.

## Materials and Methods

### Abbreviations

NHMO—DMA: Mammal Collection, Natural History Museum, University of Oslo, Norway.

PMO—Paleontological Collection, Natural History Museum, University of Oslo, Norway.

### Materials

**Figure 1:**
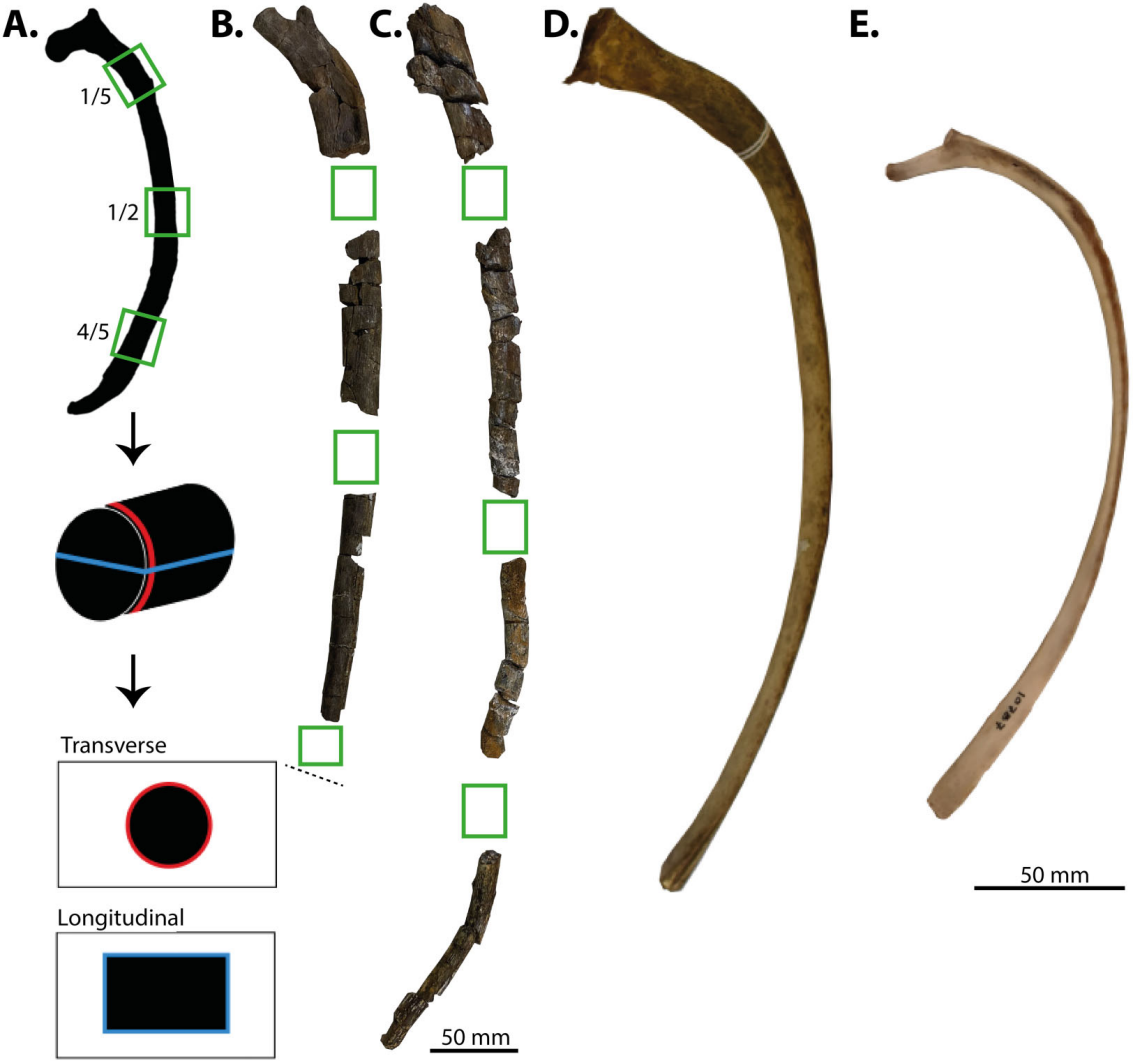
Sampling of ribs for microstructural data. (A) Sampling process. The sites along the rib where samples were taken, the method of cutting the rib pieces for analysis, and the resulting thin sections. One transverse and one longitudinal section was made for each site. (B–C) Ichthyosaur ribs used in this study, with sampling sites. (B) PMO 222.667, †*Keilhauia* sp.; dashed line indicates the missing distal rib portion. (C) PMO 222.669, †*Palvennia hoybergeti*. (D) and (E) Cetacean ribs used in the study. (D) *Phocoena phocoena* NHMO-DMA-42918. (E) *Delphinapterus leucas* NHMO-DMA-32051.

Two ichthyosaur specimens excavated in 2011 from the Late Jurassic-Early Cretaceous Slottsmøya Member Lagerstätte on Spitsbergen, Norway (Delsett et al., 2016, 2018, 2019) were sampled for this study (Figs. 1A–1C). Permit for the excavation (RIS ID: 4760) was given by the governor of Svalbard. We use the taxonomic names †*Keilhauia* sp. Delsett et al., 2017 for PMO 222.667 (Delsett et al., 2019) and †*Palvennia hoybergeti* Druckenmiller et al., 2012 for PMO 222.669 (Delsett et al., 2018), but see Zverkov & Prilepskaya (2019) for a discussion about this taxonomic assignment. Both specimens were skeletally mature at the time of death (Delsett et al., 2018, 2019), with an estimated body length of approximately 4.5 m (PMO 222.667) and 5.0 m (PMO 222.669) (Delsett, 2018). In addition to size and overall ossification of skeletal elements, the ontogenetic assessment for †*Keilhauia* sp. (PMO 222.667) was based on the well-developed crests and surface of the humerus, and clavicle-interclavicle fusion (Delsett et al., 2019), and for †*Palvennia hoybergeti* (PMO 222.669) on the robustness and size of the pterygoid quadrate ramus wings and basipterygoid processes, well-developed distal humeral facets (Delsett et al., 2018, 2019). Their sex is undetermined.

For comparison, two extant cetacean ribs from the mammal collection of the Natural History Museum in Oslo were sampled. NHMO-DMA-42918, a female harbour porpoise *Phocoena phocoena* (Linnaeus, 1758) was collected from Vestfold, Norway, in 2000, and NHMO-DMA-32051, a beluga *Delphinapterus leucas* (Pallas, 1776) was collected from an undisclosed locality in Greenland in 1900 (Figs. 1D, 1E). Based on size and epiphyseal fusion, the harbour porpoise is skeletally mature, whereas the beluga is close to reaching skeletal maturity. Thus, they are well-suited for comparison with the ichthyosaur specimens. As their body size is comparable to the ichthyosaurs (harbour porpoise 1.5–1.9 m; beluga 3.0–4.8 m (Carwardine, 2022)), body size effects on microstructural variation (Canoville, de Buffrénil & Laurin, 2016; Houssaye, Nakajima & Sander, 2018) are negligible. The sample size for this study is small, and further studies with additional specimens will make inferences more robust with regard to physiology, evolutionary convergence, and intraspecific variability.

Permission for the destructive sampling of the museum specimens was obtained. To minimize the impact of these irreversible actions, all specimens were photographed and documented using a Nikon D850 camera prior to sampling. Additionally, the cetacean rib pieces were CT scanned using a Nikon XT H225 ST micro-computed tomograph, and stored as digital 3D models, which were later used to create 3D-printed replicas with an Ultimaker S3 printer. These printed models can serve as stand-ins for the removed rib sections in the collection and if the specimens are exhibited in the future.

### Methods

We systematically sampled trunk (“thoracic” in whales, “anterior dorsal” in ichthyosaurs;) ribs from the ophthalmosaurid ichthyosaur specimens and extant odontocetes, and gastralia from the two ichthyosaurs. Well-preserved ribs were selected from vertebrae T10, T11, or T12 in the odontocetes and the equivalent position in the ichthyosaurs. Comparing ribs from the same part of the axial skeleton is important as function differ between regions, for instance the involvement in breathing and locomotion (Fig. 1). Rib length was 49 cm for each of the ichthyosaurs (PMO 222.667 and PMO 222.669), 30 cm for the porpoise (NHMO-DMA-42918) and 35 cm for the beluga (NHMO-DMA-32051). Additionally, gastralia pieces were sampled from both ichthyosaur specimens. The microanatomy and histology of ribs and gastralia are described and compared qualitatively, and compactness parameters were collected and compared quantitatively.

Three different locations—proximal, midshaft, and distal—were sampled from each rib (Figs. 1B–1E). For the cetacean specimens, the total length of the rib was measured from the proximal to the distal end. The desired sampling locations were marked at 1/5 (proximal), 1/2 (midshaft), and 4/5 (distal) of the total rib length. Cuts were made 0.8 cm on either side of the marked points, using a fixed wet abrasive saw with a diamond blade. The rib pieces were placed in small containers submerged in ethanol and left for 2–3 days, dried before being submerged in acetone (propan-2-one), and left for another 2–3 days to dissolve fat and collagen.

The ribs of the ichthyosaur specimens were fragmented when excavated and later reassembled with glue, and measured in the same manner as the cetacean specimens. The rib of PMO 222.667 was missing the distalmost part (Fig. 1B). Based on other ribs from the same individual, the missing piece is estimated to be 7 cm, and added to the rib length when calculating the sampling locations. The distal sampling location (4/5 of the total length) of PMO 222.669 landed on a segment containing a healed fracture from when the animal was alive. To maintain consistency with the other rib materials, we substituted it with the proximally neighbouring rib piece. In contrast to the ribs, gastralia were not found articulated or complete, which means that the exact location of the samples in this study is unknown.

For each rib and gastralia sample, one transverse and one longitudinal thin section was produced (Fig. 1A). The rib and gastralia pieces were placed in containers filled with epoxy (EpoFix Resin and EpoFix Hardener), and thin sections were prepared at the Centre de Recherche en Paléontologie, Paris, at the Muséum national d’Histoire naturelle by Vincent Rommevaux, following the methodology outlined by Lamm (2013).

We qualitatively describe the histology of the thin sections, based on examination using a Leica DMPL microscope equipped with a polarizer, analyser, and a lambda (λ) wave plate. Histological images were captured using a Leica MC170 HD microscope camera. Complete thin section images were created by stitching the microscopy pictures using the Image Composite Editor application. The histological terminology used is based on de Buffrénil & Quilhac (2021). Unless clearly stated, observations apply in all sections of the described rib.

**Figure 2:**
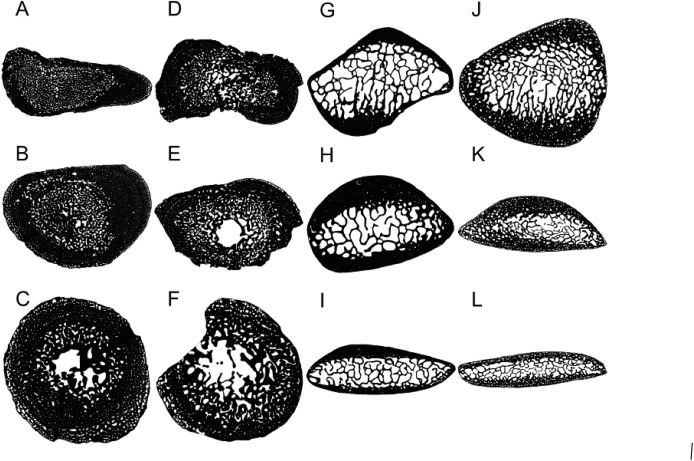
Binarized drawings of transverse rib sections. (A–C) PMO 222.667, †*Keilhauia* sp. (D–F) PMO 222.669, †*Palvennia hoybergeti*. (G–I) NHMO-DMA-42918, *Phocoena phocoena*. (J–L) NHMO-DMA-42918, *Delphinapterus leucas*. (A, D, G, J) Proximal sections. (B, E, H, K) Midshaft sections. (C, F, I, L) Distal sections. Not to scale.

To obtain quantitative microanatomical values, the software BoneProfileR was used, following the methodology outlined by Girondot & Laurin (2003). Before analyses, transverse rib and gastralia cross-sections were manually and digitally converted into binarized black-and-white images (Fig. 2). Importantly, cortical fractures were not classified as voids; instead, they were manually drawn over to be included as part of the bone in the binarized images. For the two ichthyosaurs, the proximal rib sections were digitally reassembled in GIMP (v2.10), to the best extent possible to approximate the original rib morphology prior to sectioning.

Binarized images were analyzed in R v.4.5.2, using BoneProfileR v.4.0, obtaining the following parameters using the protocol described in Girondot & Laurin (2003) and Gônet, Laurin & Girondot (2022): (1) Overall global compactness (hereinafter C_obs_), representing the percentage of the section composed of bone (black pixels). (2) Min, the lower asymptote of the compactness profile, and Max, the upper one, generally reflecting the compactness of the central medullary cavity and of the peripheral cortex, respectively. (3) P–the relative distance from the section’s centre to the point where the most abrupt change in compactness occurs. This number is normalized between 0.00 (centre) and 1.00 (outer boundary). It typically corresponds to the transition zone between the cortex and the medullary cavity (or spongiosa). (4) Slope (S)–the reciprocal of the compactness curve slope at point P, effectively indicating the width of the transition zone between dense cortical bone and the inner medullary cavity. A small S reflects a steep slope (sharp transition), whereas a large S indicates a gradual transition. The R script, the binarized raw files and the BoneProfiler plots can be found in Data S1–S5.

## Results

### Ichthyosaur rib microanatomy and histology

#### PMO 222.667–†Keilhauia sp.

**Figure 3:**
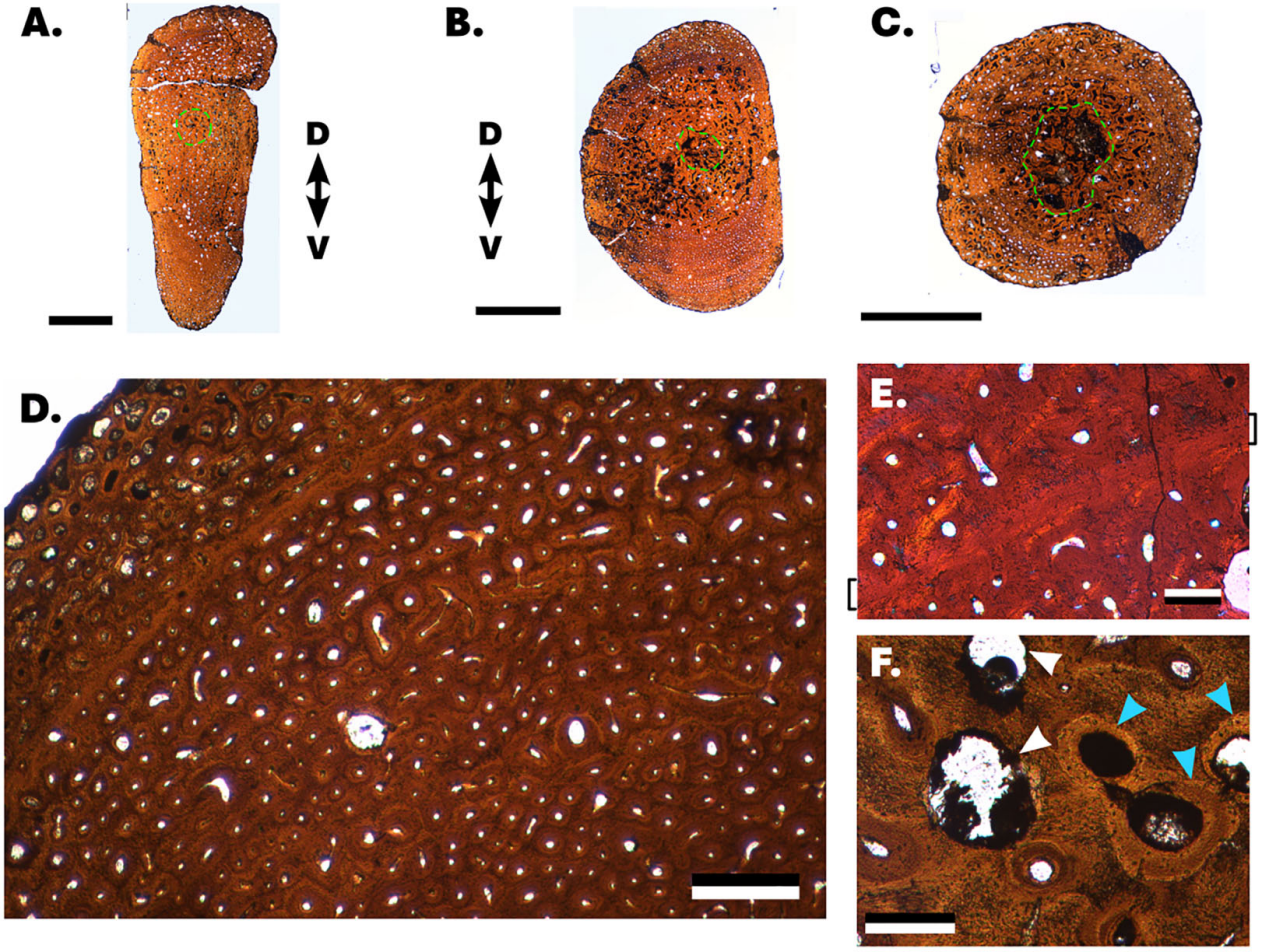
Microstructure of PMO 222.667, †*Keilhauia* sp. (A) Overview of the structure of the proximal cross-section. (B) Overview of the structure of the midhsaft cross-section. (C) Overview of the structure of the distal cross-section. (D) Microanatomy of the primary cortical tissue of the proximal cross-section. (E) Focus on the general organisation of an avascular belt, indicated with black brackets, viewed in XPL with a lambda plate. (F) Example of secondary remodeling; white arrows–erosion bays, blue arrows–secondary osteons. Green lines–Delineation of the medullar territory. Scale bars: A to C–50 mm; D–0.5 mm; E–0.2 mm.

In transverse view, the proximal part of the rib has an elongated pear-shaped outline (Fig. 3A), that changes to oval in the midshaft (Fig. 3B) and further to circular in the distal section (Fig. 3C). Despite some cortical fractures and areas of poor preservation with dark discolorations, the bone is overall well preserved.

The section has a generally cancellous structure composed of thick trabeculae forming a spongiosa without a central lumen, which is surrounded by a denser cortex (Figs. 3A, 3B). The distal section differs from the proximal and midshaft in having a looser trabecular network in the centre of the spongiosa and a more open medullary cavity (Fig. 3C).

The cortex on the ventral side is clearly thicker than the medullary counterpart on the dorsal side in the proximal and midshaft sections (Figs. 3A, 3B), whereas in the distal section, the thickness of the cortex is even throughout all the section (Fig. 3C).

**Figure 4:**
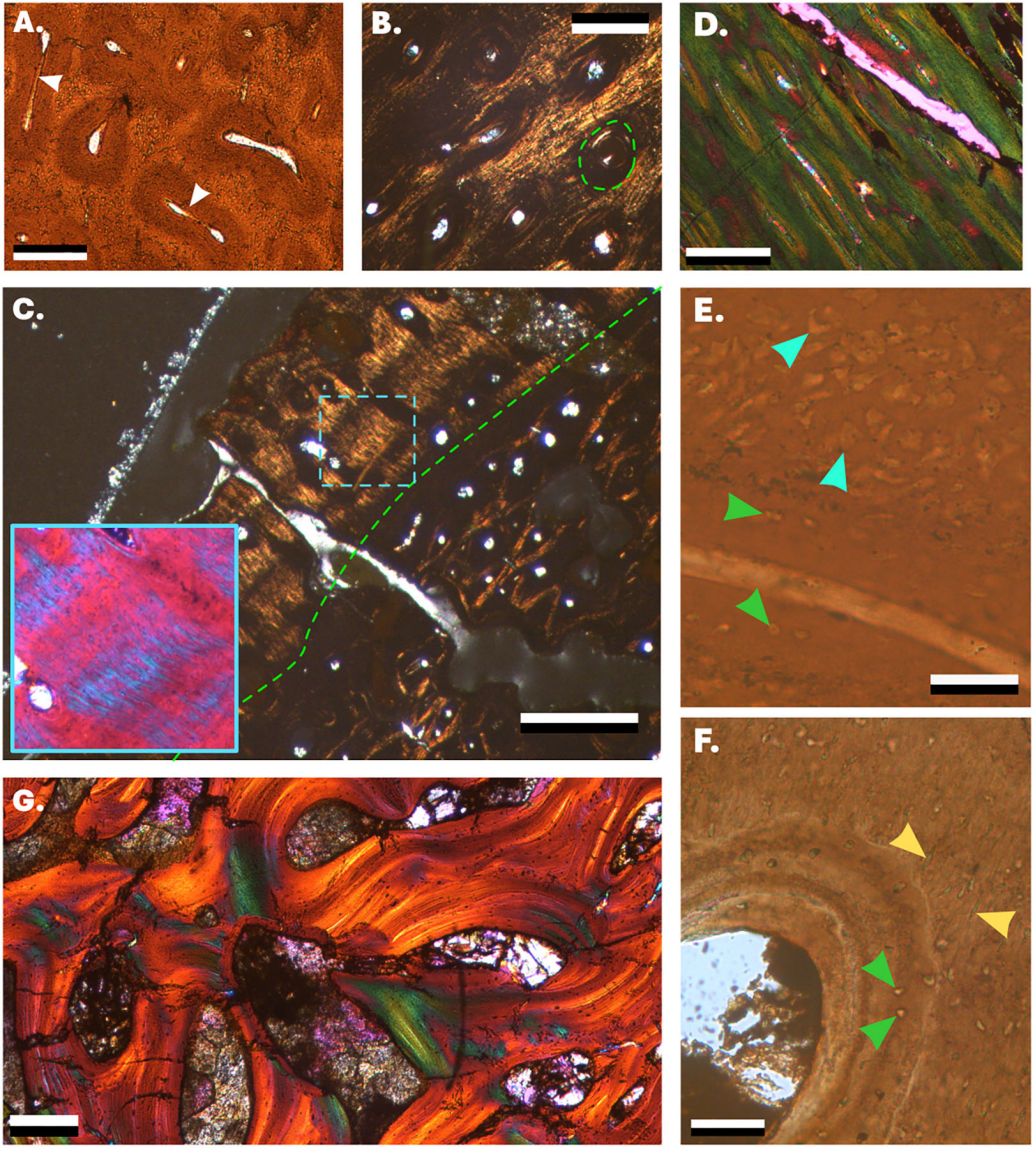
Osteohistology of PMO 222.667, †*Keilhauia* sp. (A) Example of primary osteons displaying connections through Volkmann’s canals (white arrows); (B) Monorefringent primary osteons, embedded in birefringent parallel-fibered matrix, viewed in XPL. (C) Focus on the radial oriented parallel-fibered complex, viewed in XPL. Green line–Delineation of this tissue; Blue dashed square–position of the magnification, displayed in the blue box, which focus on the orientation of the fibers in XPL with a lambda plate; (D) Example of primary cortical tissue in the longitudinal proximal section, displaying both birefringent osteons and surrounding matrix, view in XPL with a lambda plate; (E) Focus on a woven-fibered matrix surrounding an primary osteon, showing clearly globular osteocyte lacunae in deep cortical tissue. Blue arrows–globular osteocyte lacunae; Green arrows–pin-shaped osteocyte lacunae; (F) Examples of pin-shaped and flat-shaped osteocyte lacunae in outer cortical tissue. Green arrows–pin-shaped osteocyte lacunae; Yellow arrows–flat-shaped osteocyte lacunae; (G) Medullary trabeculae. Scale bars: (A, B, G)–0.2 mm; (C, D)–0.5 mm; (E, F)–0.05 mm.

The proximal and midshaft sections have a cortex mainly composed of primary bone with a vascular network predominantly consisting of primary longitudinal osteons (Fig. 3D), whereas the distal section is more variable. The proximal and midshaft sections display some layering, most clearly visible on the dorsal side, with delineated avascular layers (Fig. 3E). However, the bone exhibits secondary remodelling (Fig. 3F) making it difficult to determine whether this layering is concentric or a localized feature. In contrast, there is no concentric layering in the distal section, which is more homogenous. Some osteons are interconnected by a Volkmann’s canal (Fig. 4A). The primary periosteal bone consists of birefringent bone matrix embedding monorefringent osteons (Fig. 4B). The outer cortical regions display more organized birefringent fibres compared to the deeper bone. A particularly distinct area is observed on one half of the dorsal side, where the birefringent fibres form a parallel-fibered matrix oriented perpendicular to the rib margin (Fig. 4C). Although this alignment is localized, it spans a broad portion of the cortex and extends noticeably towards the rib centre, being present in the midshaft section, but missing in the distal one.

On the contrary to transverse cross-sections, the primary cortical tissue in longitudinal sections display bone fibres oriented along the rib’s sagittal axis (Fig. 4D), and embedding primary osteons that all show anisotropic bone fibres oriented along the rib’s sagittal axis (Fig. 4D).

In the innermost part of the cortical region, some bundles of globular osteocyte lacunae can be seen where remodelling did not replace the original primary bone tissue (Fig. 4E). Their expansion is limited to the innermost part of the bone, the rest is mostly composed of flat-shaped osteocyte lacunae associated within the birefringent areas, including both the bone matrix and the osteons (Fig. 4F). These lacunae are aligned with their long axis parallel to the bone fibres. In the transverse cross-section, the lacunae appear pin-shaped.

Avascular layers can be observed in proximal and midshaft parts of the rib. They are characterized by an absence of vascularization, a complete monorefringence, and a lower osteocyte lacunae density (Fig. 3E). One of these layers can be observed throughout the proximal section. Three are seen in the midshaft section, out of which two are localized, whereas the third one, that is closely located to the spongiosa, seems to be concentric. The distal section does not have these layers.

Remodelling is most prominent in the deeper regions of the rib, particularly within the spongiosa (Fig. 4G), but it also occurs in the cortical regions and include resorption bays (Fig. 3F) and secondary osteons (Fig. 3F).

#### PMO 222.669–†Palvennia hoybergeti

**Figure 5:**
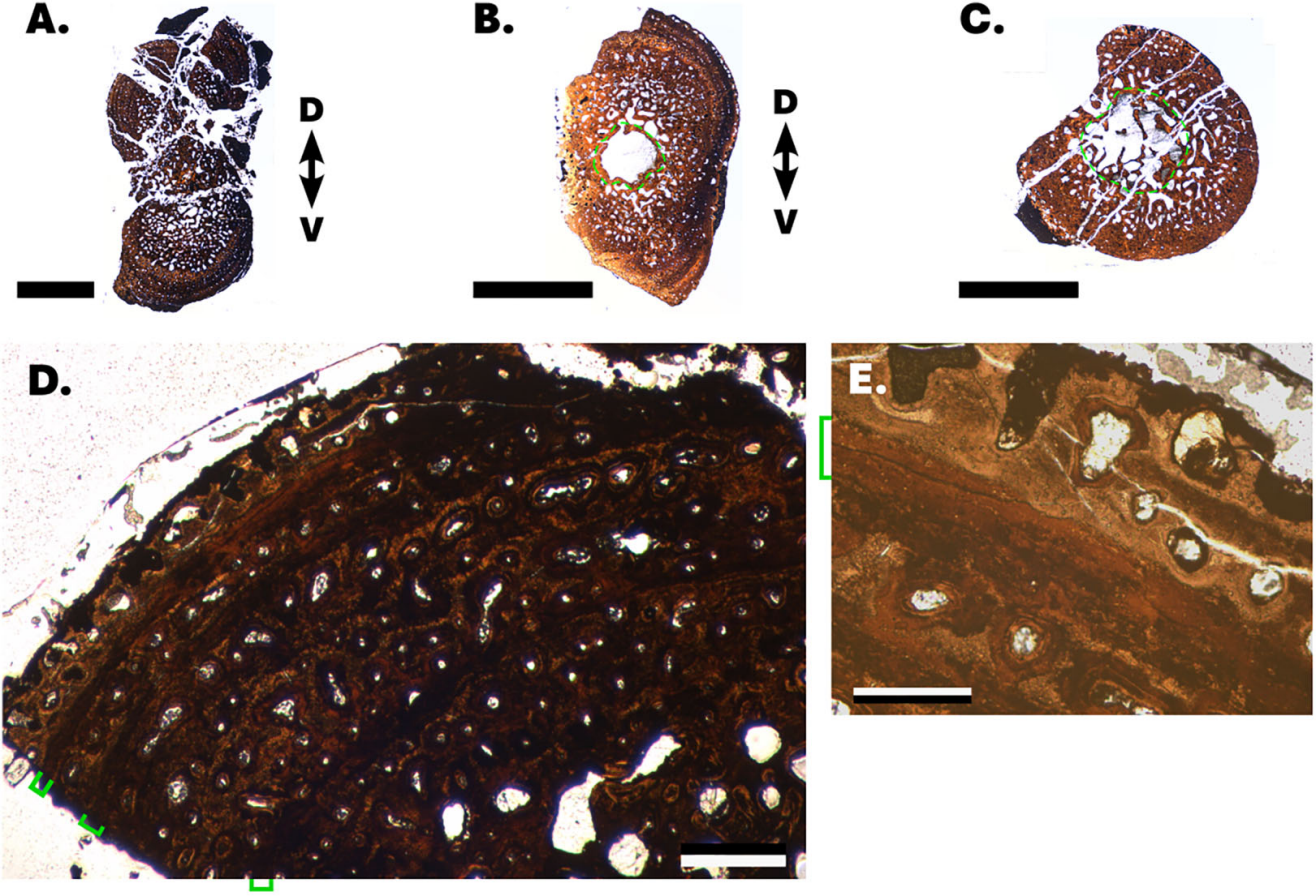
Microstructure of PMO 222.669, †*Palvennia hoybergeti*. (A) Overview of the structure of the proximal cross-section. (B) Overview of the structure of the midshaft cross-section. (C) Overview of the structure of the distal cross-section. (D) Overview of the microanatomy of the primary cortical bone tissue. Green brackets–Avascular layers. (E) Magnification on primary osteons in the primary cortical layer. Green brackets–Avascular layers. Scale bars: (A to C)–50 mm; (D)–0.5 mm; (E)–0.2 mm.

In transverse view, the proximal and midshaft sections have a figure-eight shape (Figs. 5A, 5B), whereas the distal part has an overall circular shape (Fig. 5C). Preservation is poorer compared to the †*Keilhauia* sp. specimen (PMO 222.667), with some contamination and fracturing (Figs. 5A–5C).

Similarly to PMO 222.667, the rib microanatomy can be divided into two main regions: a cancellous inner medullary spongiosa and a denser outer cortex (Fig. 5A). The spongiosa in the proximal section seems to lack a free medullary lumen and forms an irregular trabecular network with intertrabecular spaces that increase in size towards the centre (Fig. 5A). However, in the midshaft and distal sections, a difference is the appearance of a core lumen present inside the spongiosa (Fig. 5B, 5C). Overall, in the distal section, the spongiosa occupies a larger proportion of the section than in the others, and its architecture appears more irregular.

The cortex is thicker dorsally and ventrally (Figs. 5A, 5B), except in the distal section where it is evenly thick (Fig. 5C).

In the proximal and midshaft sections, the outer cortex displays alternating layers of avascular and vascular bone (Figs. 5D, 5E), suggesting possible concentric deposition, though fragmentation limits its confirmation. This pattern is absent in the distal section, which is constituted only of vascularized tissue. Vascularization is predominantly longitudinal and made of primary osteons (Fig. 5D).

**Figure 6:**
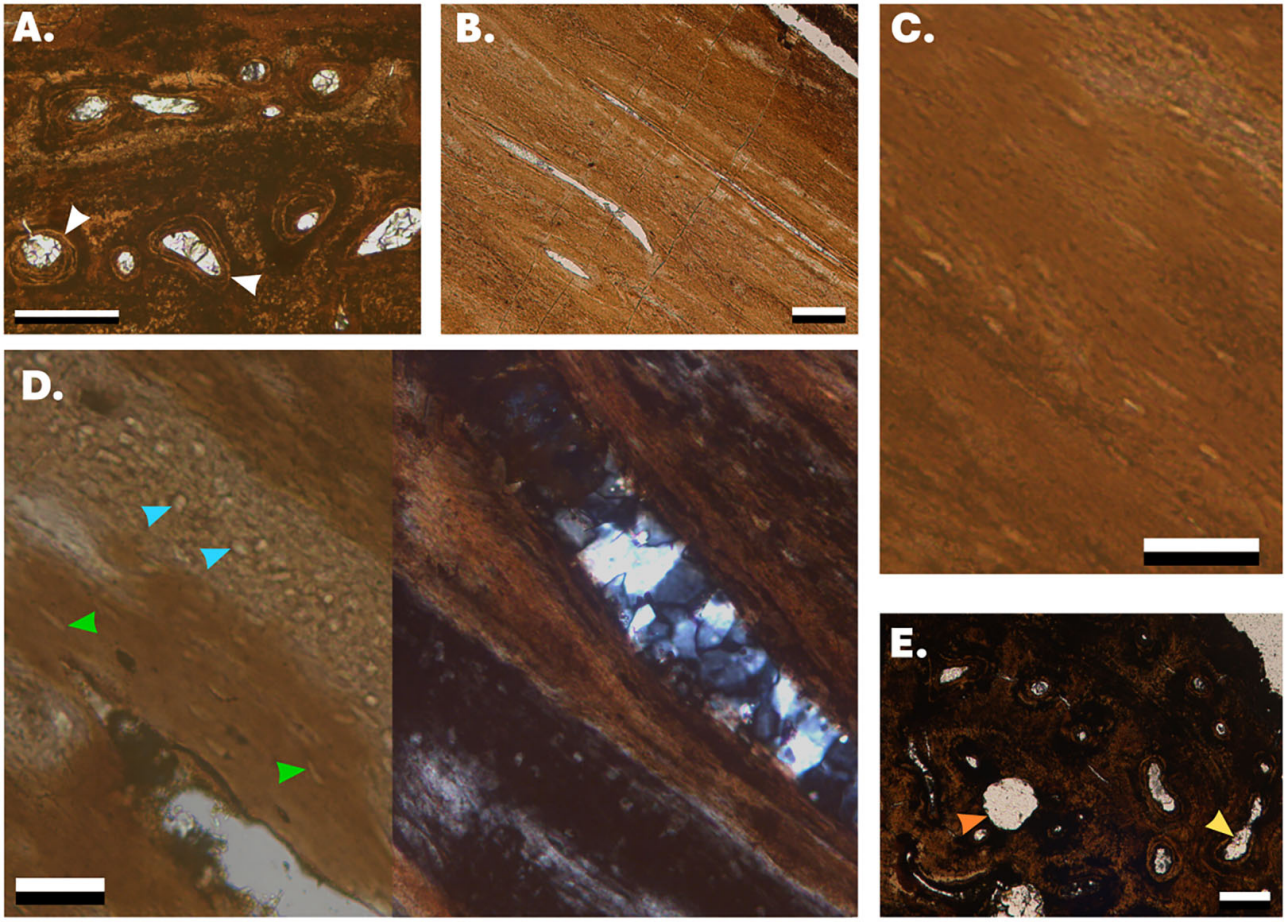
Osteohistology of PMO 222.669, †*Palvennia hoybergeti*. (A) Lines delineating primary osteons, indicated with white arrows. (B) Example of primary osteons viewed in longitudinal section. (C) Focus on flat-shaped osteocyte lacunae. (D) Association of monorefringent tissue with pin-shaped lacunae (blue arrows) and of birefringent tissue with flattened lacunae (green arrows). The left part of the picture is viewed in XPL. (E) The two possible secondary remodeling. Yellow arrow–Secondary osteon; Orange arrow–Erosion bay. Scale bars: (A, B, E)–0.2 mm; (C, D)–0.05 mm.

In the cortex, there is a distinct line separating multiple osteons from the surrounding bone matrix (Fig. 6A), but these lines are missing in the longitudinal sections (Fig. 6B). The osteons are mostly longitudinally oriented, but their direction is less uniform than in the †*Keilhauia* sp. specimen (Fig. 6B). While missing in the proximal section, Volkmann’s canals are present in other parts of the rib.

In the proximal section, the osteocyte lacunae associated with the birefringent areas all are flat-shaped (Fig. 6C). In the midshaft section, osteocyte lacunae associated to birefringent matrix have a flattened shape (Fig. 6D), while monorefringent matrix is associated with both pin-shaped and flat osteocyte lacunae of similar diameter (Fig. 6D).

The preservation obscures the opportunity to fully evaluate possible growth marks. However, in the proximal section, and in parts of the midshaft section, two belts of avascular layers can be observed (Fig. 5D). These layers have the same isotropy and osteocyte lacunae as the surrounding bone and are not fully concentric. The osteons have a general monorefringent appearance in cross-sections, but some show faint concentric lamella.

On the contrary to proximal and midshaft sections, the cortical region of the distal cross-section has been subject to remodelling, with stages of secondary osteon formation being present, in the innermost part of the cortex (Fig. 6E).

#### Interpretation of the microanatomy and histology in the rib sections

Throughout the three sample locations, two main microanatomical regions can be distinguished: the inner medullary region and the outer, more compact, cortical region (Green outline, Figs. 3A–3C). The two regions are distinguished based on the presence of periosteotic deposition indicative of the cortex and endosteal deposition indicative of the medulla. The latter can be identified based on the clear shift from the histological nature of the tissue of the former: on the contrary to the periost, there is no sign of a resorption modelling the scaffolding of the trabeculae; here, the trabeculae seem to be of primary, centripetic, deposition. A third, intermediate, area is located between the other two: the inner cortex, which is distinct from the outer one as being remodelled. However, a clear delineation with the outer cortex, which is not subject to remodelling, is hard to define as remodelling is a gradual process. Therefore, we prefer to consider the entire cortex as one area, especially given that both the inner and outer cortex derive from the same primary process, *i.e.*, periosteotic deposition.

In the two ophthalmosaurid ichthyosaurs, the cortical region is not fully compact, and no classical LAGs are observed in transverse sections. However, the outermost portion of the cortical region in proximal and mid-shaft sections in both specimens show an alternation between vascular and avascular bands. The latter share similar characteristics: they possess a reduced density of osteocyte lacunae. In most sections, most of these avascular bands are not fully concentric. The absence of vascularization is enough to support a decrease, at least local, of the bone growth rate that might even be global, given the extent of some of these layers throughout the ribs. Phases of slower and faster growth is also consistent with the variable presence of woven-parallel matrix. In the distal sections, no alternating bands are present, suggesting a more homogenous growth rate, again emphasizing the importance of known sampling locations.

The avascular bands observed in †*Keilhauia* sp. ribs may indicate changes in the growth rate (Fig. 3E). There is one in the proximal section, and three in the midshaft section. Complicating the interpretation is the lack of full concentricity in two of the three midshaft layers. Therefore, while the observed features share several traits consistent with reduced growth rate—including avascularity and reduced osteocyte lacunae density—their incomplete distribution introduces uncertainty regarding their classification. However, despite not representing annuli as laid down by true cyclical growth in ectothermic vertebrates (*e.g.*, crocodilians), the structures still record changes in growth rate. This might well be localized, as growth rate is not homogeneous throughout a skeletal element.

The presence of primary osteons and localized areas of woven-fibered matrix, demonstrated by the association between globular osteocyte lacunae and isotropic matrix (Faure-Brac, Pelissier & Cubo, 2019), suggest the presence of woven-parallel complex. In other cases, the monorefringence of the tissue can sometimes suggest faster deposition, but a close survey of the lacunae reveals parallel-fibered matrix cut transversally, which is supported by an examination of longitudinal sections (Fig 6D; Faure-Brac, Pelissier & Cubo, 2019). Woven-parallel complex is found in the innermost cortical layer of †*Keilhauia* sp. and indicates rapid bone deposition only in the earliest stage of the growth of this specimen. The possible extent of woven-parallel complex in †*Palvennia hoybergeti*’s rib is unknown, as remodelling and poor preservation removed evidence of earlier deposition, but the predominant presence of parallel-fibered matrix suggests that growth rate decreased. The sections exhibit primary osteons distributed throughout the bone, which are generally embedded in primary bone tissue which partly consists of parallel-fibered, sometimes woven-fibered matrix in †*Keilhauia* sp.

The perpendicular arranged bone fibres observed in the proximal and midshaft sections of †*Keilhauia* sp. (Fig. 4C) are noteworthy as they are difficult to interpret with current understanding of periosteal bone deposition. They resemble tissue coined as “radial-fibered bone” found in a Cretaceous plesiosaur (Pereyra, O’Gorman & Chinsamy, 2025). While suggested by Pereyra, O’Gorman & Chinsamy (2025), we reject the possibility that these structures are Sharpey’s fibres, which are the only currently known organised and radially oriented fibres. In this rib section, the fibres constite a real complex, whereas Sharpey’s fibres tend to be more spread (*e.g.*, Faure-Brac & Cubo, 2023). However, a radially oriented parallel-fibered complex questions the dynamic osteogenesis as we currently understand it. Further research is needed to unveil the ontogenetic process behind its deposition.

### Ichthyosaur gastralia microanatomy and histology

#### PMO 222.667–†Keilhauia sp.

**Figure 7:**
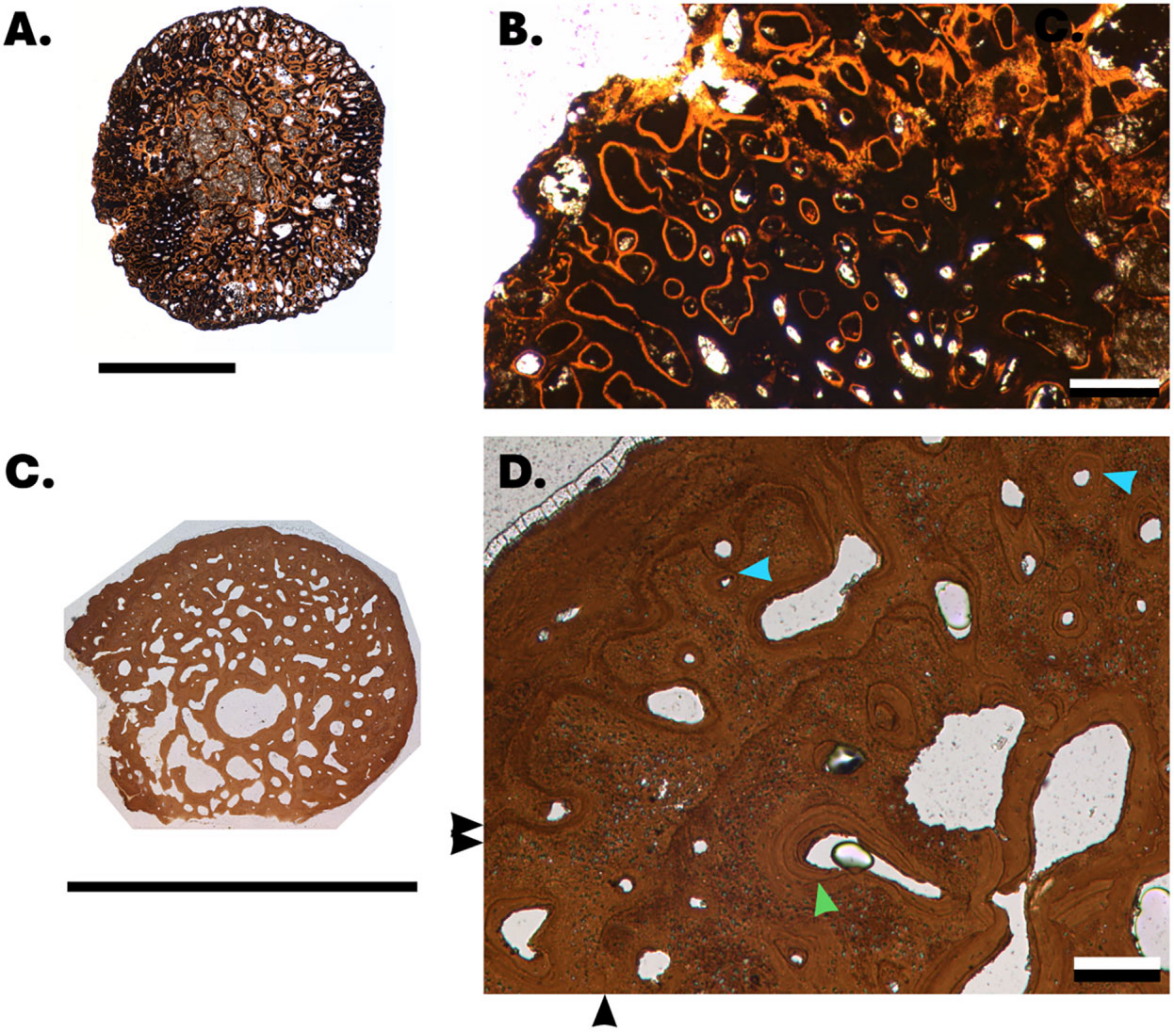
Microanatomy and osteohistology of gastralia from PMO 222.667 and PMO 222.669. (A) Overview of the structure of the cross-section of the gastralia of PMO 222.667. (B) General overview of the microanatomy of the gastralia of PMO 222.667, almost completely darkened, in a cross-section. (C) Overview of the structure of the cross-section of the gastralia of PMO 222.669. (D) Overview of the primary tissue of the gasralia of PMO 222.669, in a cross-section. Black arrows–growth lines; Blue arrows–primary osteons; Green arrow–secondary osteons. Scale bars: (A, C)–50 mm; (B)–0.5 mm; (D)–0.2 mm.

The gastralia has an overall circular cross-section (Fig. 7A). The section is intact but appears almost completely dark when viewed under the microscope, likely due to contamination or diagenetic effect (Fig. 7B). Its microanatomy differs from the rib (Figs. 3A–3C) as the whole section has a spongy appearance with only a very thin outer layer (Fig. 7A). While there is a more cancellous inner region with larger cavities separated by thin trabeculae, the surrounding bone also has a cancellous, irregular appearance, similar to what is observed in the medullary spongiosa of the rib sections (Fig. 7B).

#### PMO 222.669–†Palvennia hoybergeti

The gastralia has a nearly circular cross-section (Fig. 7C). Preservation quality is superior to that of the rib samples, with minimal evidence of contamination. Similar to the gastralia of the †*Keilhauia* sp. specimen (PMO 222.667) (Fig. 7A), this section is more cancellous than the rib cross-sections (Figs. 5A–5C). The spongiosa dominates the internal structure, extending nearly to the bone’s boundary and leaving only a thin outer cortical region. It forms a trabecular network consisting of irregular shaped cavities that become progressively larger toward the deeper regions. Despite its cancellous nature, the vascular network is visible, characterized by longitudinal osteons. The vascularization is composed primarily of primary osteons, as in the rib sections (Fig. 7D). However, several secondary osteons are also present in the outer cortical region. Both primary and secondary periosteal bone exhibits a monorefringent appearance in both transverse and longitudinal sections, which contrasts with the rib sections (Fig. 7D).

In the transverse section, three distinct dark lines are visible in the outer cortical region. These lines appear concentric when traced through the section, although their clarity varies, being sharper in some areas and fainter in others (Fig. 7D).

### Odontocete rib microanatomy and histology

#### NHMO-DMA-42918–Phocoena phocoena

**Figure 8:**
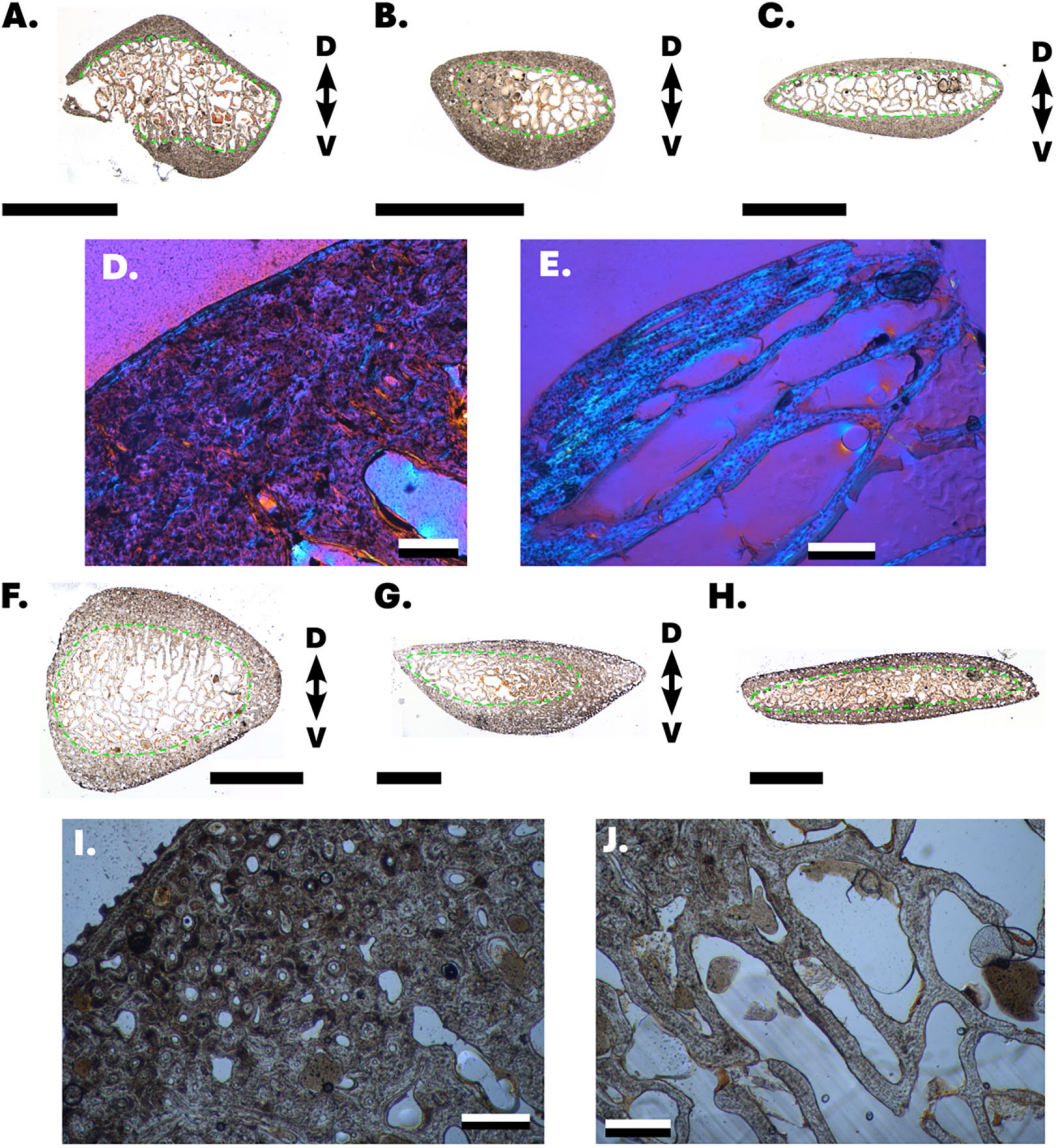
Microanatomy and osteohistology of NHMO-DMA-42918 (*Phocoena phocoena*) and NHMO-DMA-32051 (*Delphinapterus leucas*). (A) Overview of the structure of the proximal cross-section of NHMO-DMA-42918. (B) Overview of the structure of the midshaft cross-section of DMA 42918. (C) Overview of the structure of the distal cross-section of NHMO-DMA-42918. (D) Overview of the microanatomy of the cortical region, viewed in XPL with a lambda plate. (E) Overview of the longitudinal microanatomy, viewed in XPL with a lambda plate. (F) Overview of the structure of the proximal cross-section of NHMO-DMA-32051. (G) Overview of the structure of the midshaft cross-section of NHMO-DMA 32051. (H) Overview of the structure of the distal cross-section of NHMO-DMA 32051. (I) Overview of the microanatomy of the cortical region; viewed in XPL with a lambda plate. (J) Overview of the longitudinal microanatomy, viewed in XPL with a lambda plate. Green lines: delineation of the endochondral territory. Scale bars: (A, B, C, F, G, H)–50 mm; (E, I, J)–0.5 mm; (D)–0.2 mm.

The proximal rib has a parallelogram-like cross-section with rounded edges (Fig. 8A), whereas the midshaft has an oval outline (Fig. 8B), and the distal section is more elliptical (Fig. 8C). Cortical thickness is consistently thicker dorsally than ventrally (Figs. 8A–8C). Throughout, the rib has two distinct microanatomical regions: an inner medullary spongiosa without a free lumen and an outer, compact cortex (Green outline, Figs. 8A–8C). The medullary region has a network of thin trabeculae forming irregular, mesh-like cavities. In the proximal and midshaft longitudinal sections, the spongiosa consists of long trabeculae aligned along the sagittal axis of the rib piece. A few trabeculae connect these, forming elongated cavities. In the distal longitudinal section, the spongiosa almost occupies the whole section, with the cortex reduced to the same thickness as the trabeculae. The trabecular network differs from the other two locations in having central trabeculae forming a more mesh-like appearance with no preferred orientation.

The cortex exhibits a longitudinal vascular network constituted mainly of osteons, with the diameter of canals being smaller than in the ichthyosaur specimens. No concentric or localized layering is observed. In all the three sections (proximal, midshaft and distal), extensive remodelling of the cortical region has resulted in the formation of dense Haversian bone and loss of most histological features (Fig. 8D). The bone is fully remodelled by secondary osteons that all appear fully developed. Located at the outer edge of the rib, a parallel-fibered bone matrix that appears birefringent in cross-polarized light circumvent the section (Fig. 8E). Apart from it, no remnants of primary periosteal bone are visible in any part of the cortical bone.

#### NHMO-DMA-32051–Delphinapterus leucas

The transverse section of the proximal rib has a triangular shape with rounded edges (Fig. 8F), that turns to an oval shape in the midshaft cross-section (Fig. 8G), and a more elliptical one in the distal cross-section (Fig 8H). Two distinct regions can be distinguished: an inner, more cancellous medullary spongiosa, and a surrounding cortical layer (Green outline, Figs. 8F–8H). No free inner lumen is present. The medullary spongiosa is filled with thin interconnected trabeculae, though these are unevenly distributed, resulting in cavities of varying sizes.

In comparison to the *Phocoena phocoena* specimen, which displays a denser and more compact cortex, this specimen shows a relatively more cancellous and porous cortical region. The cortical thickness is uneven in proximal and midshaft, and evenly thick in the distal section. The cortex contains a vascular network of longitudinal osteons, but no evidence of concentric layering is present.

The medullary spongiosa occupies most of the longitudinal section, with long trabeculae aligned parallel to the rib’s sagittal axis (Fig. 8I), creating a similar pattern to the *Phocoena phocoena* specimen (Fig. 8E).

As in NHMO-DMA-42918, the cortical region throughout the rib shows extensive remodelling through the formation of secondary osteons, resulting in dense Haversian bone (Fig. 8J). However, some differences to the *Phocoena phocoena* specimen are evident. Notably, the *Delphinapterus leucas* rib contains secondary osteons at various stages of development (Fig. 8J), including resorption bays, forming osteons, and fully developed osteons, giving the cortical region a more cancellous appearance than in the porpoise. No remnants of primary bone are present, and there is no outer parallel-fibered layer.

#### Interpretation of histology and microanatomy of cetaceans

Both cetacean specimens exhibit dense Haversian bone tissue throughout the rib, a result of extensive bone remodelling that has completely replaced the primary periosteal bone, preventing the identification of any remaining primary bone tissue. Although both taxa display Haversian remodelling, its extent differs significantly, and other differences can also be noted. In *Phocoena phocoena*, the outer cortical region displays the formation of an external fundamental system (Fig. 8E), a feature typically indicative of somatic maturity (de Buffrénil & Quilhac, 2021) suggesting that this specimen had reached skeletal maturity at the time of death. This finding also likely accounts for the differences observed in cortical compactness between the two species. *Delphinapterus leucas* exhibits a more cancellous cortical structure, caused by ongoing bone remodelling, whereas in *Phocoena phocoena*, remodelling appears to have slowed or ceased.

### Microanatomical parameters

The ichthyosaur and cetacean rib sections display interesting differences in terms of microanatomical compactness and inner architecture. Ichthyosaur rib sections have a higher observed compactness than cetacean sections (Table 1), which is evident from the more open medullary cavity of the latter. Moreover, while P is lower in ichthyosaurs than in cetaceans, the opposite is true for S. Therefore, these parameters indicate (1) more compact sections in ichthyosaurs with (2) a smaller open cavity and (3) a less defined territorialization of the medulla and of the cortex, echoing the histological description above. P and S vary along the sections but without any defined pattern in ichthyosaurs. In cetaceans, S is relatively constant, whereas P seems to drop slightly at the middle of the rib.

**Table 1:** Microanatomical values obtained *via* BoneProfileR.

Taxon	Specimen	Section	C_obs_	P	S	Min	Max
†*Keilhauia* sp.	PMO 222.667	Pro.	0.887	0.583	0.228	0.799	0.928
†*Keilhauia* sp.	PMO 222.667	Mid.	0.887	0.416	0.405	0.754	0.929
†*Keilhauia* sp.	PMO 222.667	Dis.	0.861	0.294	0.056	0.456	0.910
†*Keilhauia* sp.	PMO 222.667	Gastralia	0.58	0.498	0.266	0.447	0.744
†*Palvennia hoybergeti*	PMO 222.669	Pro.	0.876	0.476	0.184	0.658	0.956
†*Palvennia hoybergeti*	PMO 222.669	Mid.	0.801	0.241	0.244	0.000	0.934
†*Palvennia hoybergeti*	PMO 222.669	Dis.	0.750	0.350	0.120	0.173	0.921
†*Palvennia hoybergeti*	PMO 222.669	Gastralia	0.82	0.339	0.598	0.184	0.957
*Phocoena phocoena*	NHMO-DMA-42918	Pro.	0.526	0.645	0.063	0.314	0.969
*Phocoena phocoena*	NHMO-DMA-42918	Mid.	0.691	0.598	0.101	0.232	0.998
*Phocoena phocoena*	NHMO-DMA-42918	Dis.	0.585	0.742	0.116	0.309	0.920
*Delphinapterus leucas*	NHMO-DMA-32051	Pro.	0.629	0.628	0.122	0.232	0.864
*Delphinapterus leucas*	NHMO-DMA-32051	Mid.	0.658	0.416	0.185	0.102	0.821
*Delphinapterus leucas*	NHMO-DMA-32051	Dis.	0.578	0.610	0.101	0.224	0.839

**Note:**

Pro, proximal; Mid, midshaft; Dis, distal; C_obs,_ observed compactness; P, Inflexion point; S, Slope.

For the ichthyosaur gastralia, values resemble those of the ribs, especially when compared to the midshaft and distal sections, with the exception of the C_obs_ of PMO 222.667, which is lower than in the ribs (Table 1).

## Discussion

Ribs are not uniform structures, shown clearly by sampling at three different sites as we did in this study. Examination of the cross-sections reveals not only interspecific differences but also notable morphological changes along the rib shaft, including the shape of the cross-section. In †*Keilhauia* sp., the cross-section changes from a pyriform profile proximally to a circular shape distally (Figs. 3A–3C). Similarly, †*Palvennia hoybergeti* has a figure-eight shape proximally that becomes circular towards the distal end (Figs. 5A–5C). Previous work on ophthalmosaurid ichthyosaurs demonstrated significant variation in ribs beyond the typical figure eight-cross-section used for instance *e.g.*, in phylogenetic analysis (Delsett et al., 2017, 2018, 2019). Our study adds to this conclusion, implying caution in use of such characters unless sampling location is known.

The odontocetes also show within-element variation, but in contrast to the ichthyosaurs, *Phocoena phocoena* (Figs. 8A–8C) and *Delphinapterus leucas* (Figs. 8F–8H) display a shift from a more tubular structure proximally to a flatter diploe-type structure distally. In addition to shape morphology, histological and microanatomical data demonstrate that sampling location present a possible bias, as these features vary between proximal, midshaft and distal sections, as in other amniote groups with systematic sampling along the rib shaft (Gray et al., 2007; Houssaye et al., 2015; Klein, Canoville & Houssaye, 2019; Waskow & Sander, 2014).

### Rapid and ongoing growth in ichthyosaur specimens

Features such as bone matrix type, osteocyte morphology, and vascularization provide key insights into the rate and pattern of bone formation (de Buffrénil & Quilhac, 2021; Marotti, 2010). A commonly cited tissue type in ichthyosaur osteohistology is the woven-parallel complex (Anderson et al., 2018; Delsett et al., 2025; Houssaye, Nakajima & Sander, 2018; Houssaye et al., 2014; Kolb, Sanchez-Villagra & Scheyer, 2011; Nakajima, Houssaye & Endo, 2014), which is also found in the ribs in the Middle Jurassic ophthalmosaurid †*Mollesaurus* (Talevi & Fernández, 2012). It consists of primary periosteal bone with a woven-fibered matrix, globular-shaped osteocytes, and primary osteons, and is typically associated with rapid bone deposition and, by extension, high growth rates (de Buffrénil & Quilhac, 2021; Marotti, 2010). While woven-parallel complex was indeed found in †*Keilhauia*, it cannot be confirmed in †*Palvennia hoybergeti*. Moreover, for both species, the periost is dominated by parallel-fibered complex. Given its high vascularization, it supports a bone growth rate intermediate between woven-parallel complex and avascular parallel-fibered complex. We can then infer a first burst of growth, as shown by the woven parallel complex, before a second slower phase with the vascularized parallel-fibered complex (de Buffrénil & Quilhac, 2021; Marotti, 2010). Interestingly, the rate of vascularization in both ophthalmosaurid specimens studied here does not seem to decrease (Figs. 3D, 5D), suggesting a steady growth.

The gastralia of †*Palvennia hoybergeti* presents a markedly different histological structure. Here, longitudinal primary osteons are embedded in a woven-fibered matrix associated with globular osteocyte lacunae (Fig. 7D). These features meet all diagnostic criteria for a woven-parallel complex, which is thus present more consistently in the gastralia than in the ribs of the same specimen. This disparity between the rib and gastralia suggests that the variation in histology reflects element-specific growth dynamics and mosaic development within the skeleton. The widespread presence of woven-parallel complexes in various taxa and skeletal elements of other ichthyosaurs, along with growing evidence supporting endothermy-like metabolism (Bernard et al., 2010; Delsett et al., 2022; Lindgren et al., 2018; Séon et al., 2025), support this interpretation.

### Growth marks

Growth marks refer to interruptions in skeletal growth, visible in the primary periosteal cortex of bone. They are often broadly categorized into two types: lines of arrested growth (LAGs), characterized by distinct, dark concentric lines representing temporary halts in bone deposition, and annuli, any cyclical changes in the bone matrix structure (de Buffrénil & Quilhac, 2021). The formation of both LAGs and annuli has historically been associated with annual or biannual reductions in growth rate, allowing these features to be used in skeletochronology to estimate the age and growth patterns of extinct animals, but recent studies have challenged this assumption, as the relation between different growth marks and the physiological processes they record are complex and not fully understood (Chinsamy & Pereyra, 2025; Pereyra & Chinsamy, 2026). Observations of growth marks in ichthyosaurs remain limited, but has been observed in Triassic †*Mixosaurus*, †*Grippia* and †*Cymbospondylus* (Delsett et al., 2025; Kolb, Sanchez-Villagra & Scheyer, 2011), and possibly in Jurassic †*Stenopterygius* (Anderson et al., 2018; Wallstedt et al., 2024), and cyclical growth is likely a common feature within †Ichthyopterygia (Houssaye et al., 2014).

In the ribs of †*Keilhauia* in this study, structures that record changes in growth rate were observed (Fig. 3E). These are not fully concentric and thus not true cyclical growth marks, but are interpreted as records of localized growth variation.

The gastralia of †*Palvennia hoybergeti* preserves growth marks, not in the form of annuli, but rather as LAGs (Fig. 7D). These LAGs are visible as distinct dark contour lines that can be readily identified under lower magnifications. They are located within the primary bone of the gastralia and can generally be traced in a concentric pattern across the section, although some regions show fainter contours or even apparent absences. Although the concentric nature of these LAGs may be open for debate, these features share similarities with observations in the gastralia of †*Mixosaurus* (Kolb, Sanchez-Villagra & Scheyer, 2011). While Anderson et al. (2018) did not find LAGs in the gastralia of †*Stenopterygius*, the observations from †*Palvennia hoybergeti* suggest that LAGs are not exclusive to †*Mixosaurus*, but also occur in later ichthyosaurs. This opens the possibility that gastralia could be a promising skeletal element for identifying reliable growth marks, and future studies could benefit from systematically sampling gastralia across multiple taxa.

### Rib compactness

Although histological comparisons between the ichthyosaur and cetacean specimens remain limited due to remodelling in the cetacean ribs, meaningful microanatomical comparisons can be made, as the presence of primary bone does not impact microanatomical assessments. The quantitative microanatomical data (Table 1) shows larger observed compactness in all transverse sections in the ichthyosaurs compared to the toothed whales. This suggests that, relative to rib size, the two ichthyosaur ribs were denser and likely heavier than the whale ribs. The lowest compactness value among the ichthyosaur samples was the distal section of †*Palvennia hoybergeti* (Fig. 2F), likely due to the more cancellous structure in the medullary spongiosa, but even this section is more compact than any of the cetacean sections. The increased density in the ichthyosaur material is further supported by elevated Min values, with the exception of the midshaft and distal section of †*Palvennia hoybergeti*, where a central core lumen is present (Figs. 2E–2F).

The Max compactness values are relatively consistent across all specimens. The highest observed value is seen in the midshaft section (Fig. 2H) of *Phocoena phocoena*, while the lowest is observed in the midshaft section (Fig. 2K) of *Delphinapterus leucas*. These differences are caused by the variation in cortical vascularization: *Phocoena phocoena* only exhibits complete secondary osteons in the cortex (Fig. 8D), while *Delphinapterus leucas* has numerous resorption bays (Fig. 8I), which reduces cortical compactness.

The *P* value (indicating the position of the largest transition in compactness) does not follow a consistent pattern across the specimens, likely because of the variation in the medullary spongiosa. It might also result from limited sample size. In some cases, such as the midshaft of †*Palvennia hoybergeti*, the *P* value likely marks the boundary between the inner lumen and the perimedullar region (Fig. 2E), rather than the transition to the cortex. Cetaceans generally have lower S values, suggesting a sharper and more abrupt transition between spongiosa and cortical bone. Interestingly, a similar steep transition is also seen in the distal section of †*Keilhauia*, which could indicate a central lumen starting to emerge (Fig. 2C).

A distinct pattern is observed when comparing proximal and distal sections of the same ichthyosaur rib. The proximal and midshaft sections consistently exhibit higher compactness and higher minimum values than the distal sections (with the exception of the midshaft of †*Palvennia hoybergeti*). This pattern aligns with findings from three previous ichthyosaur rib studies that analyzed different locations along the rib (Anderson et al., 2018; Nakajima, Houssaye & Endo, 2014; Wallstedt et al., 2024). Like our results, these studies show that distal rib sections are more cancellous compared to the proximal regions, albeit based on visible differences, not quantitative values. Varying microanatomy at different sampling locations is also evident in studies on fossil whale ribs, likely due to a combination of the growth direction and biomechanical needs (Gray et al., 2007; Houssaye et al., 2015).

The high compactness observed in the ribs of both †*Keilhauia* and †*Palvennia* approaches the “osteosclerotic” condition observed in many aquatic tetrapods (Houssaye, 2009; Klein, Canoville & Houssaye, 2019). This finding appears to contradict the general pattern of cancellous bone typically reported in dolphin-like ichthyosaurs from the Jurassic and Cretaceous (*e.g.*, Houssaye, Nakajima & Sander, 2018; Houssaye et al., 2014), but is not an isolated observation (Houssaye, Martin Sander & Klein, 2016). Talevi & Fernández (2012) described a rib of †*Mollesaurus* exhibiting unexpectedly high compactness, and since then, several studies on Jurassic ichthyosaurs have reported similar dense ribs (Anderson et al., 2018; Massare et al., 2014; Wallstedt et al., 2024). Earlier publications, such as Kiprijanoff (1881) and Seitz (1907), also show ribs with comparable compact morphology. de Ricqlès, Taquet & de Buffrénil (2009) described one rib thin section (likely Jurassic, from the UK) with an “osteosclerotic” condition, and one (of unknown age, from Spitsbergen) that is relatively cancellous. The addition of †*Keilhauia* and †*Palvennia* reinforces the view that relatively dense and compact rib bone is a recurring trait among late, thunniform ichthyosaurs.

In both †*Keilhauia* and †*Palvennia*, the gastralia differ from the ribs in being cancellous throughout, without a compacted cortical region. Bearing in mind the unknown sample location along the gastralia, the low compactness also differs from a previous study which found relatively compact gastralia in †*Mixosaurus* (Kolb, Sanchez-Villagra & Scheyer, 2011), something that is also the case in the few other studied marine reptiles (Klein, Canoville & Houssaye, 2019).

The high degree of compactness observed in the ribs is therefore unlikely to be representative of the entire skeleton and is instead probably an adaptive specialization of the rib cage. This increased compactness, and by extension, weight, of the ribs may serve as ballast, contributing to buoyancy control by countering the updrift produced by air-filled lungs. It would also mean less cavities for lipids that likely filled voids in the skeleton, also with implications for buoyancy. Such a strategy could be particularly important for ichthyosaurs, whose morphology and swimming mechanics might have differed from those of modern cetaceans (Motani, 2002a, 2002b). Unlike ichthyosaurs, cetaceans possess a horizontally oriented caudal fluke and a vertebral column that facilitates an efficient up-and-down motion which possibly aids in the initiation phase of diving behaviour. In contrast, ichthyosaurs, which had vertically oriented tail flukes and a side-to-side mode of swimming, may have benefited from increased weight in the anterior dorsal region to assist in overcoming positive buoyancy during initial phase of a dive (Fish, 2023; Fish & Lauder, 2017). Thus, the denser rib structure in ichthyosaurs may represent a functional adaptation linked to their specific locomotor style and buoyancy management needs.

## Conclusion

In this study, ophthalmosaurid ichthyosaur and modern toothed whale ribs were sampled in order to detect possible convergent microstructural patterns, and to investigate ichthyosaur growth. Importantly, sample locations were at three set locations along the rib, capturing variation throughout the element.

The histology of ophthalmosaurid ribs in this study include areas with woven parallel complex, in line with previous studies, and adding to other indications of rapid growth and possible endothermy. Investigating growth patterns is often challenging due to cancellous ichthyosaur skeletal elements. However, the cortical region in Late Jurassic ophthalmosaurid ichthyosaur ribs targeted here, is relatively compact and shows bands representing slower and faster growth, and the gastralia are likely more promising for skeletochronology, as they might preserve true LAGs.

The ichthyosaurs possess relatively compact ribs compared to modern toothed whales, which might be an adaptation for ballasting the body close to the lungs.

Rib microstructure in the beluga and harbour porpoise is also described for the first time. Their histology reflects widespread remodelling, and while this provides limited opportunities for comparison to the ichthyosaur specimens, it offers valuable direction for future research. To detect convergences, studies should focus on juvenile specimens that retain primary bone tissue, or on extinct cetacean material that, as seen in de Buffrénil & Mazin (1990), does not display the same dense Haversian bone type. However, as demonstrated in the ichthyosaur material, there is skeletal-specific variation within and across species. Consequently, future comparative histological studies with ichthyosaurs should consider a wider range of extant and extinct cetacean taxa to more accurately assess patterns of convergence.

## Supplemental Information

10.7717/peerj.21486/supp-1 Supplemental Information 1 R script for histomorphometric values using BoneProfileR.

10.7717/peerj.21486/supp-2 Supplemental Information 2 Microanatomical plots for all transverse rib sections. Output plots from BoneProfileR for all rib thin sections. A PMO 222.667 *Keilhauia* sp. Proximal B PMO 222.667 *Keilhauia* sp. Mid-shaft C PMO 222.667 *Keilhauia* sp. Distal D PMO 222.669 *Palvennia hoybergeti* Proximal E PMO 222.669 *Palvennia hoybergeti* Mid-shaft F PMO 222.669 *Palvennia hoybergeti* Distal G NHMO-DMA 42918 *Phocoena phocoena* Proximal H NHMO-DMA 42918 *Phocoena phocoena* Mid-shaft I NHMO-DMA 42918 *Phocoena phocoena* Distal J NHMO-DMA 32051 *Delphinapterus leucas* Proximal K NHMO-DMA 32051 *Delphinapterus leucas* Mid-shaft L NHMO-DMA 32051 *Delphinapterus leucas* Distal

10.7717/peerj.21486/supp-3 Supplemental Information 3 Binarized rib cross sections used for BoneProfileR. Raw data for BoneProfiler including binarized photos of all 12 rib sections (PMO 222.667, PMO 222.669, NHMO-DMA-42918, NHMO-DMA-32051).

10.7717/peerj.21486/supp-4 Supplemental Information 4 Binarized gastralia sections.

10.7717/peerj.21486/supp-5 Supplemental Information 5 Microanatomical plots for gastralia sections.

## Additional Information and Declarations

### Field Study Permissions

The following information was supplied relating to field study approvals (*i.e*., approving body and any reference numbers):

Governor of Svalbard.

### Data Availability

The following information was supplied regarding data availability:

The data and code are available in the Supplemental Files.
