# Supplementary material for: Rib microstructure in thunniform ichthyosaurs and toothed whales
Source: PeerJ. 2026 Jul 7;14:e21486. doi: 10.7717/peerj.21486 (PMC13353231; doi:10.7717/peerj.21486)
Supplement: Supplemental Information 2 — Output plots from BoneProfileR for all rib thin sections. A PMO 222.667 Keilhauia sp. Proximal B PMO 222.667 Keilhauia sp. Mid-shaft C PMO 222.667 Keilhauia sp. Distal D PMO 222.669 Palvennia hoybergeti Proximal E PMO 222.669 Palvennia hoybergeti Mid-shaft F PMO 222.669 Palvennia hoybergeti Distal G NHMO-DMA 42918 Phocoena phocoena Proximal H NHMO-DMA 42918 Phocoena phocoena Mid-shaft I NHMO-DMA 42918 Phocoena phocoena Distal J NHMO-DMA 32051 Delphinapterus leucas Proximal K NHMO-DMA 32051 Delphinapterus leucas Mid-shaft L NHMO-DMA 32051 Delphinapterus leucas Distal [file peerj-14-21486-s002.pdf]

A PMO 222.667
Keilhauia sp. Proximal

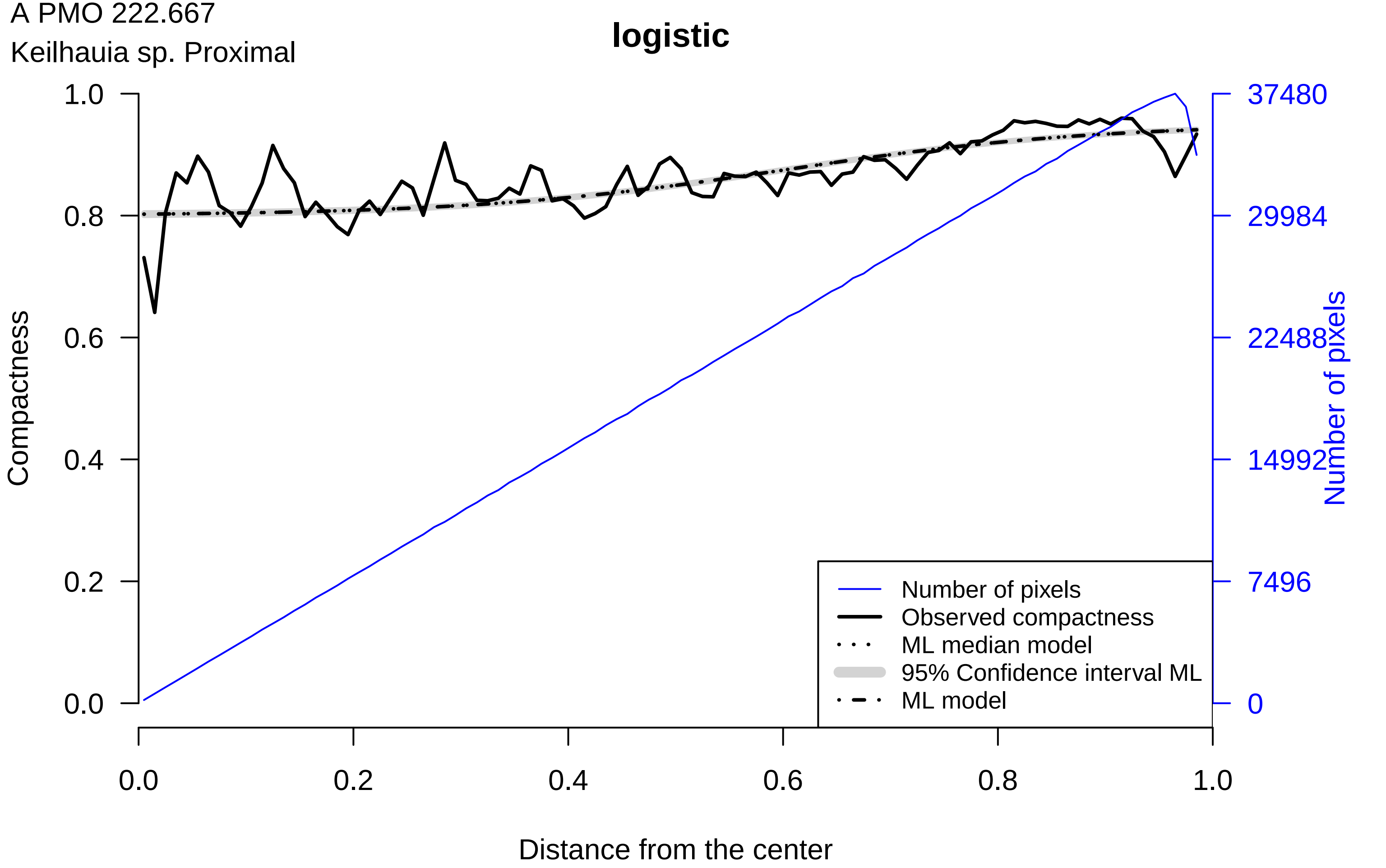

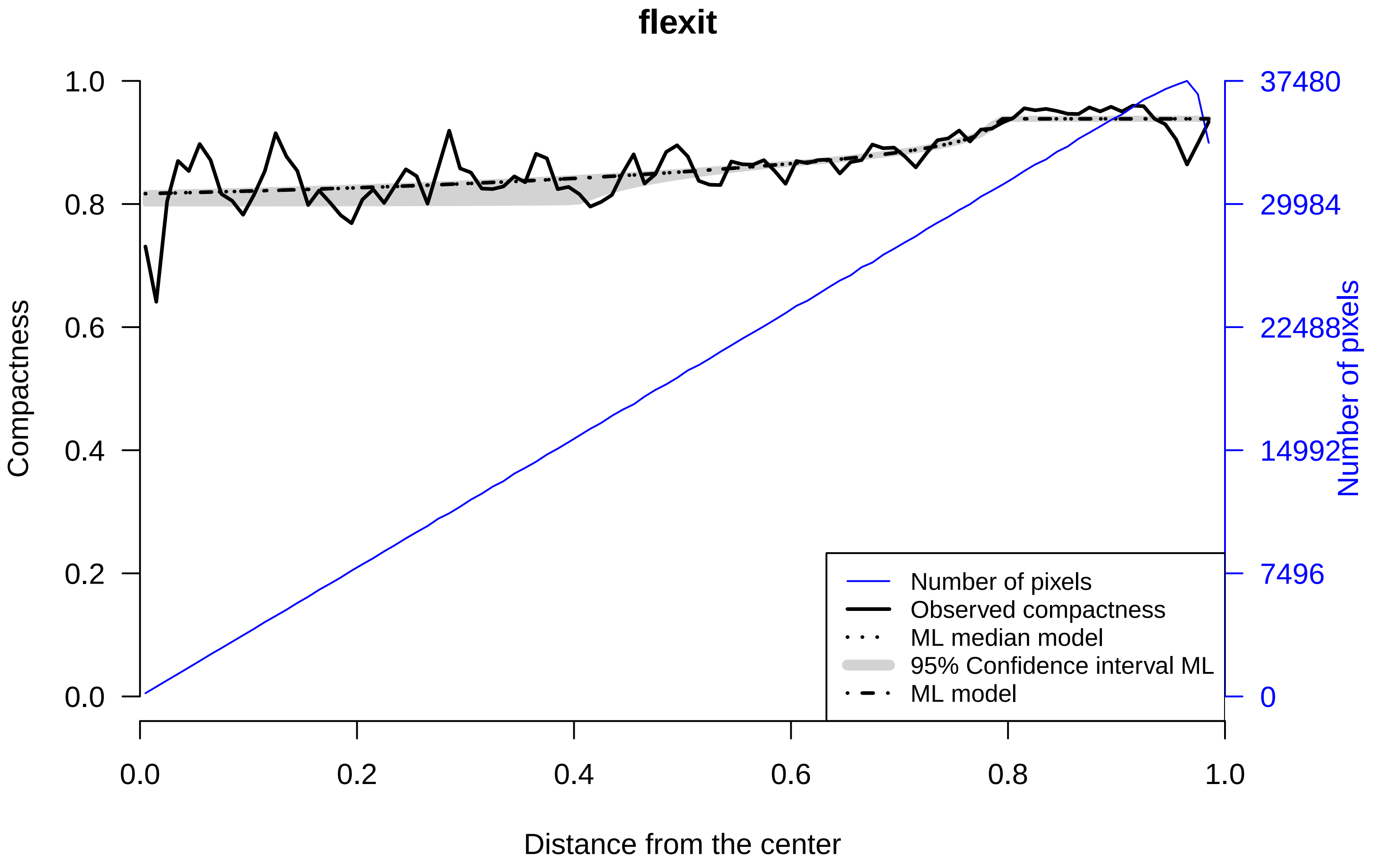

B PMO 222.667
Keilhauia sp. Mid-shaft

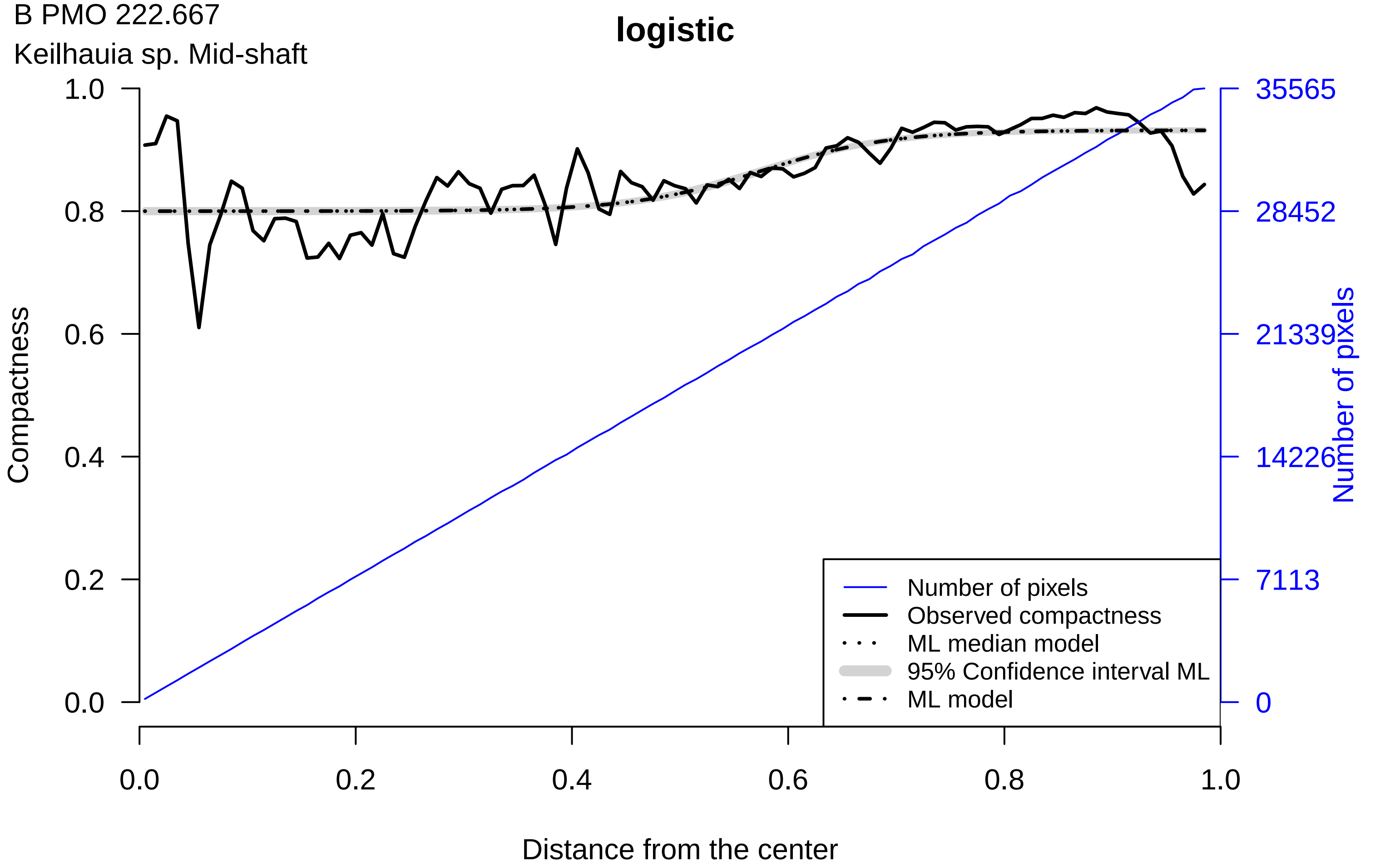

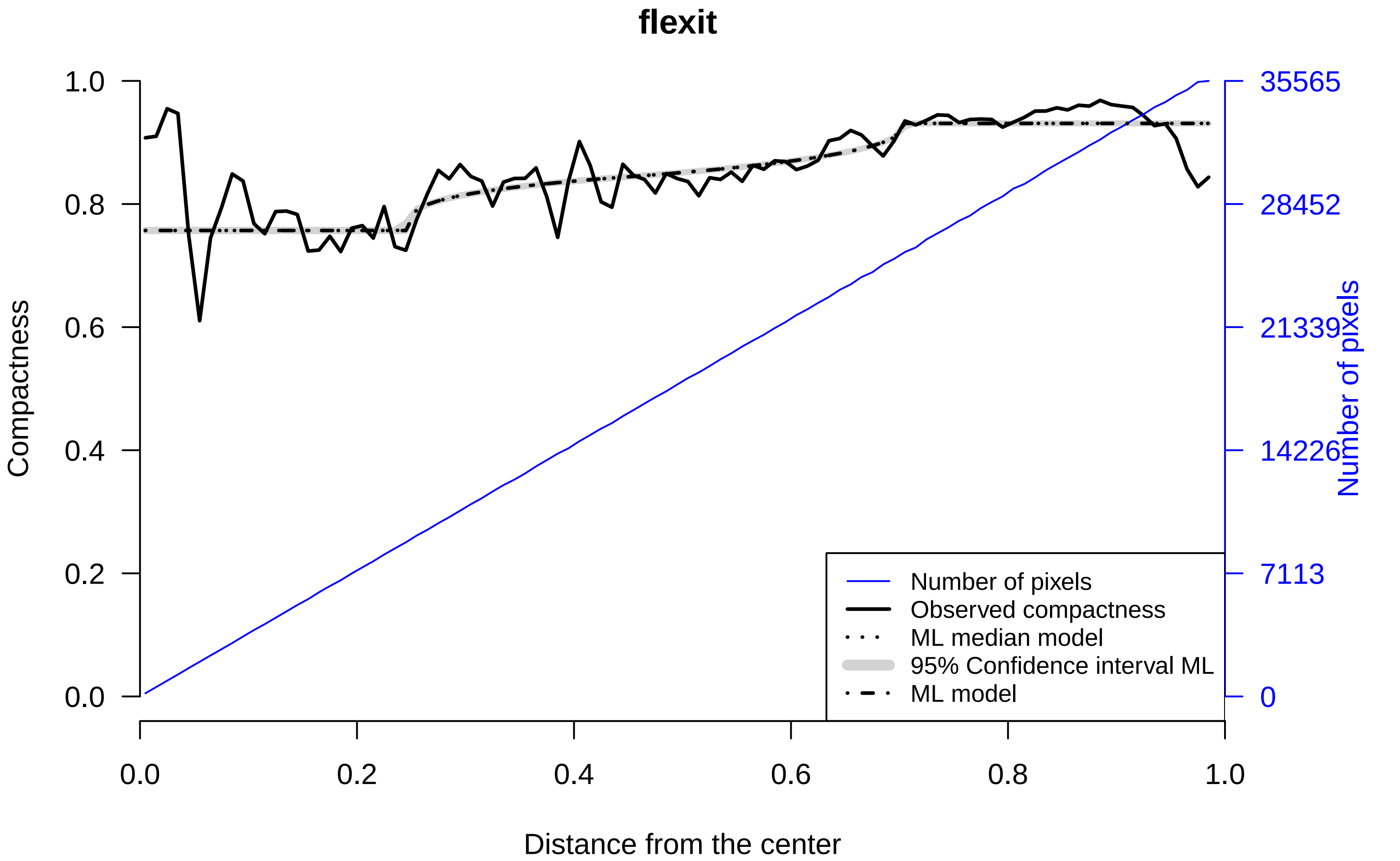

C PMO 222.667
Keilhauia sp. Distal

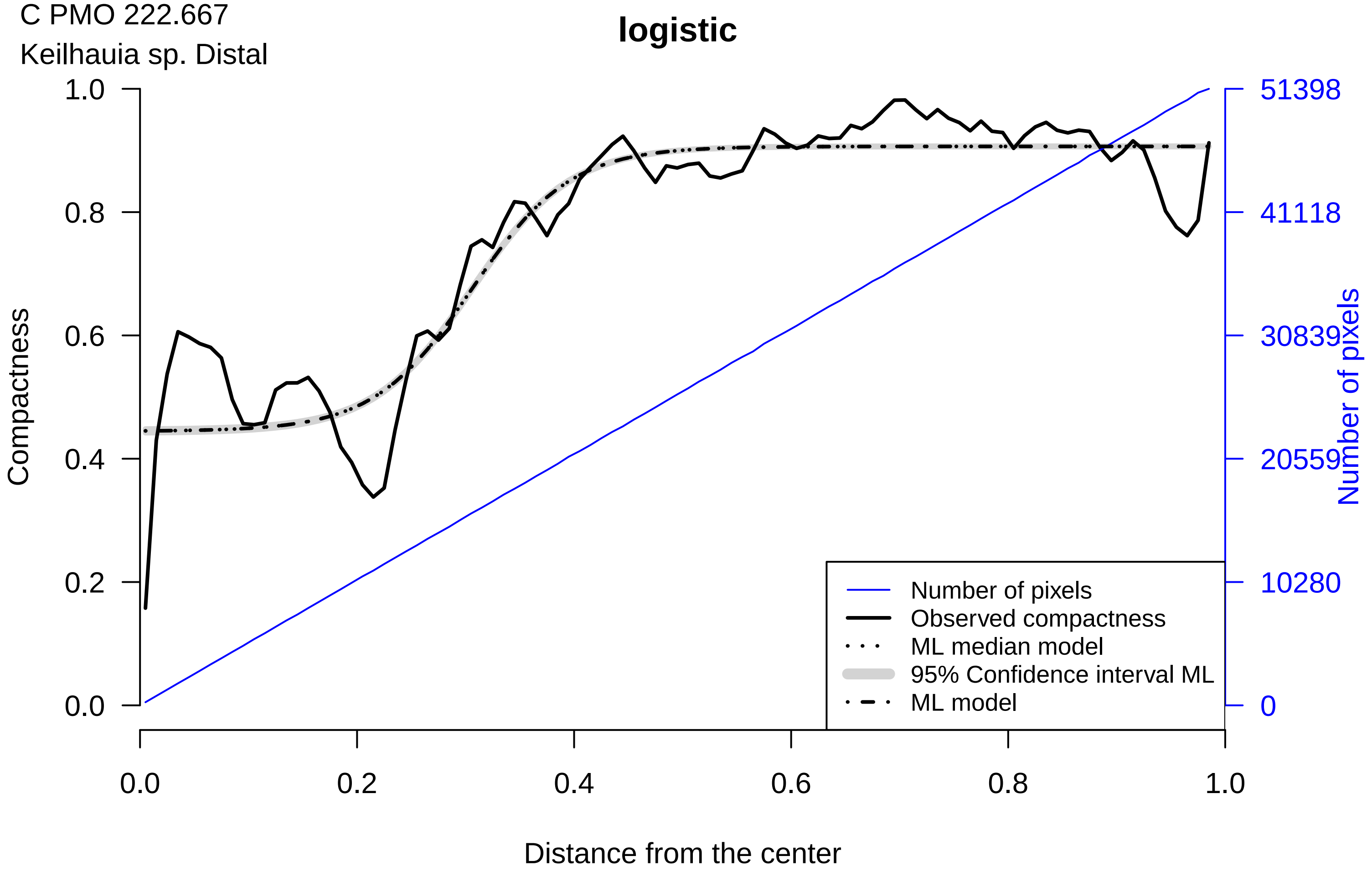

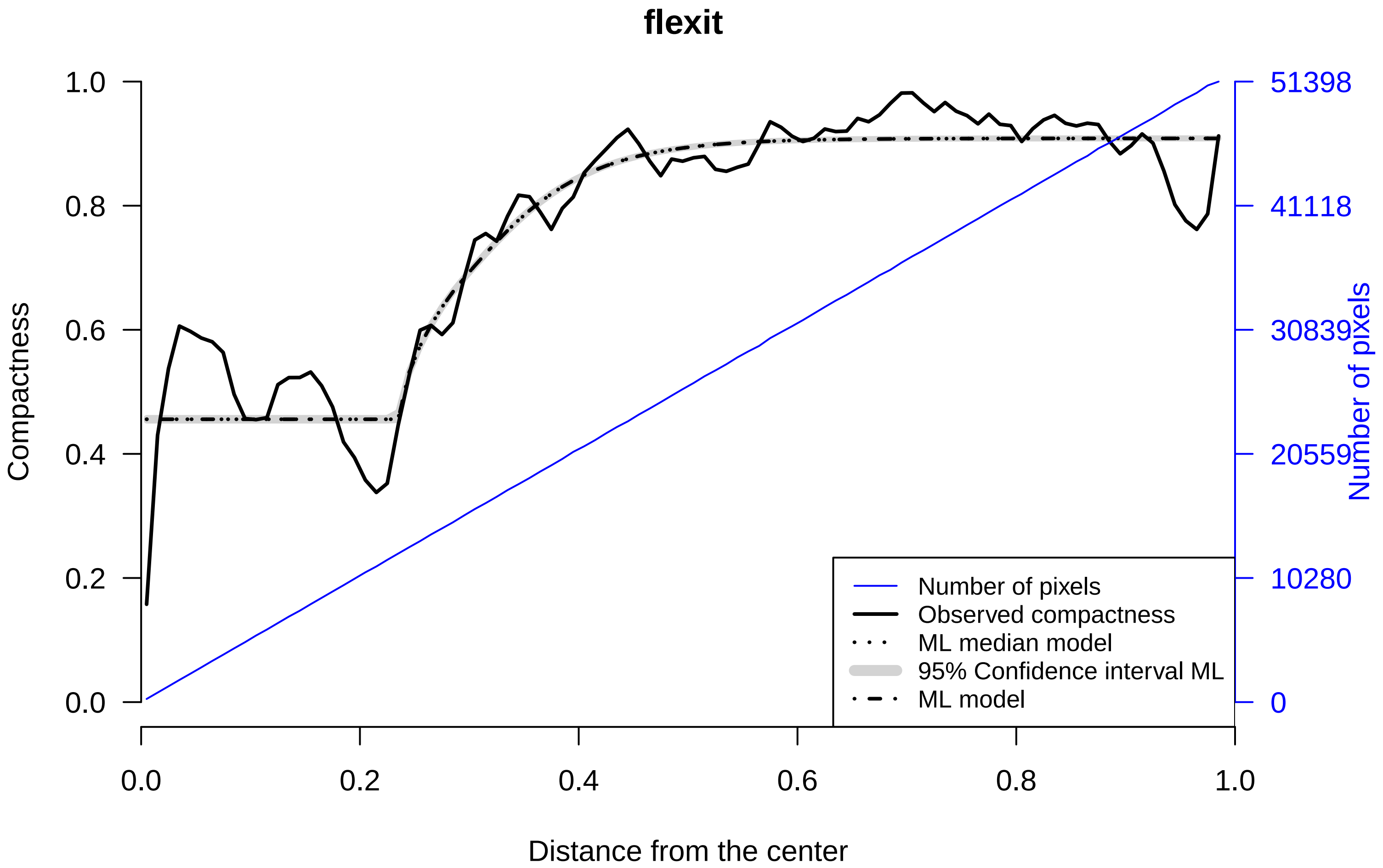

D PMO 222.669
Palvennia hoybergeti Proximal

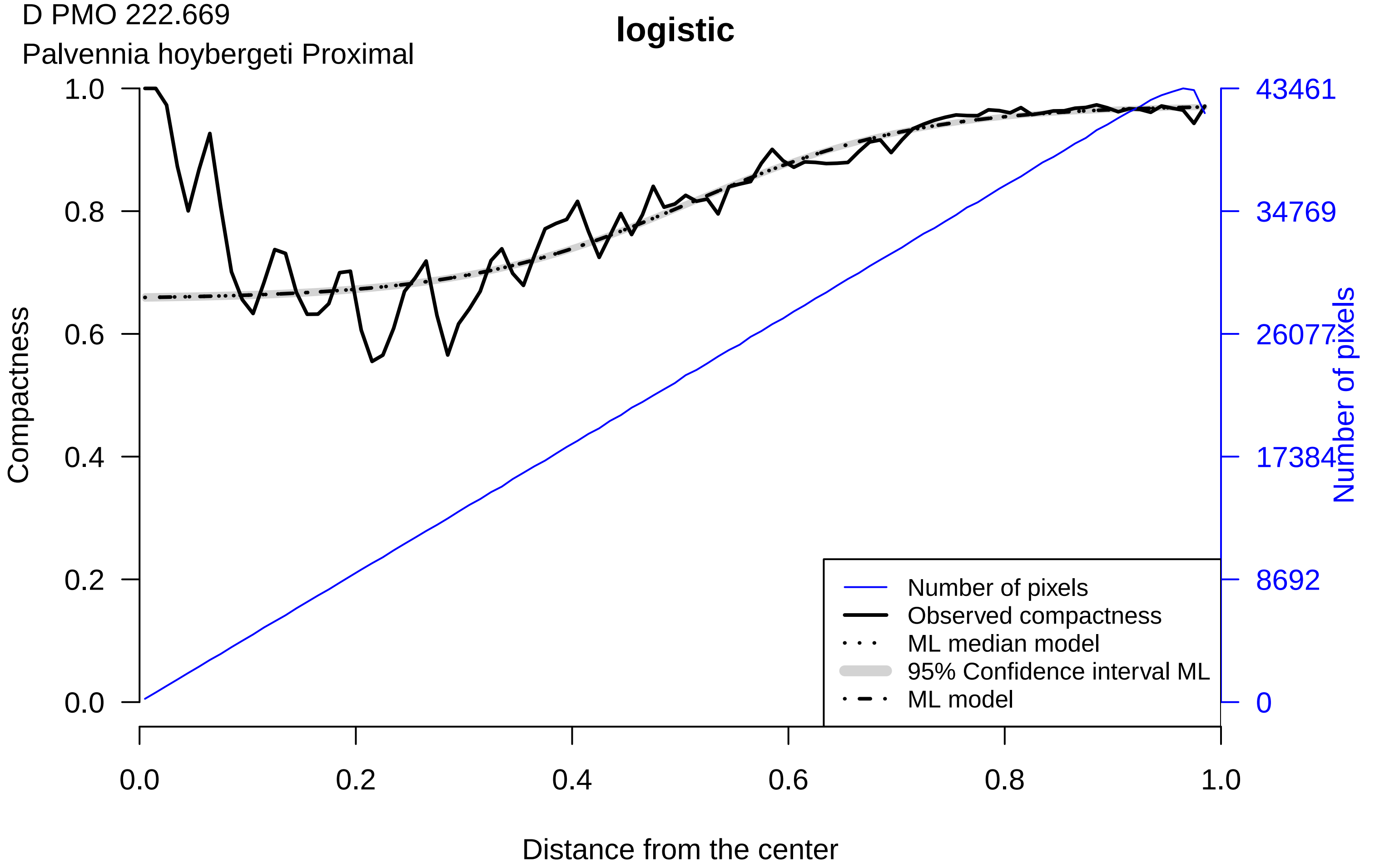

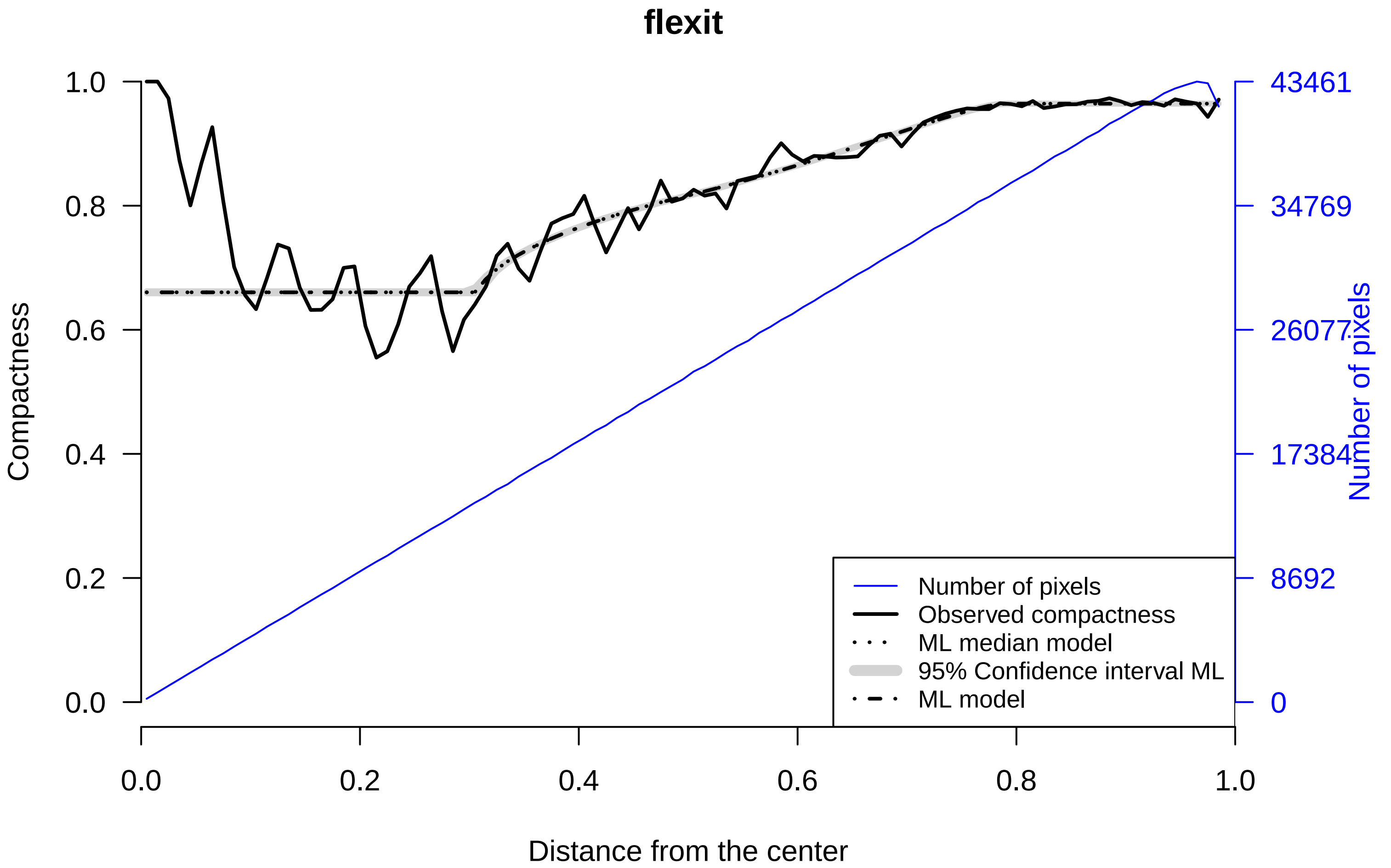

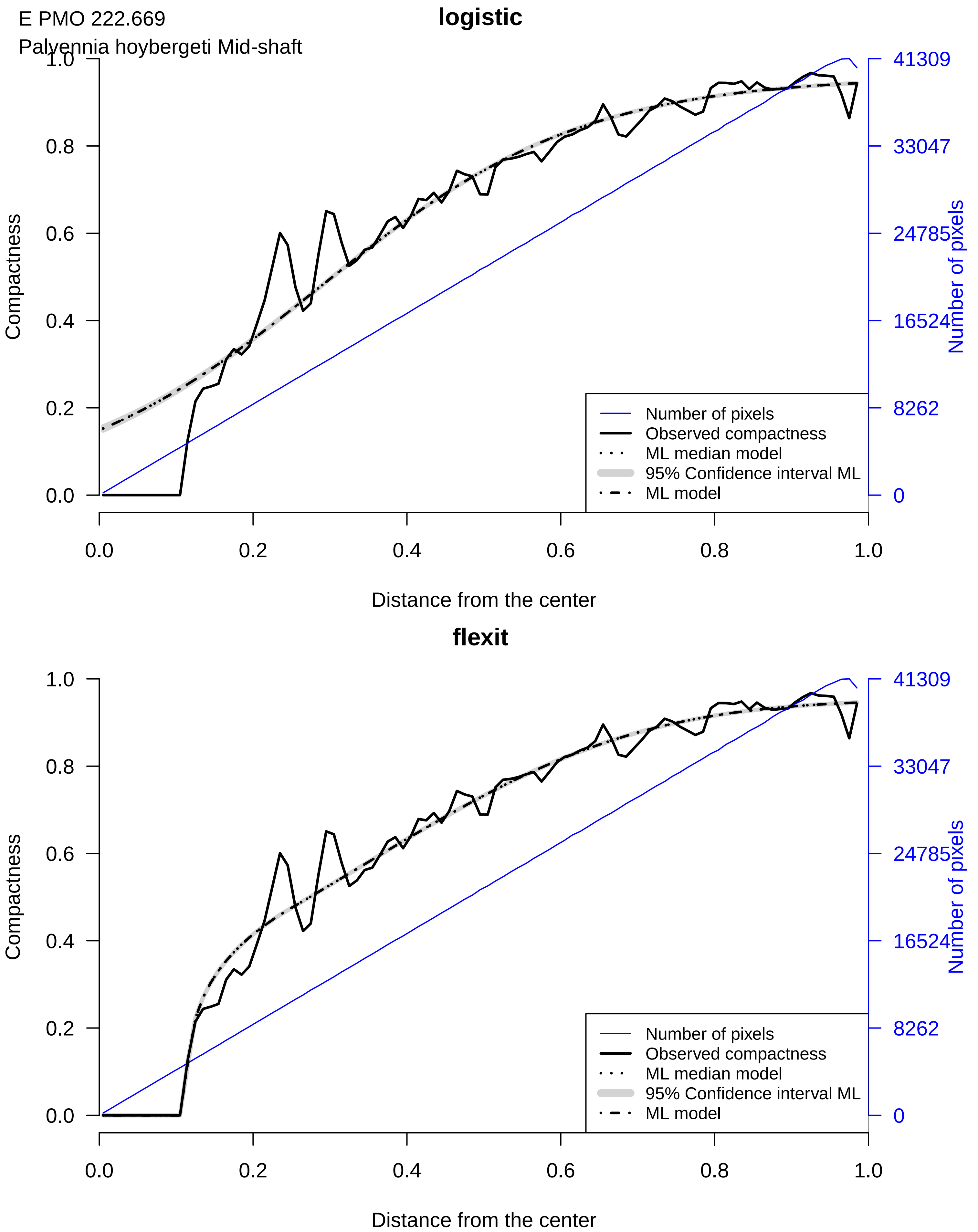
E PMO 222.669
Palyennia hoybergeti Mid-shaft
logistic
Compactness
Number of pixels
Distance from the center
0.0
0.2
0.4
0.6
0.8
1.0
0
8262
16524
24785
33047
41309
Number of pixels
Observed compactness
ML median model
95% Confidence interval ML
ML model
flexit
Compactness
Number of pixels
Distance from the center
0.0
0.2
0.4
0.6
0.8
1.0
0
8262
16524
24785
33047
41309
Number of pixels
Observed compactness
ML median model
95% Confidence interval ML
ML model

F PMO 222.669

Palvennia hoybergeti Distal

logistic

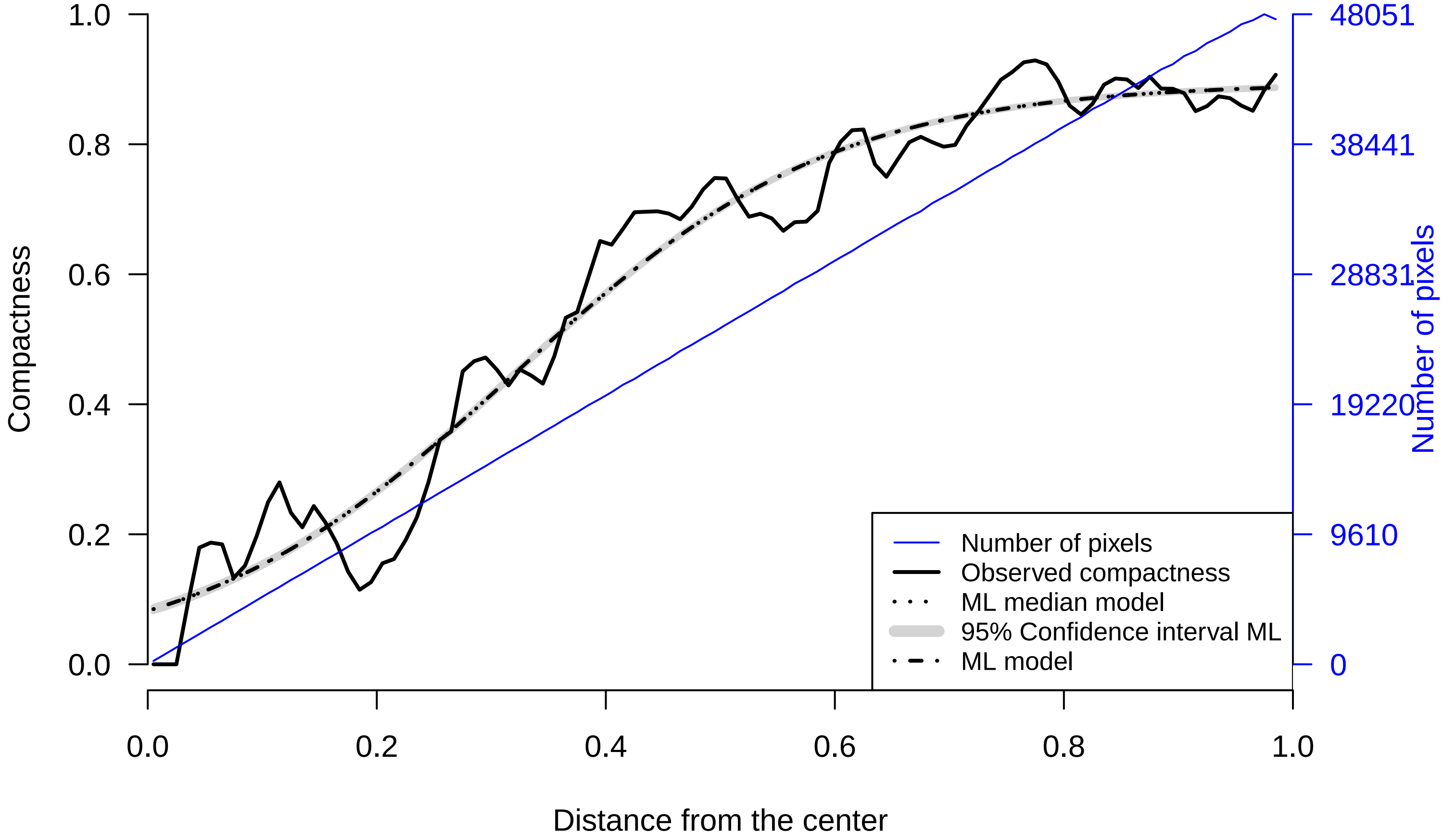

flexit

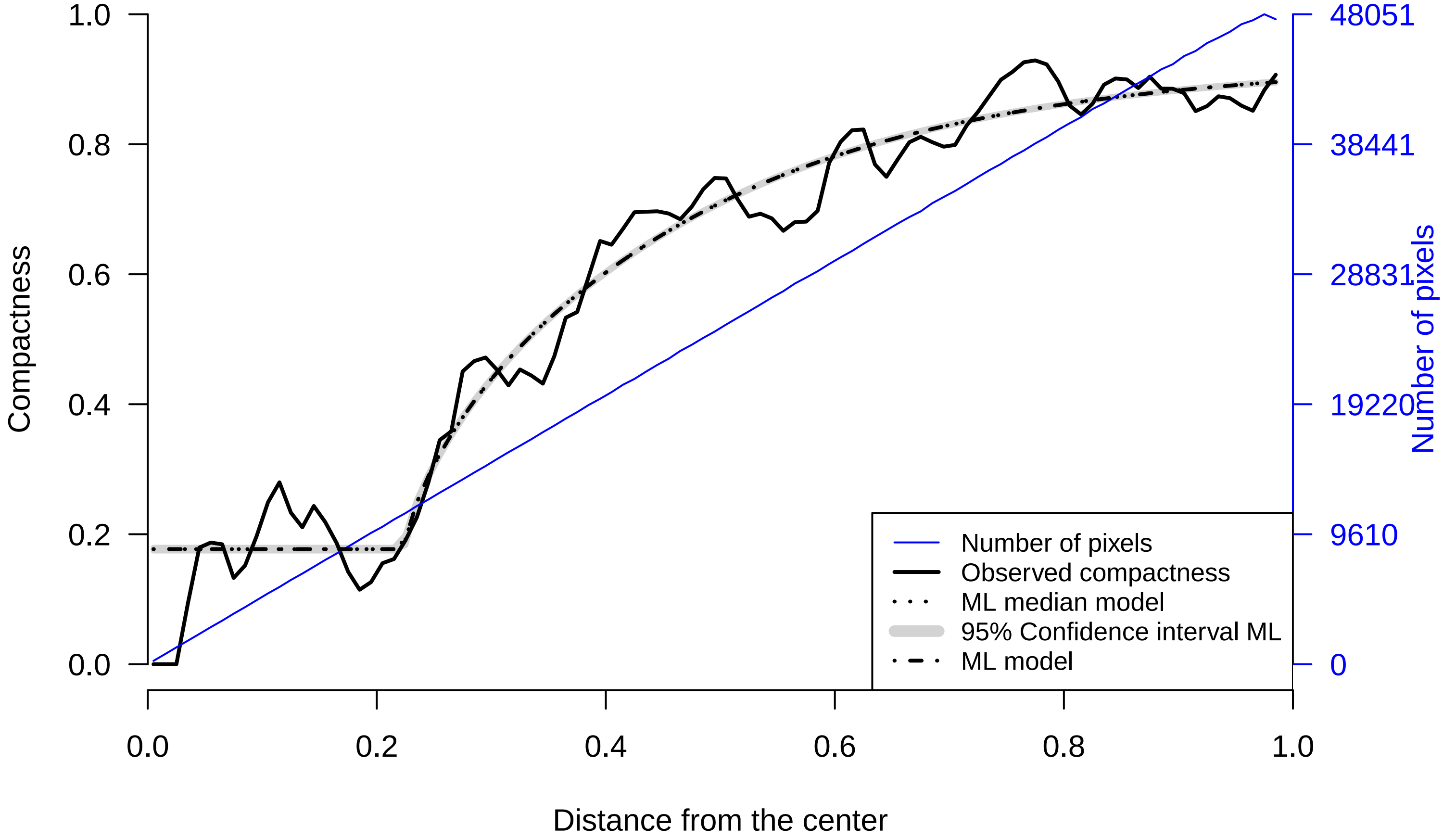

G NHMO-DMA 42918
Phocoena phocoena Proximal

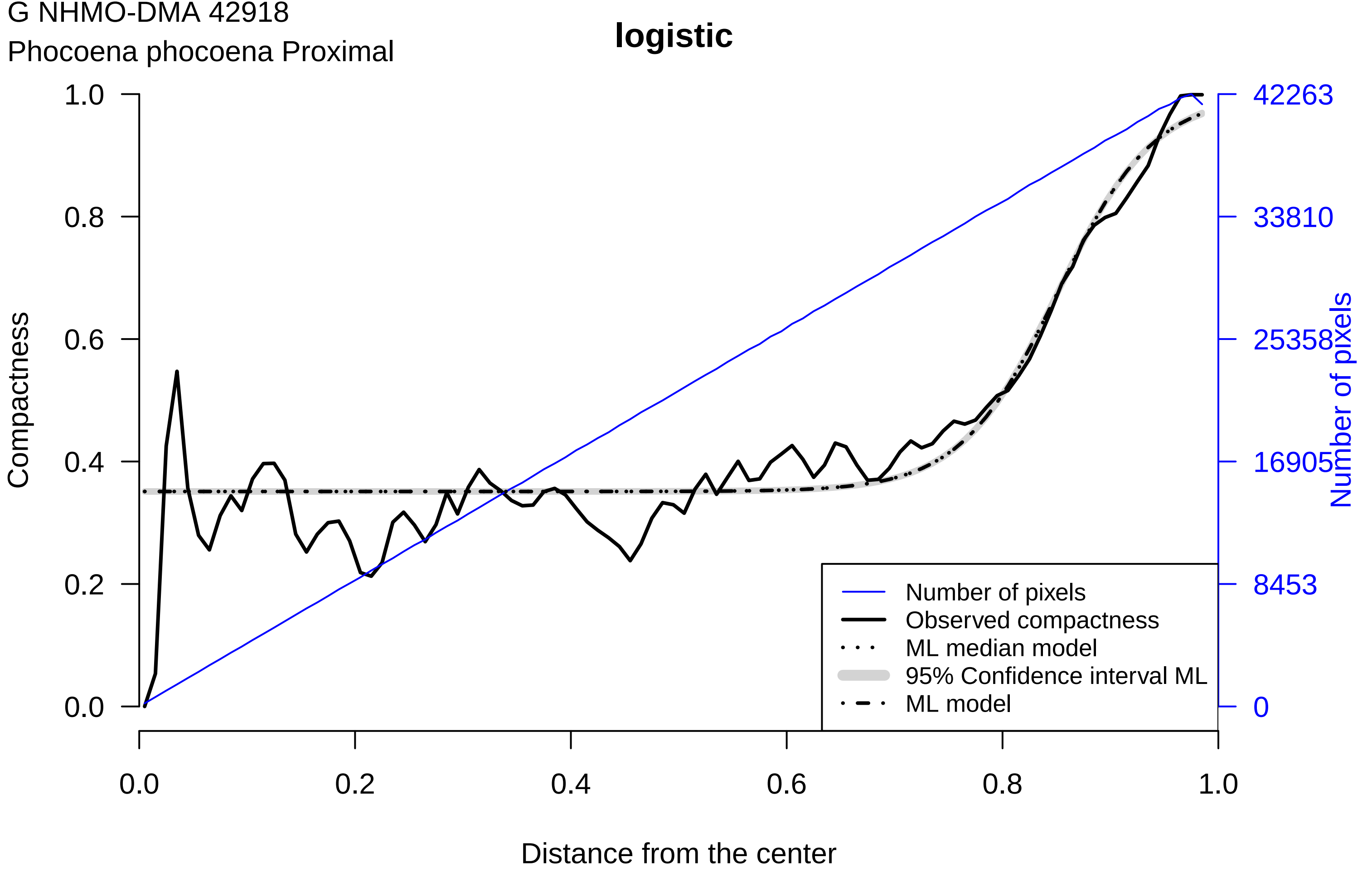

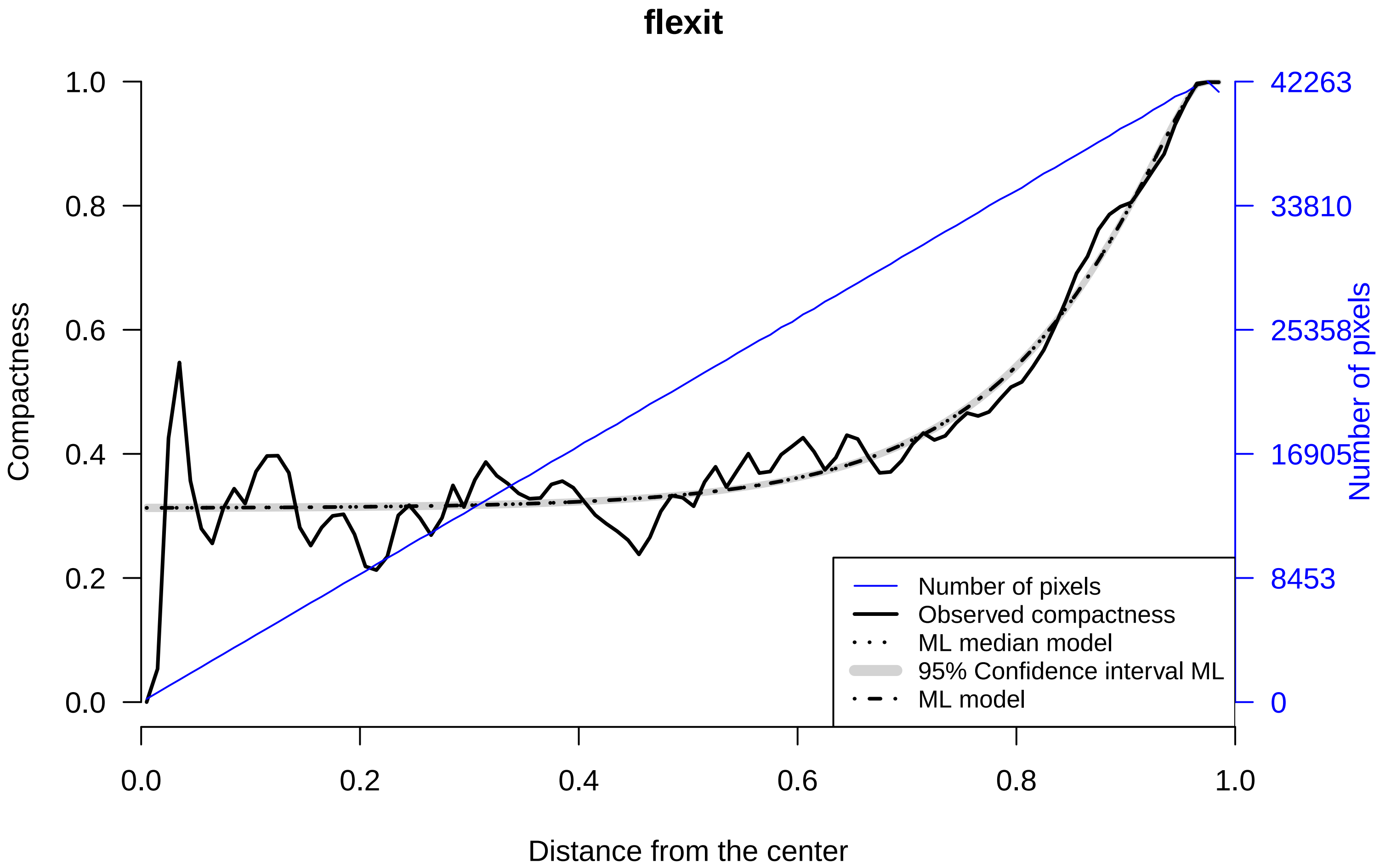

H NHMO-DMA 42918
Phocoena phocoena Mid-shaft

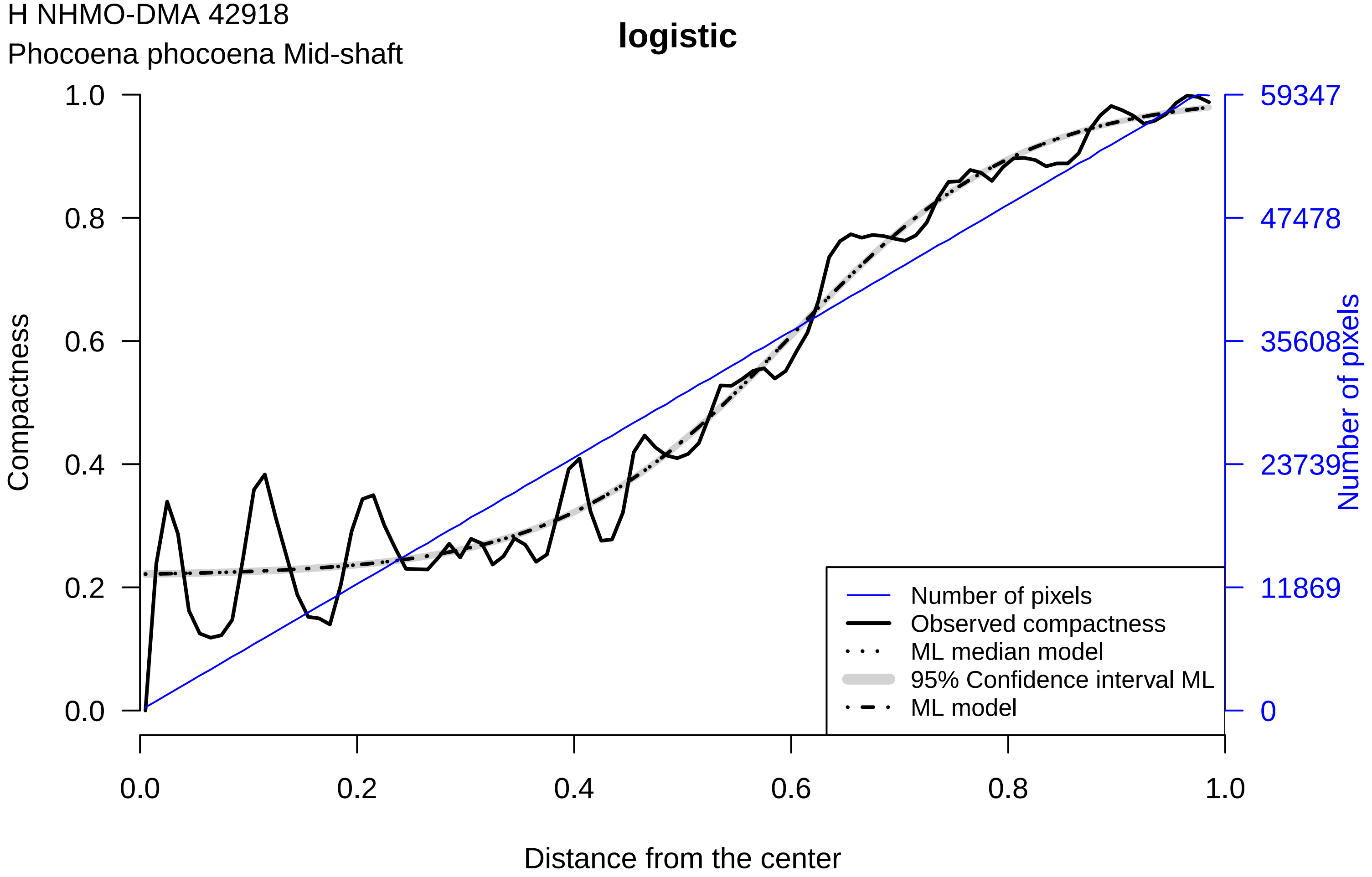

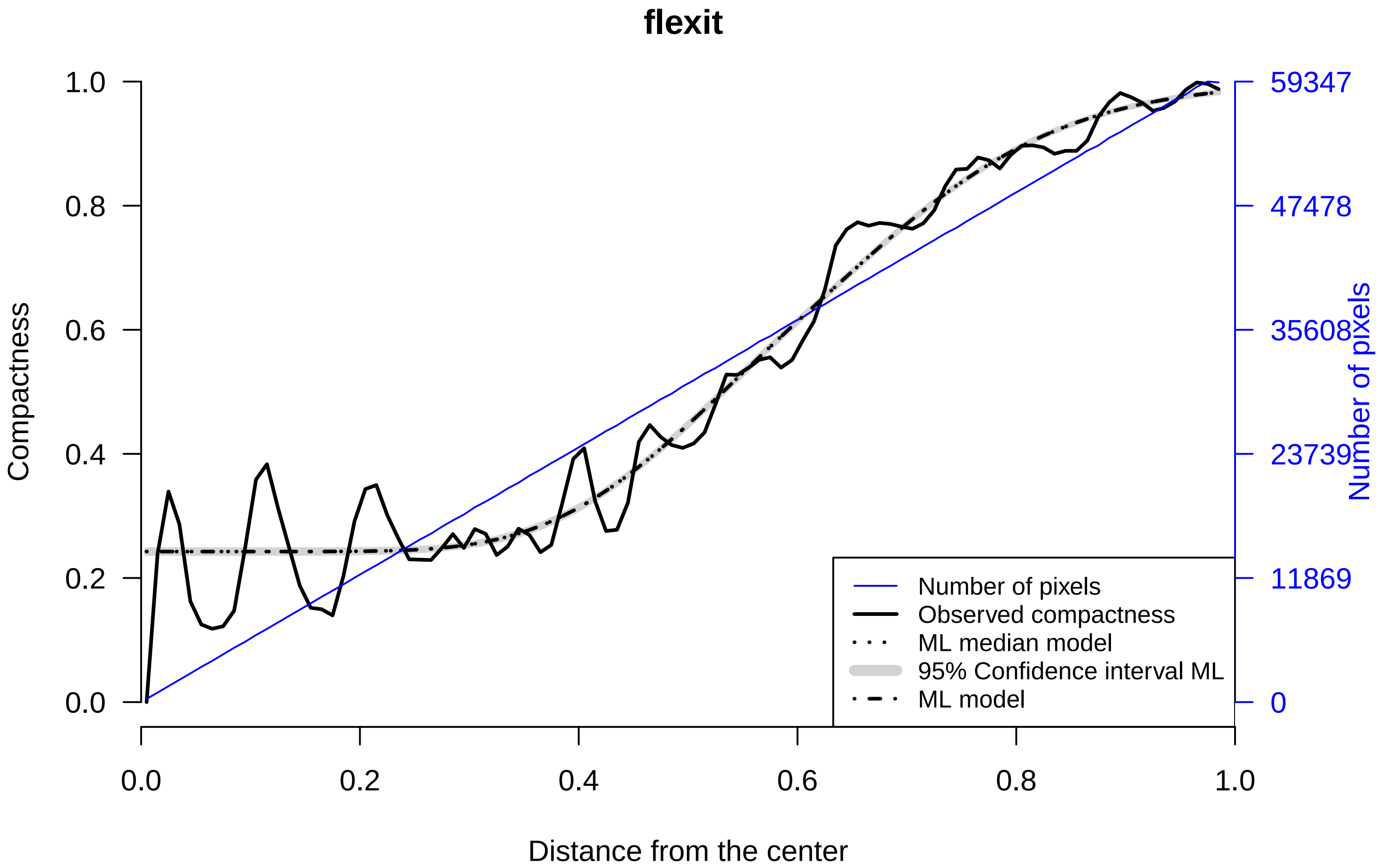

I NHMO-DMA 42918
Phocoena phocoena Distal

logistic

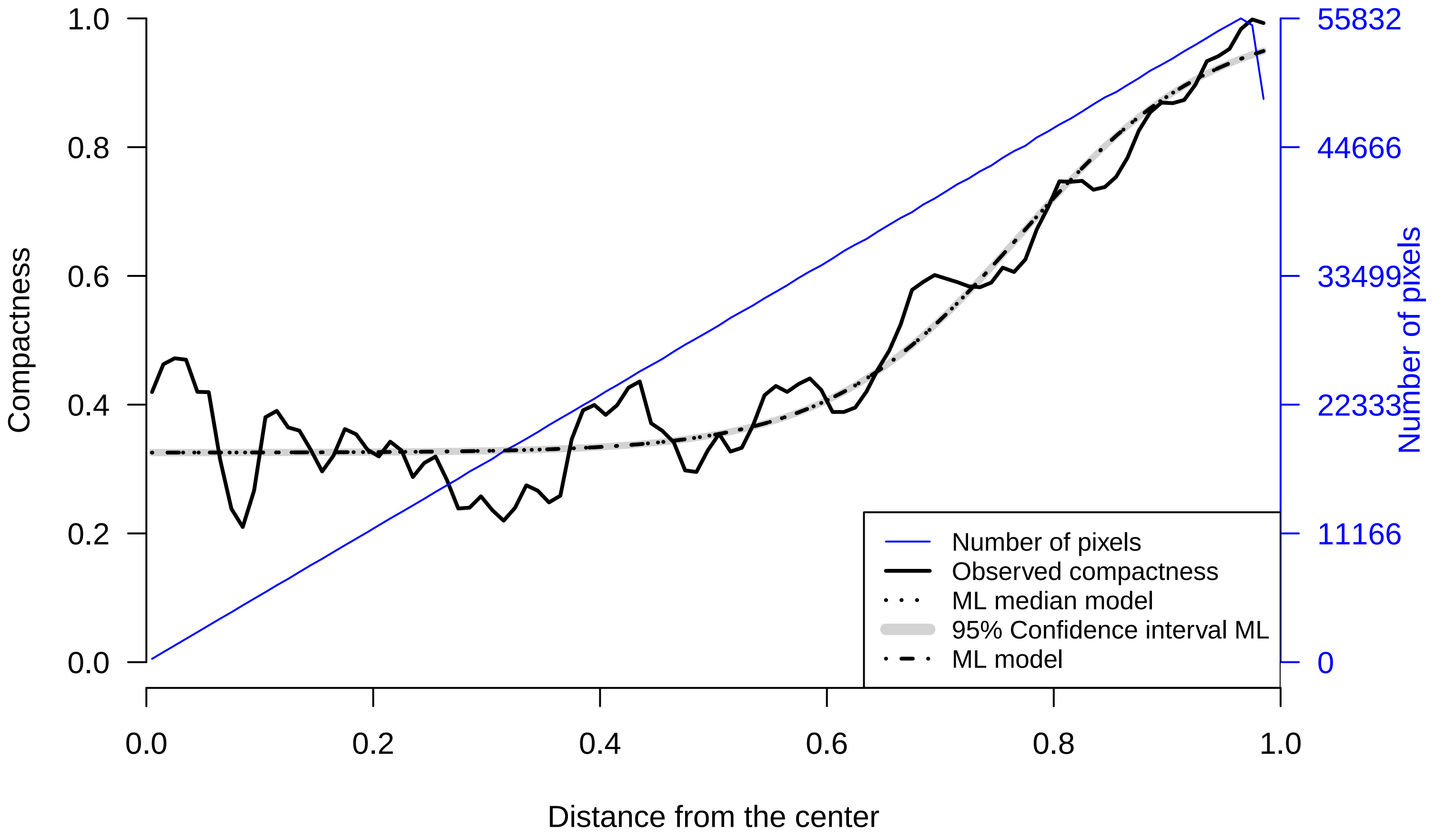

flexit

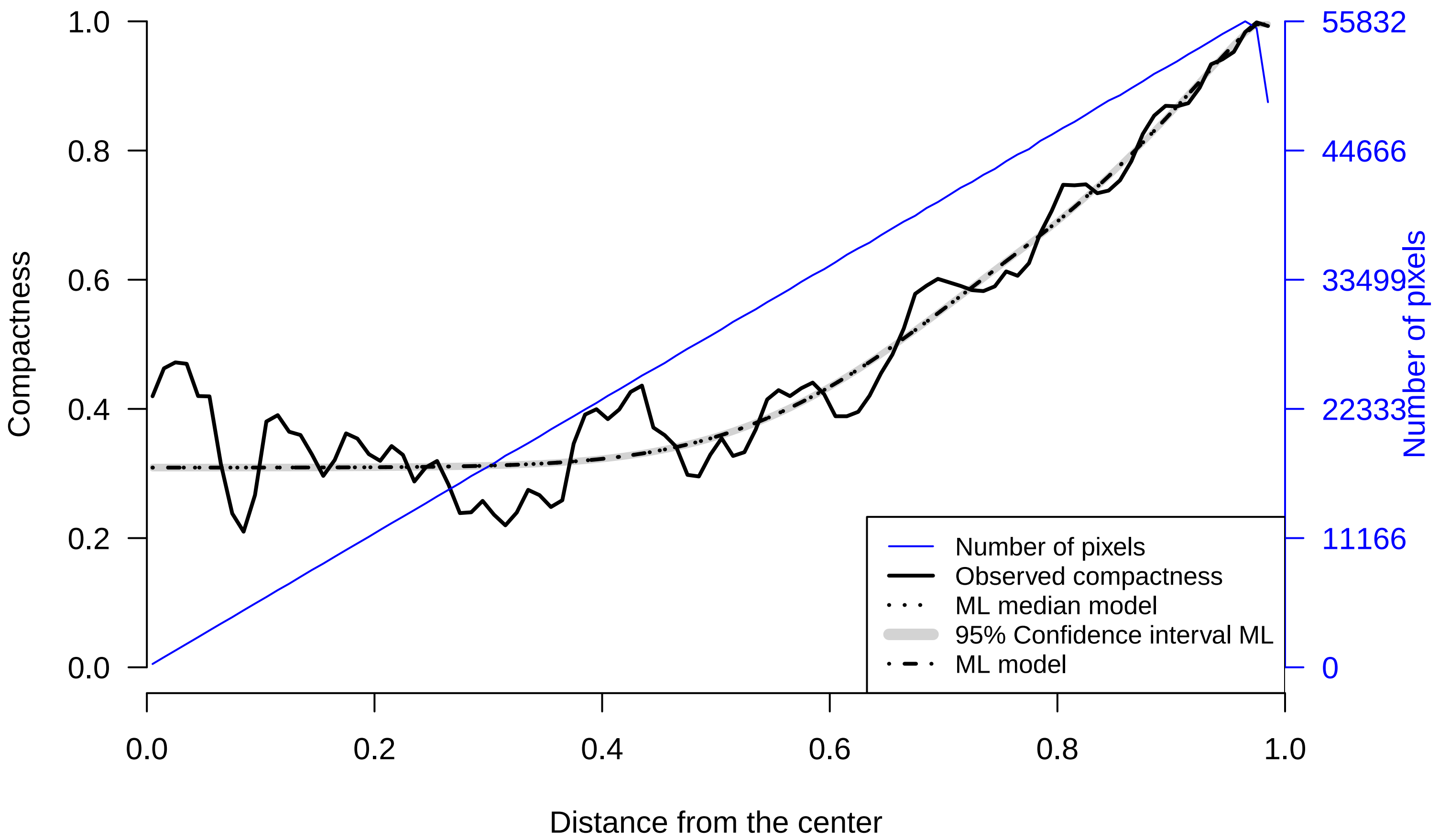

J NHMO-DMA 32051
Delphinapterus leucas Proximal

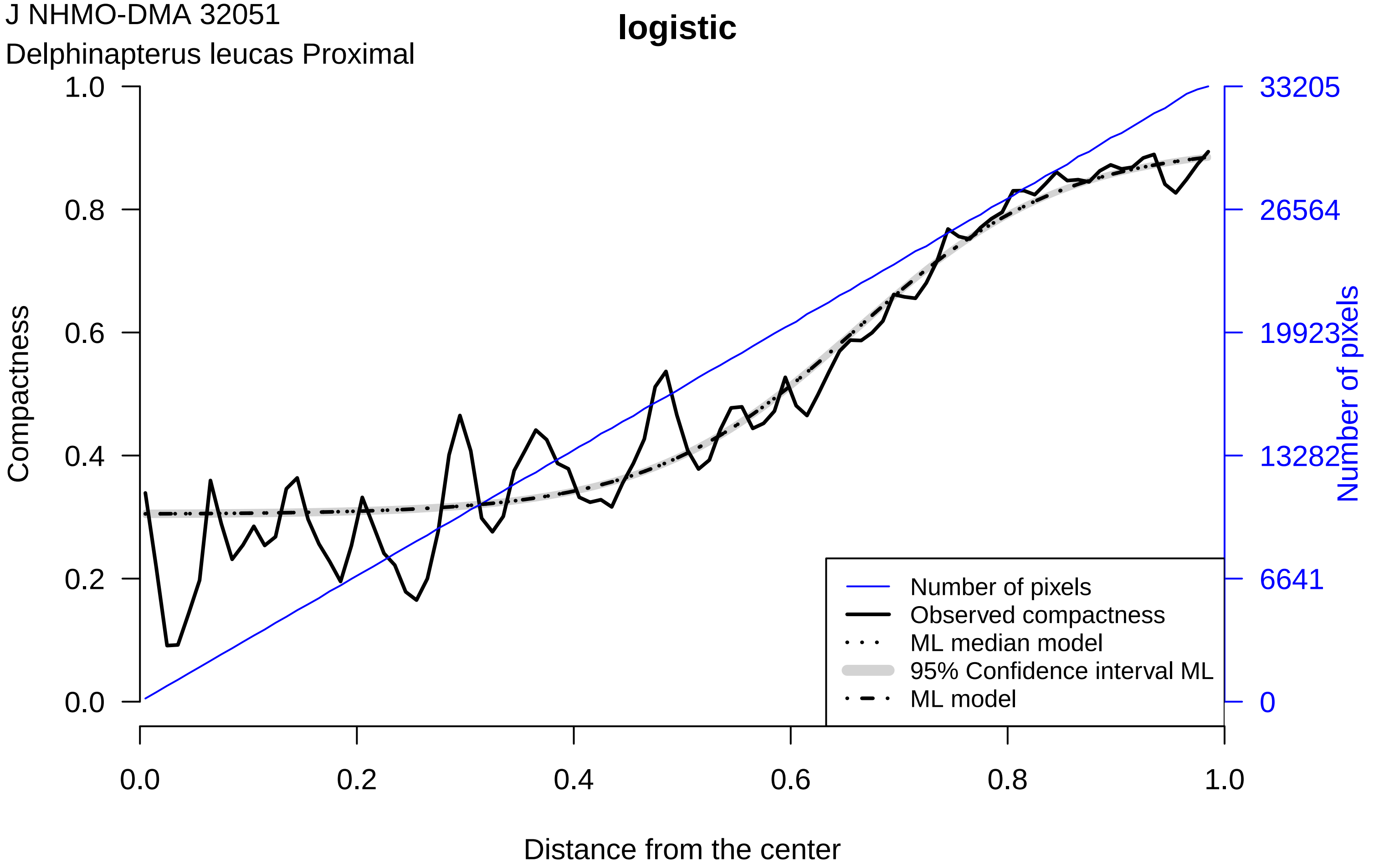

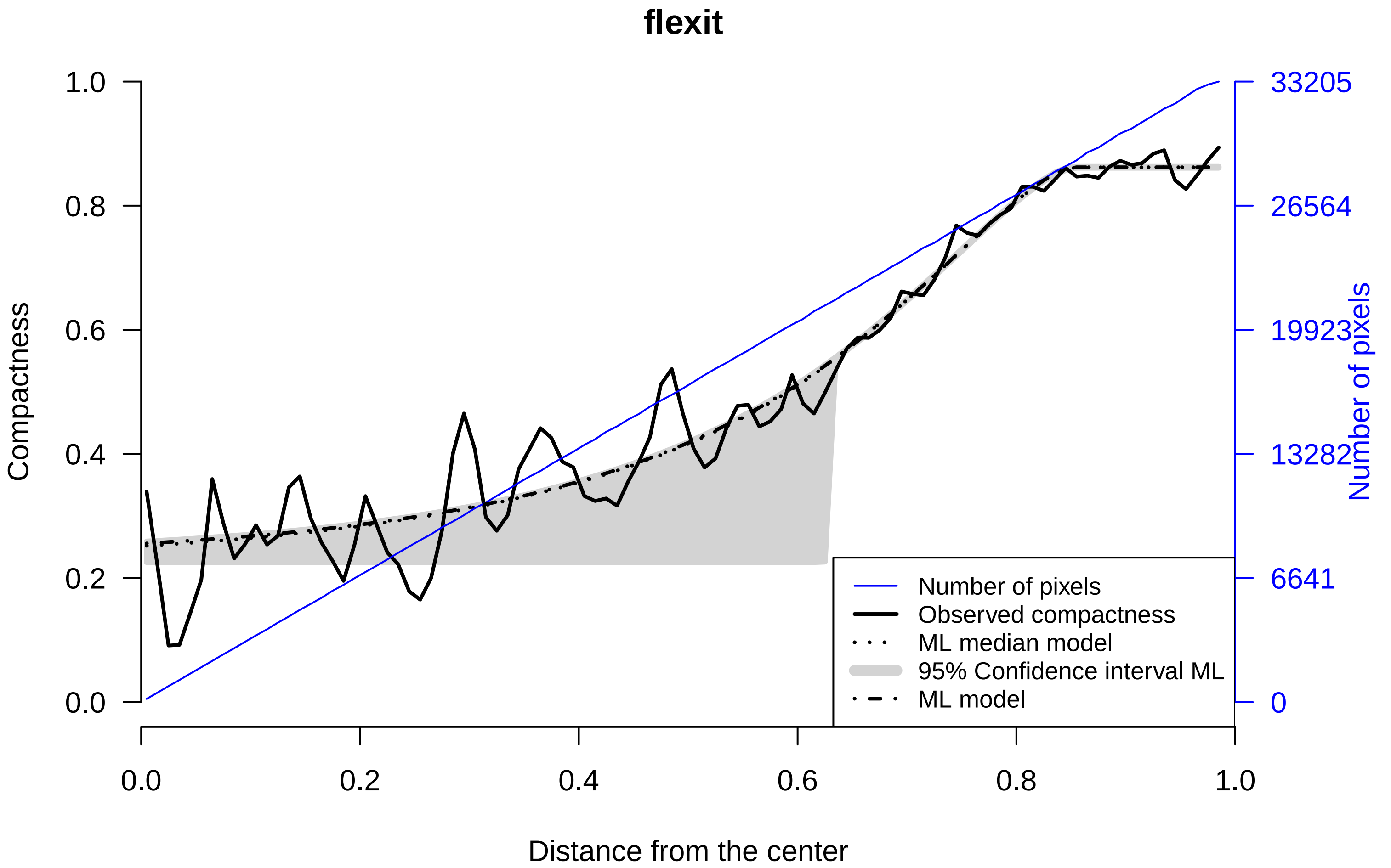

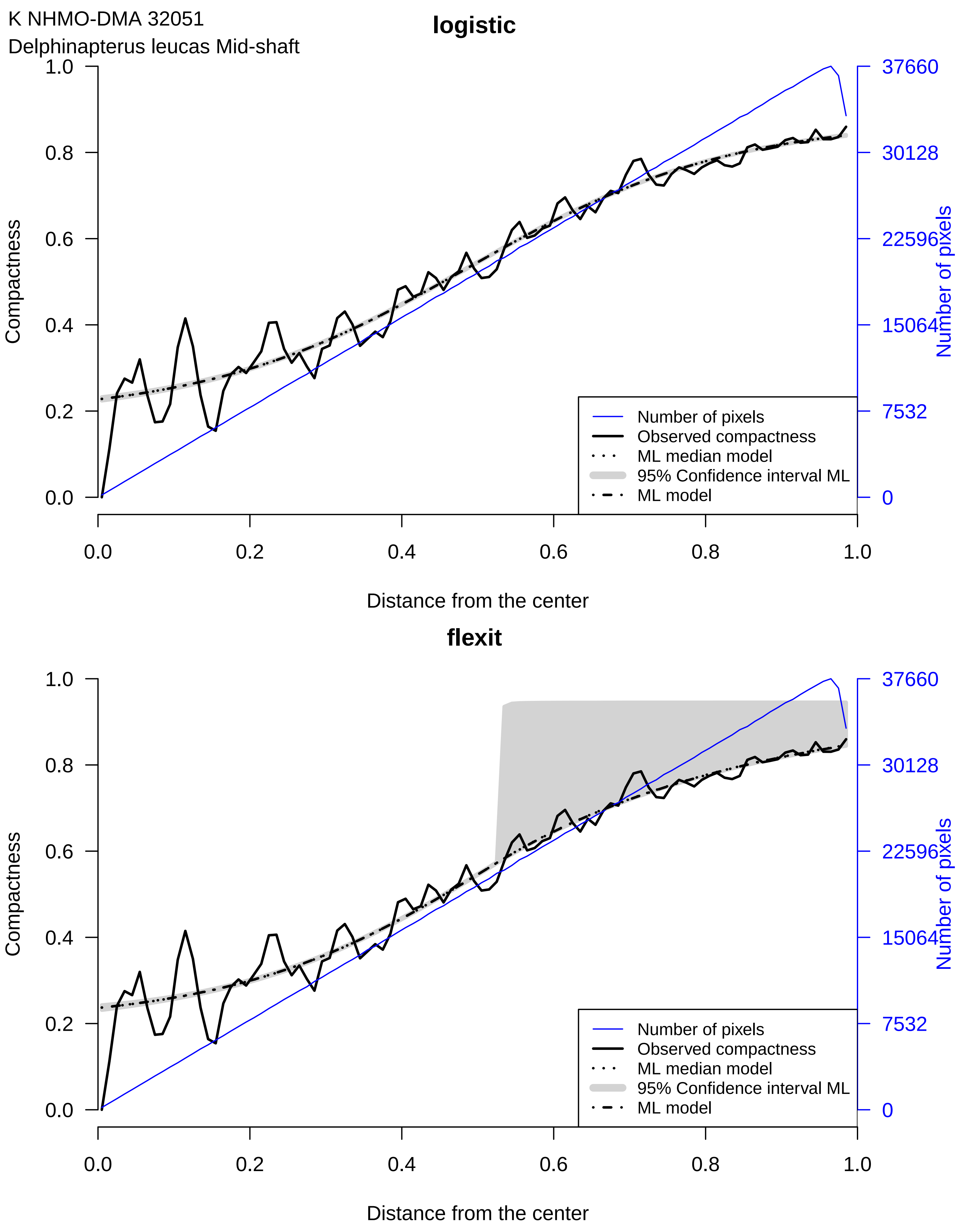
K NHMO-DMA 32051
Delphinapterus leucas Mid-shaft
logistic
Compactness
Number of pixels
0.0
0.2
0.4
0.6
0.8
1.0
0
7532
15064
22596
30128
37660
Number of pixels
Observed compactness
ML median model
95% Confidence interval ML
ML model
Distance from the center
flexit
Compactness
Number of pixels
0.0
0.2
0.4
0.6
0.8
1.0
0
7532
15064
22596
30128
37660
Number of pixels
Observed compactness
ML median model
95% Confidence interval ML
ML model
Distance from the center

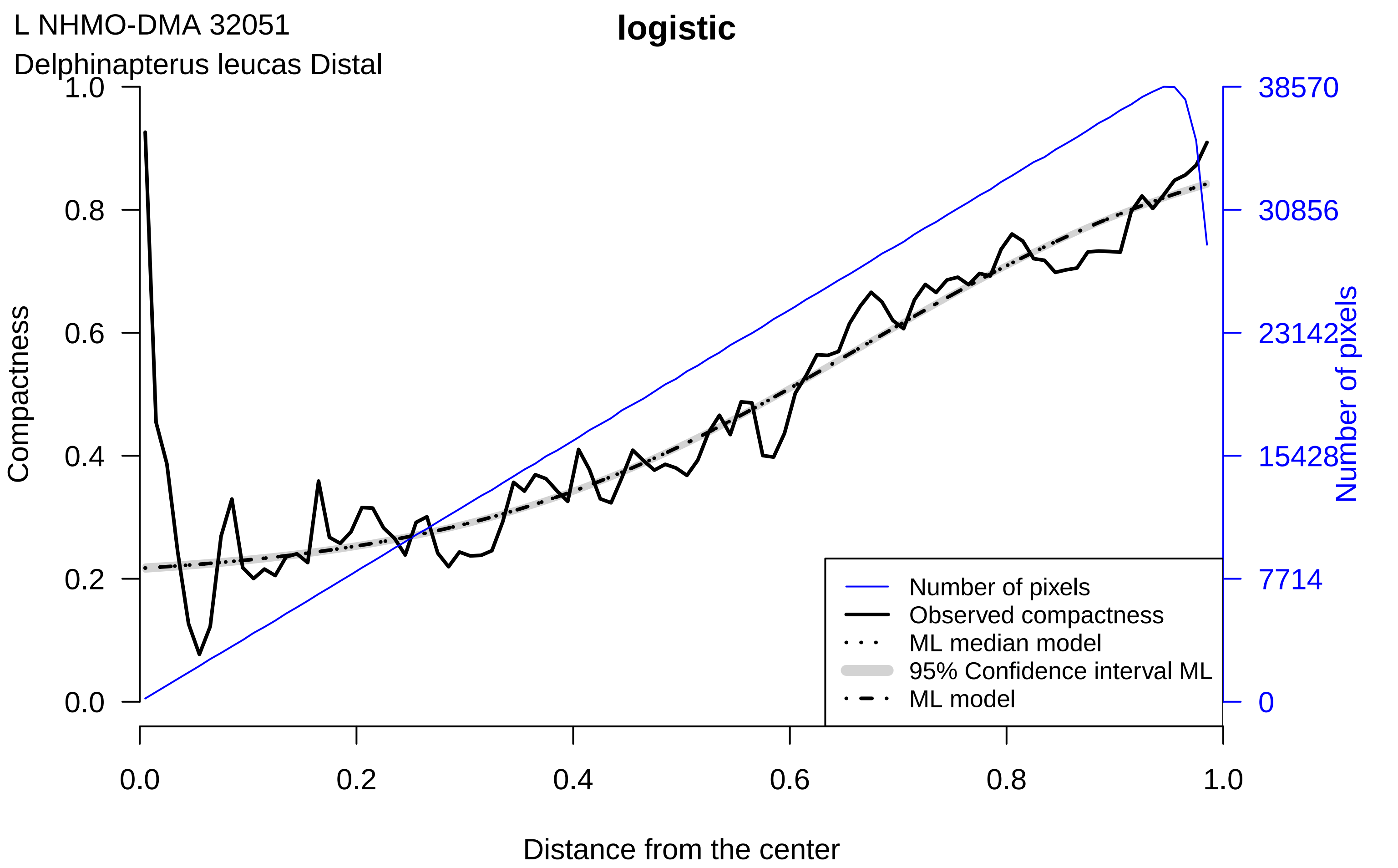
L NHMO-DMA 32051
Delphinapterus leucas Distal
logistic
Compactness
Number of pixels
Distance from the center
1.0
0.8
0.6
0.4
0.2
0.0
38570
30856
23142
15428
7714
0
Number of pixels
Observed compactness
ML median model
95% Confidence interval ML
ML model

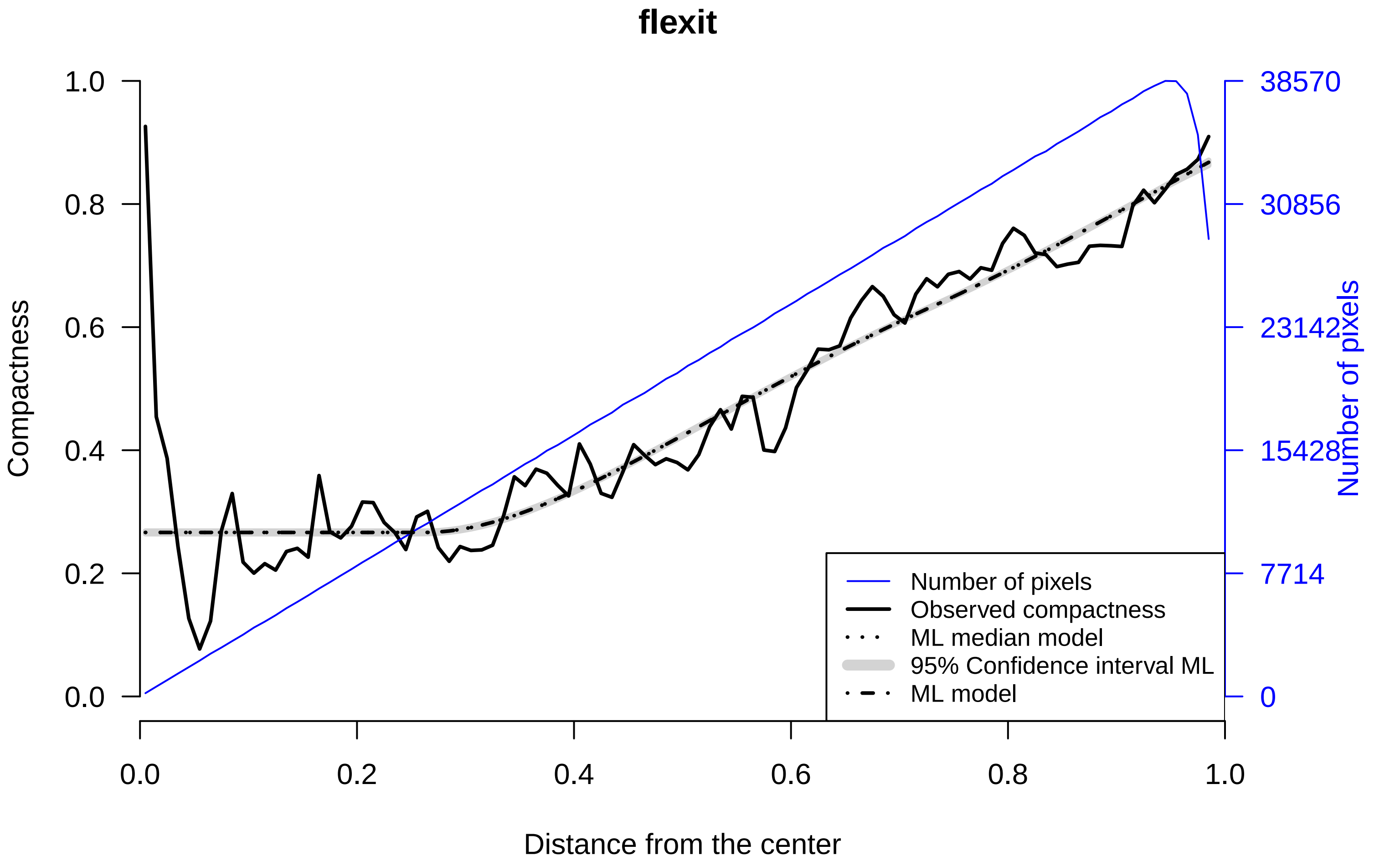
flexit
Compactness
Number of pixels
Distance from the center
1.0
0.8
0.6
0.4
0.2
0.0
38570
30856
23142
15428
7714
0
Number of pixels
Observed compactness
ML median model
95% Confidence interval ML
ML model
