# Supplementary material for: Rib microstructure in thunniform ichthyosaurs and toothed whales
Source: PeerJ. 2026 Jul 7;14:e21486. doi: 10.7717/peerj.21486 (PMC13353231; doi:10.7717/peerj.21486)

Supplemental data S3 Binarized rib sections for data collection using BoneProfiler

PMO 222.667 Proximal

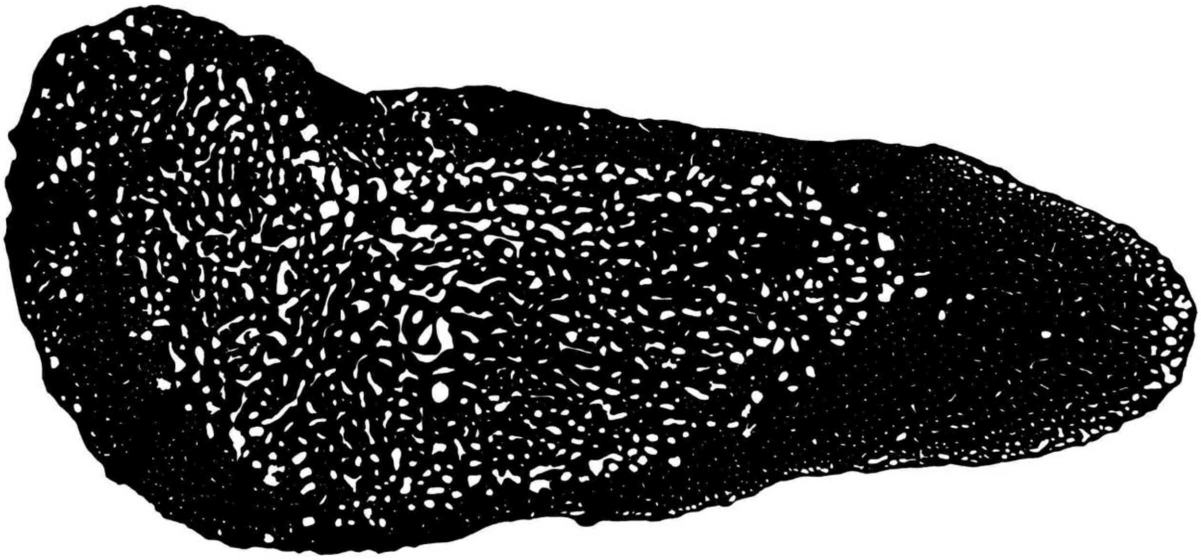

PMO 222.667 Mid-shaft

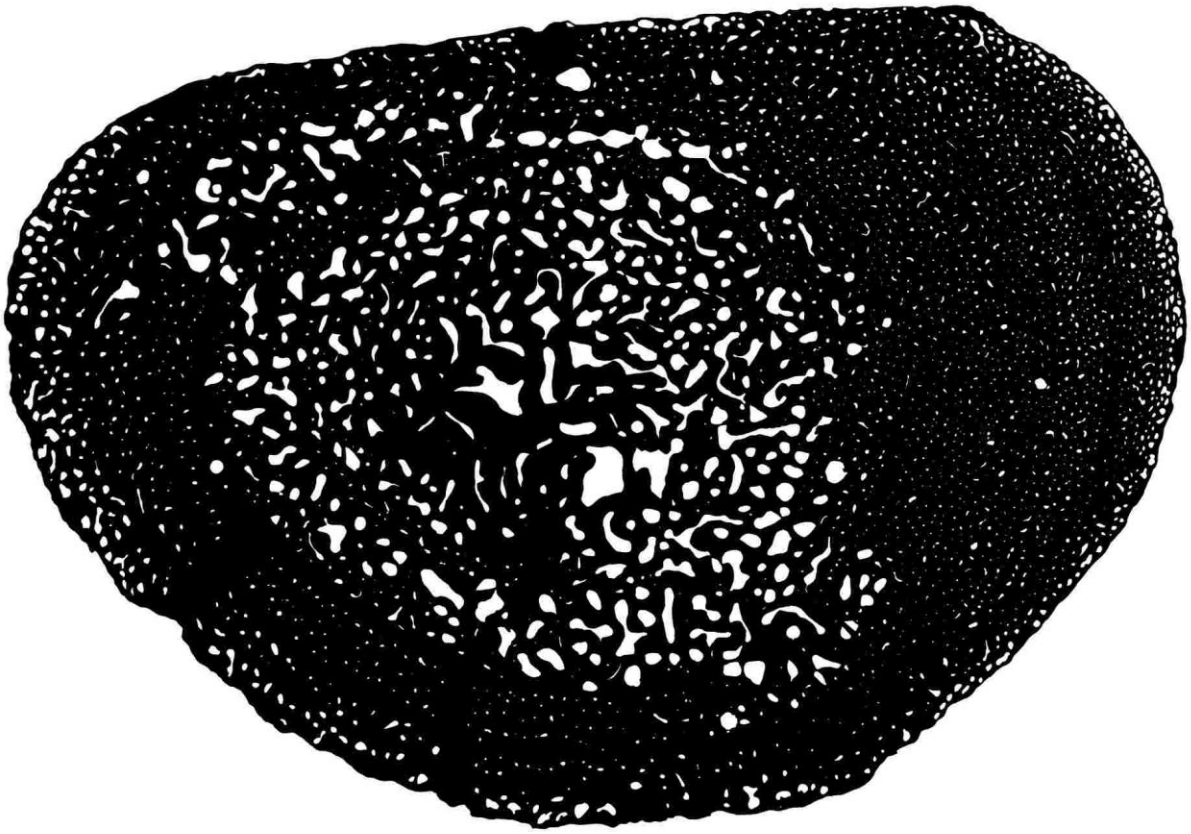

PMO 222.667 Distal

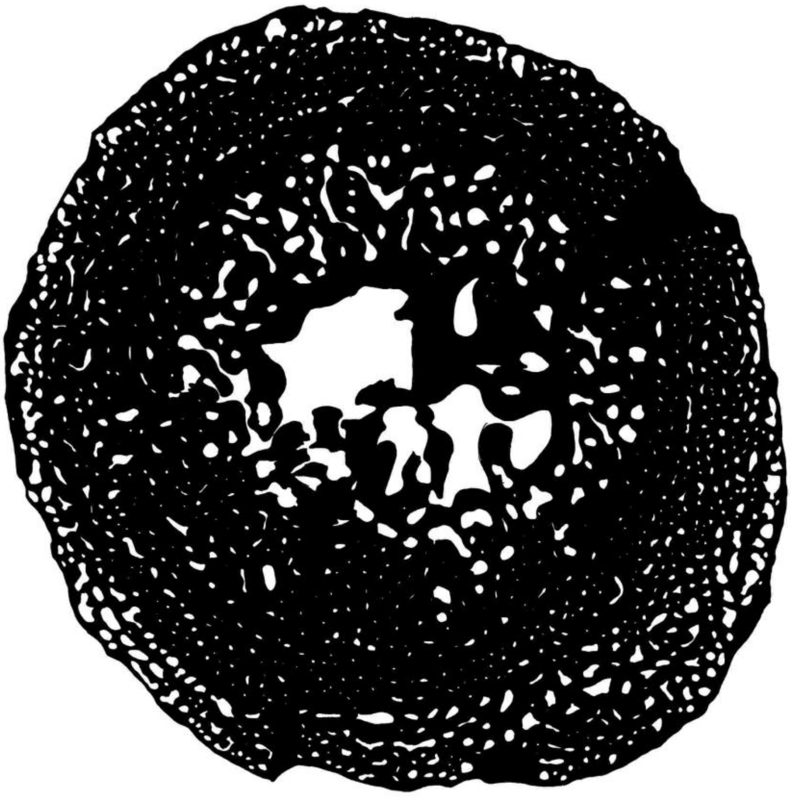

PMO 222.669 Proximal

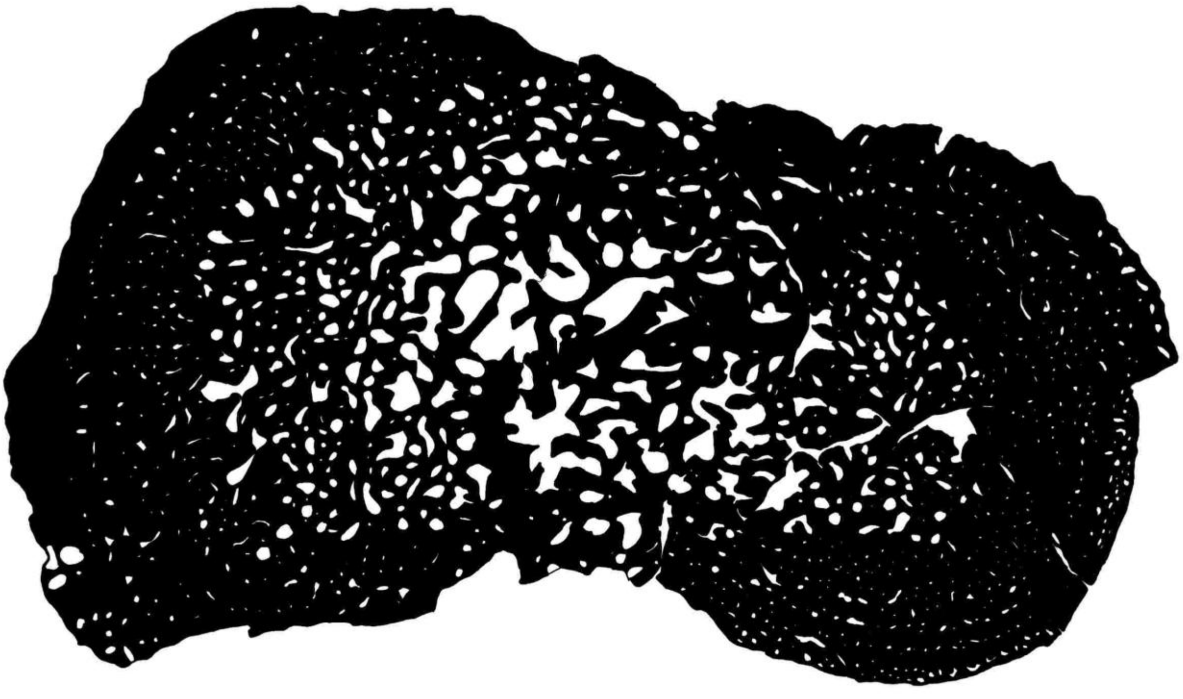

PMO 222.669 Mid-shaft

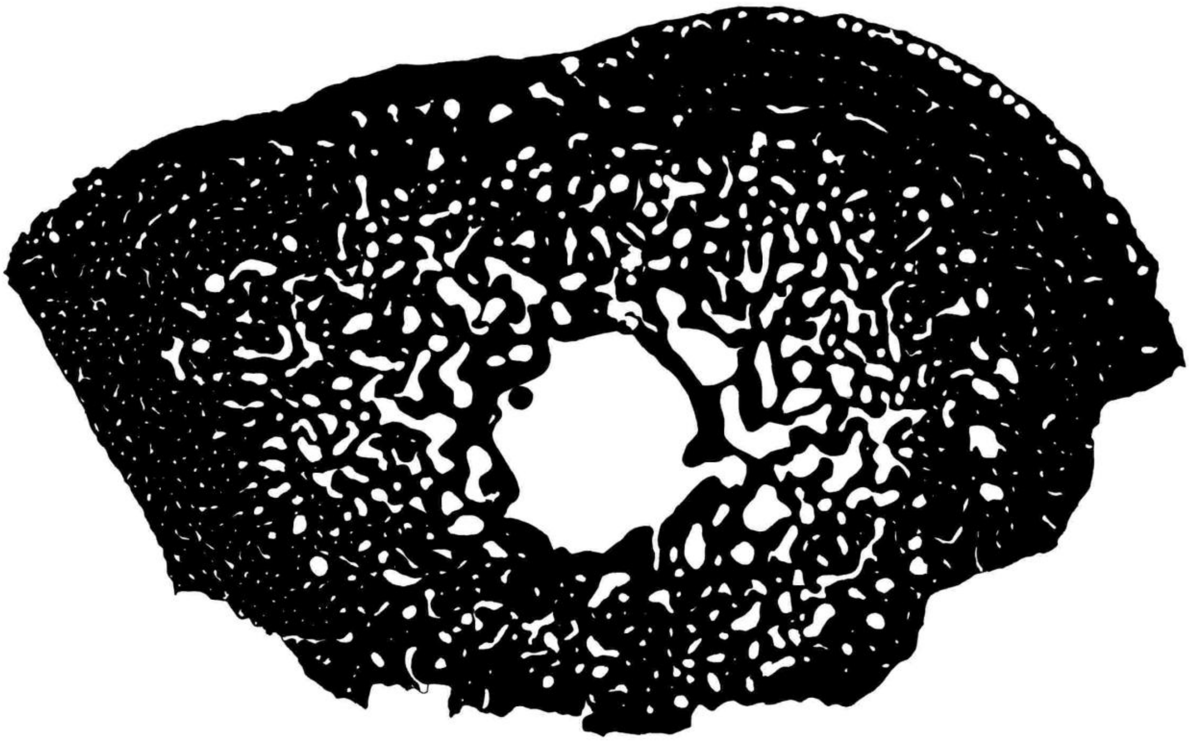

PMO 222.669 Distal

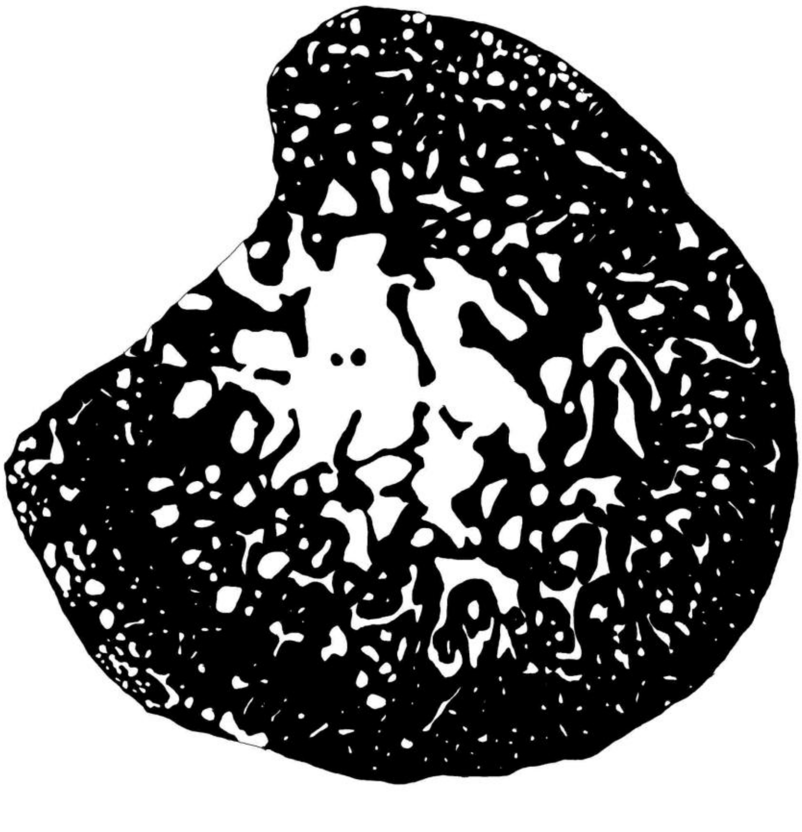

NHMO-DMA-42918 Proximal

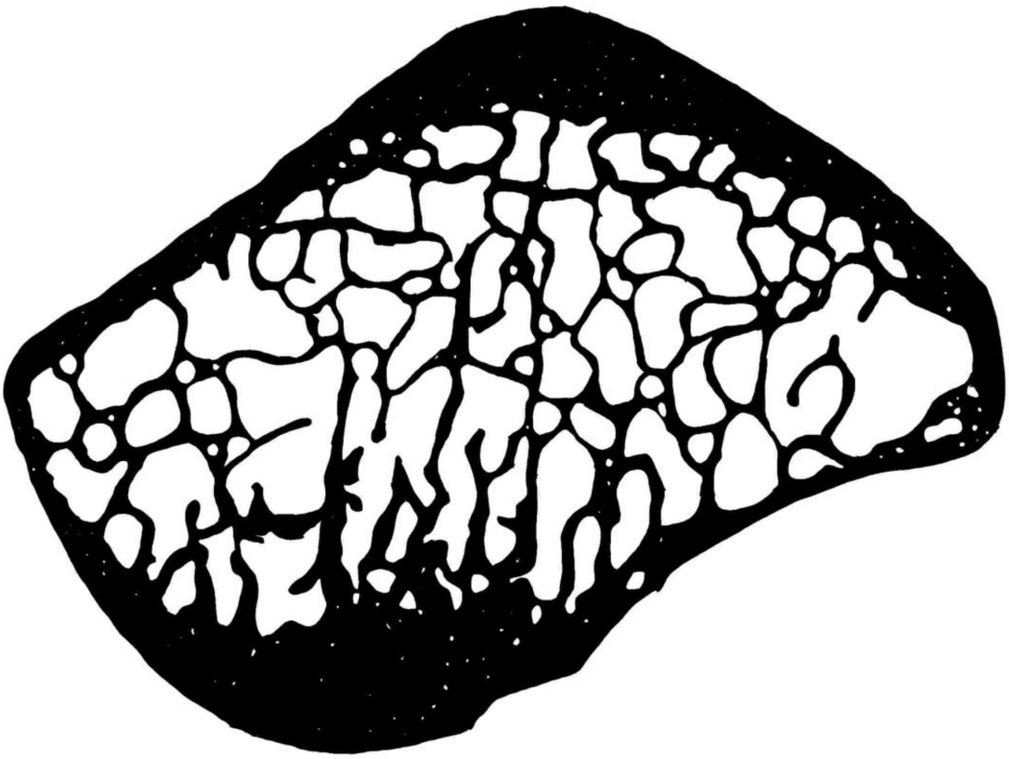

NHMO-DMA-42918 Mid-shaft

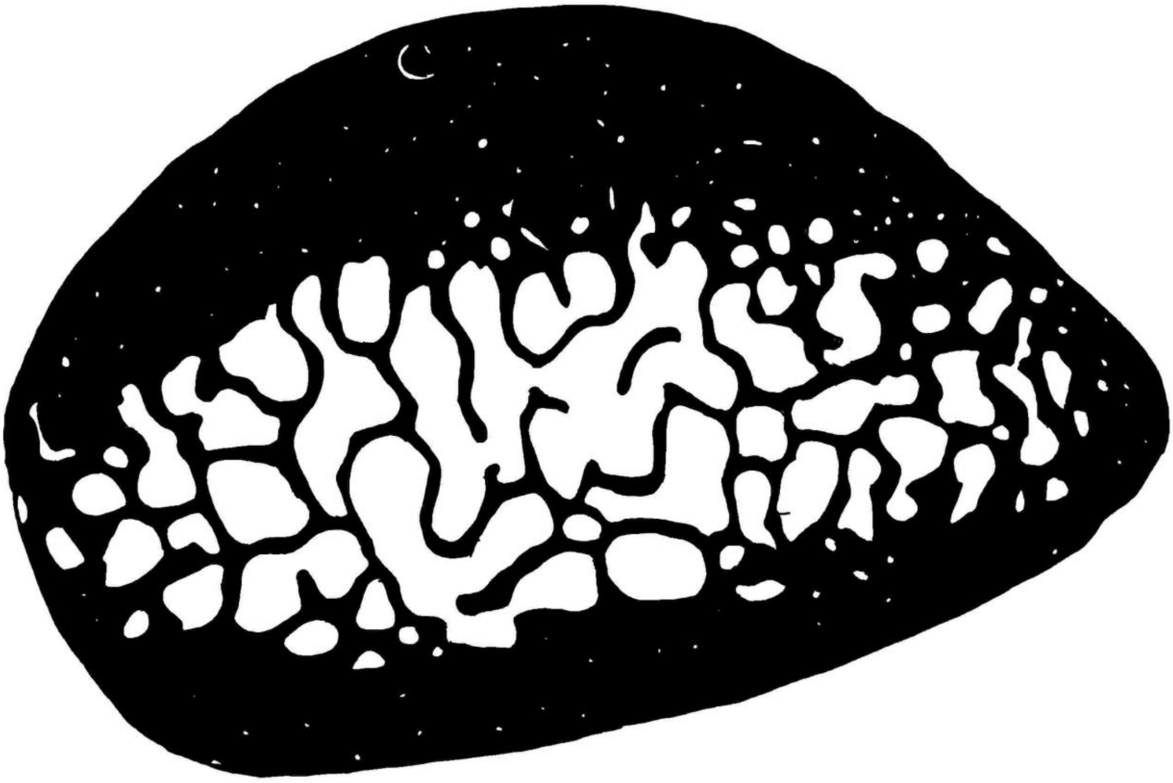

NHMO-DMA-42918 Distal

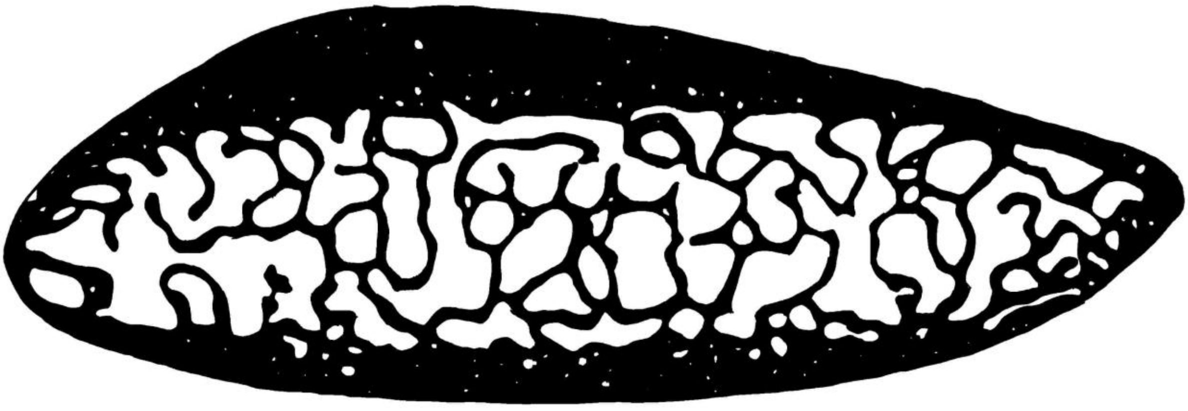

NHMO-DMA-32051 Proximal

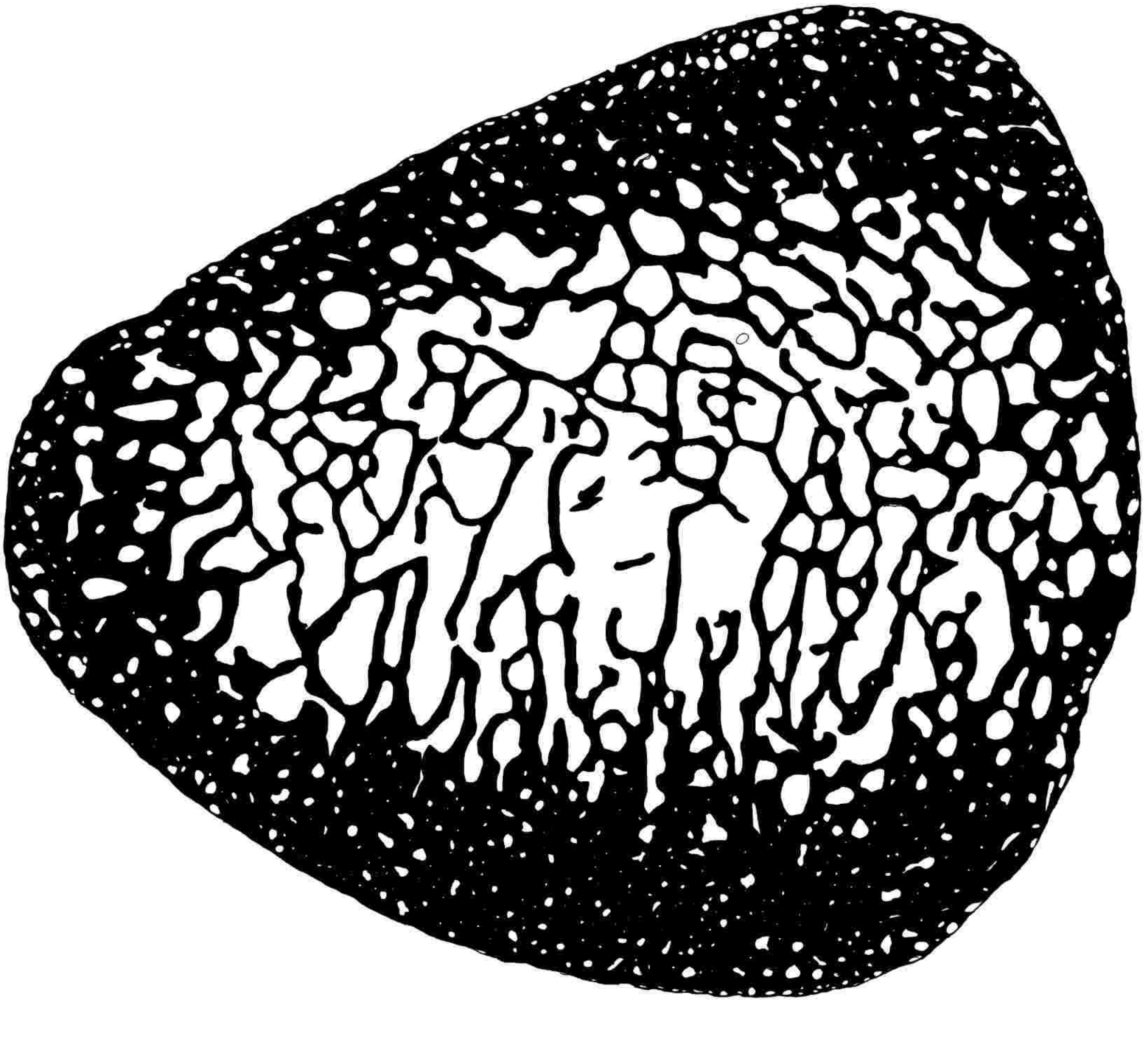

NHMO-DMA-32051 Mid-shaft

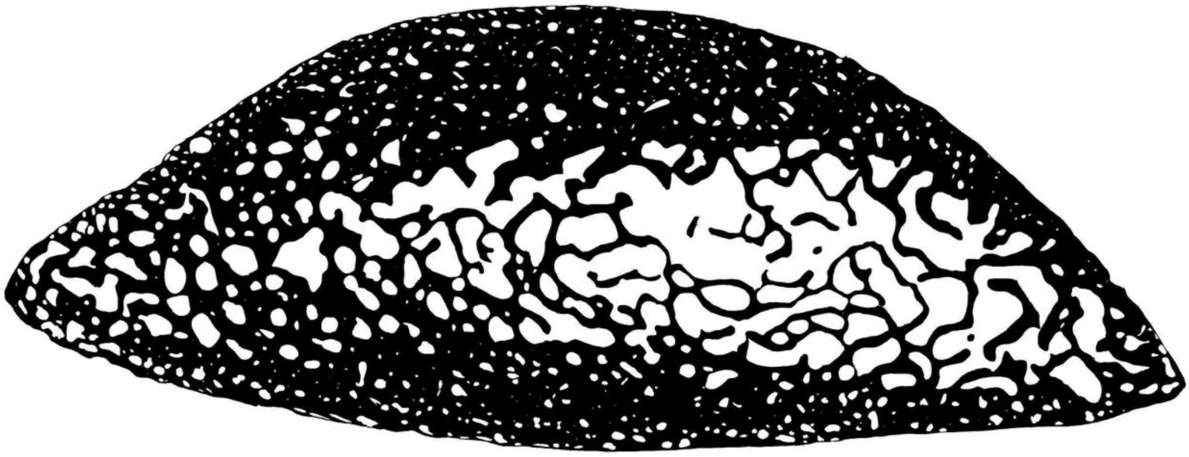

NHMO-DMA-32051 Distal

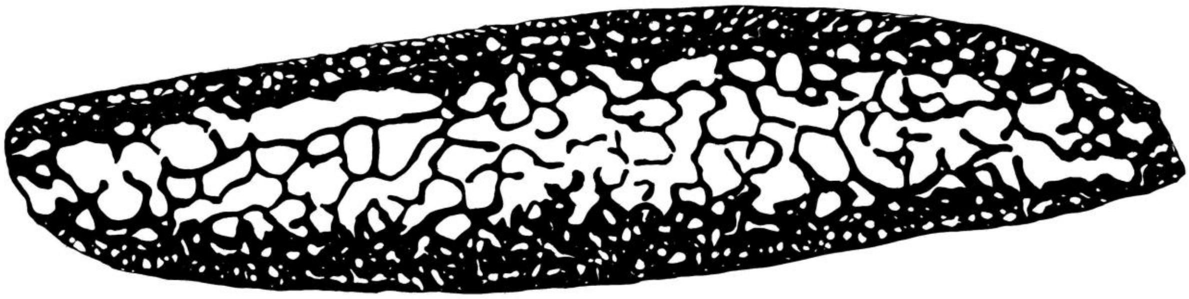

Supplement: Supplemental Information 3 — Raw data for BoneProfiler including binarized photos of all 12 rib sections (PMO 222.667, PMO 222.669, NHMO-DMA-42918, NHMO-DMA-32051). [file peerj-14-21486-s003.pdf]
