# Supplementary material for: Rib microstructure in thunniform ichthyosaurs and toothed whales
Source: PeerJ. 2026 Jul 7;14:e21486. doi: 10.7717/peerj.21486 (PMC13353231; doi:10.7717/peerj.21486)
Supplement: Supplemental Information 4 [file peerj-14-21486-s004.pdf]

Supplemental data S4 Binarized gastralia sections for data collection using BoneProfiler

PMO 222.667 gastralia

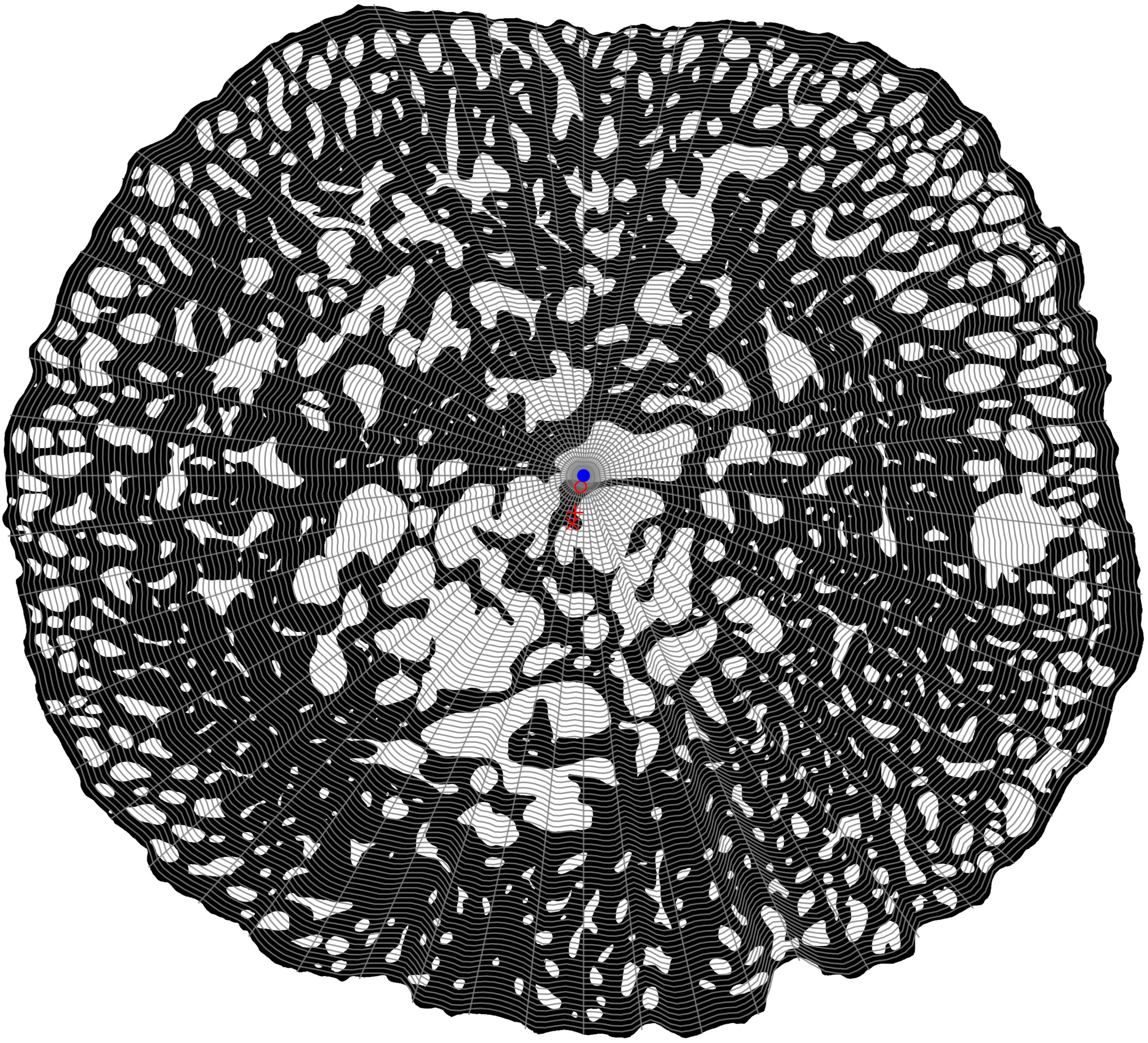

PMO 222.669 gastralia

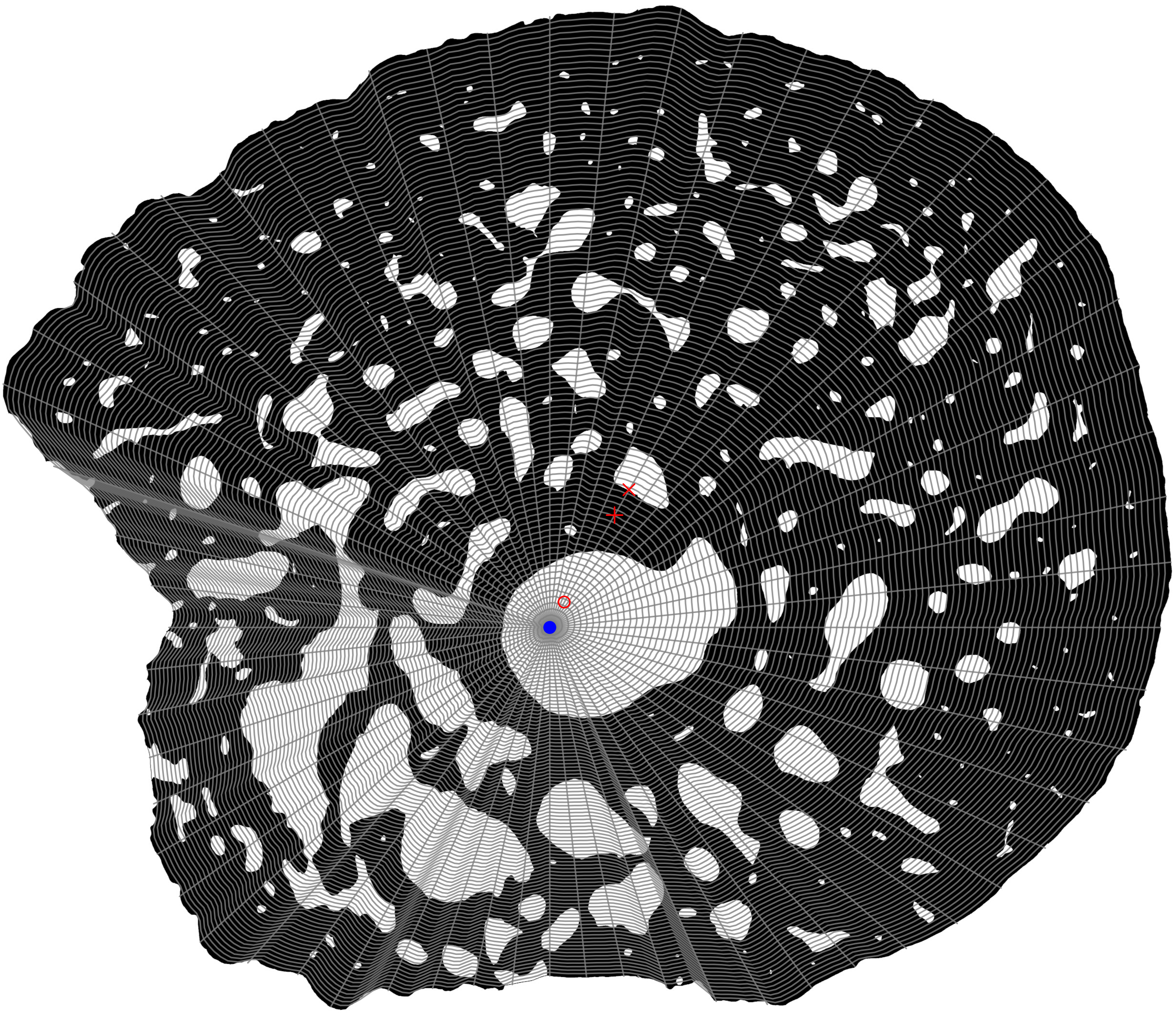

X Center of the mineralized part

O Center of the non−mineralized part

+ Center of the section

Background

Foreground
