# Supplementary figures and images for: Rib microstructure in thunniform ichthyosaurs and toothed whales
Source: PeerJ. 2026 Jul 7;14:e21486. doi: 10.7717/peerj.21486 (PMC13353231; doi:10.7717/peerj.21486)

## logistic

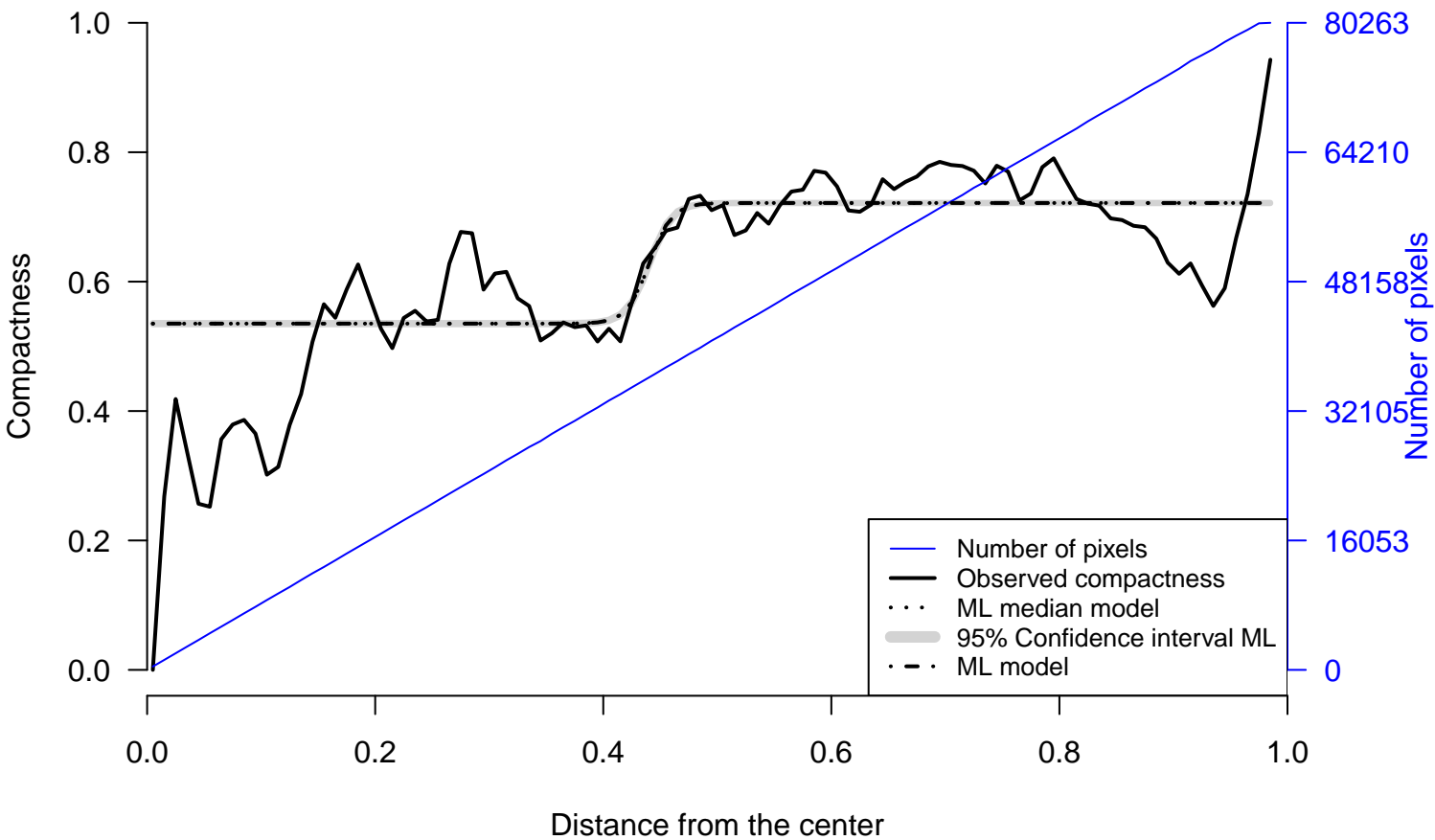

## flexit

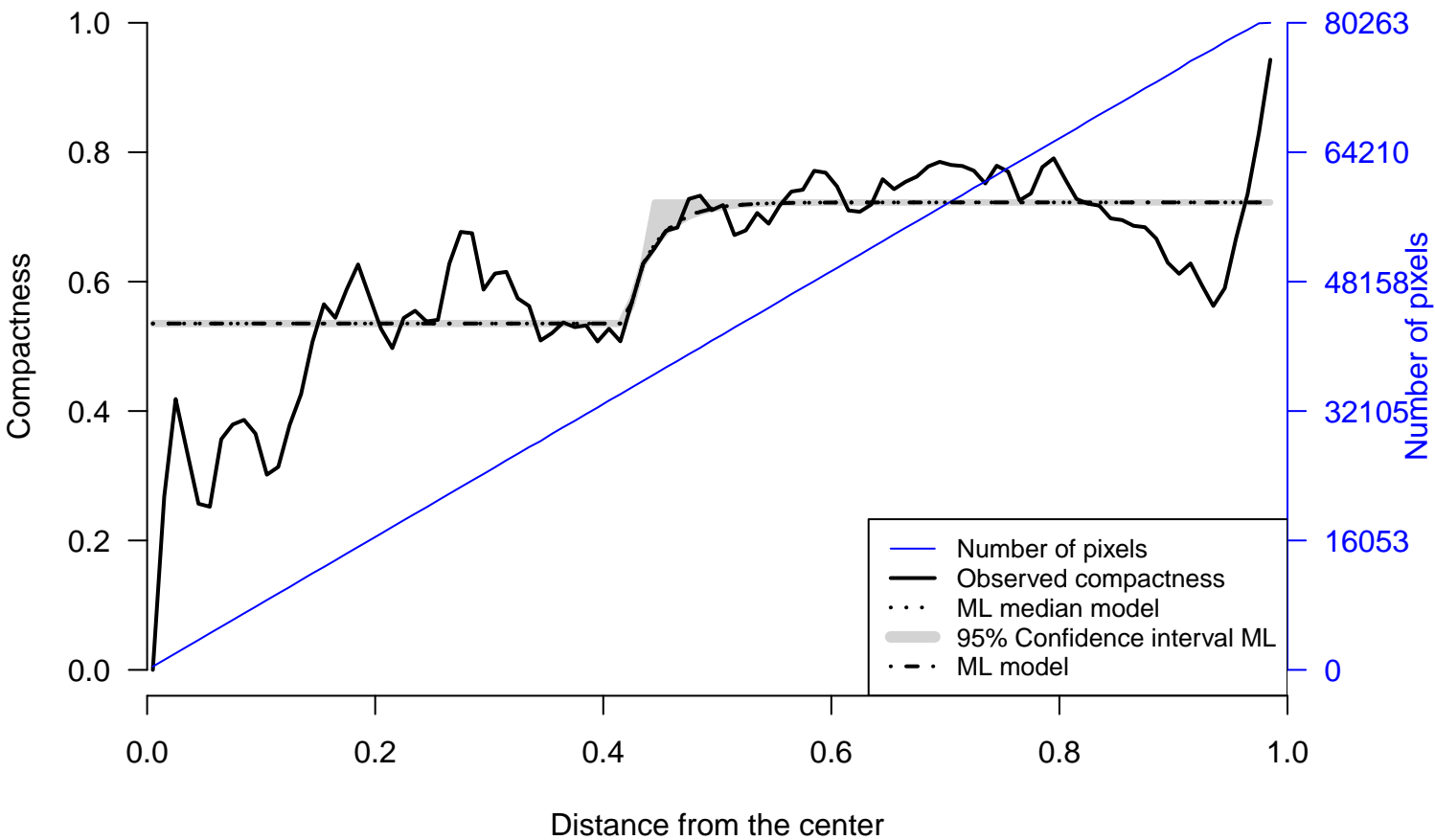

logistic

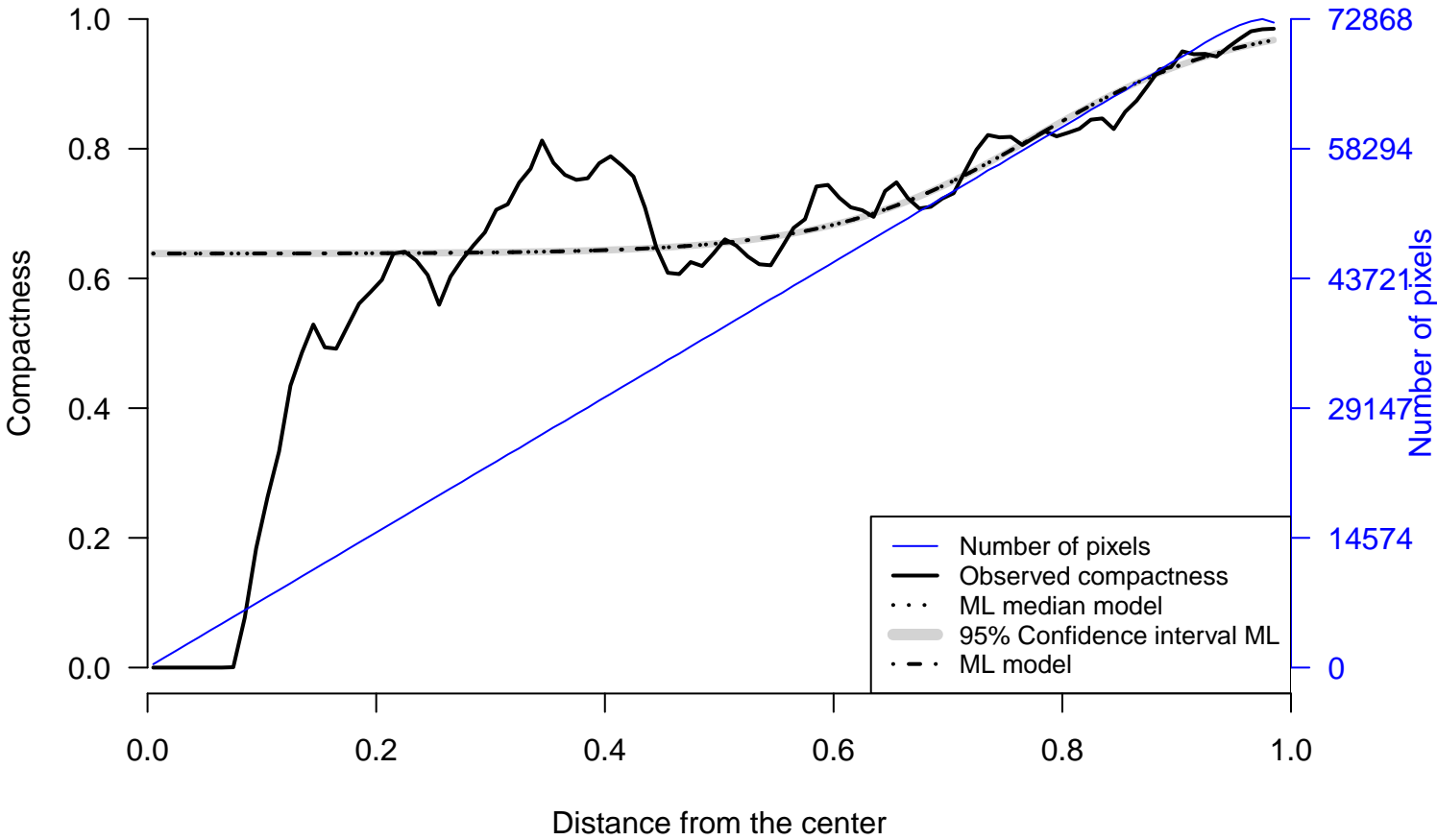

flexit

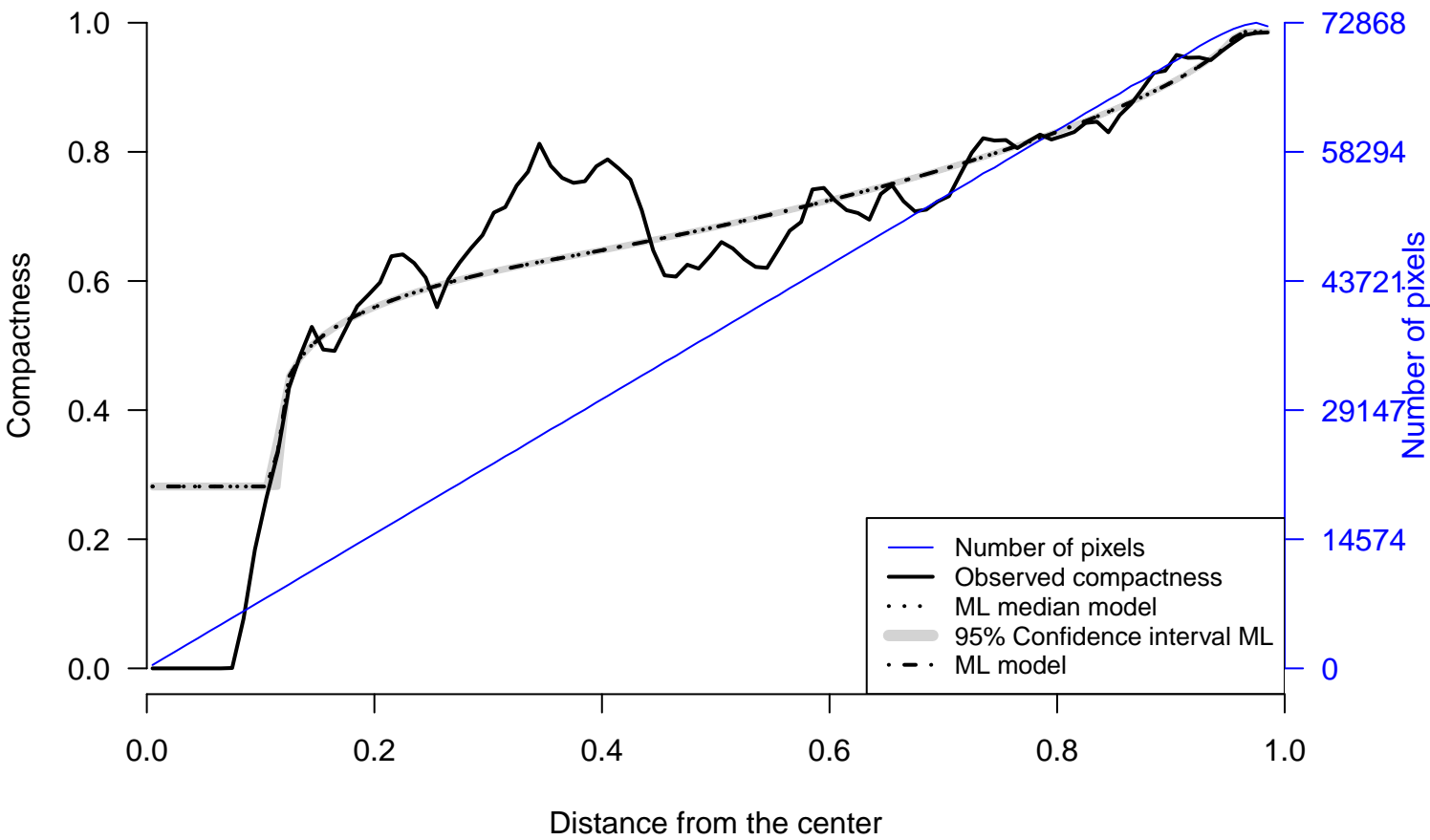

Supplement: Supplemental Information 5 [file peerj-14-21486-s005.pdf]
